# Prediction of HIV-1 protease cleavage site using a combination of sequence, structural, and physicochemical features

**DOI:** 10.1186/s12859-016-1337-6

**Published:** 2016-12-23

**Authors:** Onkar Singh, Emily Chia-Yu Su

**Affiliations:** Graduate Institute of Biomedical Informatics, College of Medical Science and Technology, Taipei Medical University, Taipei, Taiwan

**Keywords:** HIV-1 protease, Cleavage sites, Sequence features, Structural features, Physicochemical properties, Pseudo amino acid composition, Machine learning

## Abstract

**Background:**

The human immunodeficiency virus type 1 (HIV-1) aspartic protease is an important enzyme owing to its imperative part in viral development and a causative agent of deadliest disease known as acquired immune deficiency syndrome (AIDS). Development of HIV-1 protease inhibitors can help understand the specificity of substrates which can restrain the replication of HIV-1, thus antagonize AIDS. However, experimental methods in identification of HIV-1 protease cleavage sites are generally time-consuming and labor-intensive. Therefore, using computational methods to predict cleavage sites has become highly desirable.

**Results:**

In this study, we propose a prediction method in which sequence, structural, and physicochemical features are incorporated in various machine learning algorithms. Then, a bidirectional stepwise selection algorithm is incorporated in feature selection to identify discriminative features. Further, only the selected features are calculated by various encoding schemes and used as input for decision trees, logistic regression, and artificial neural networks. Moreover, a more rigorous three-way data split procedure is applied to evaluate the objective performance of cleavage site prediction. Four benchmark datasets collected from previous studies are used to evaluate the predictive performance.

**Conclusions:**

Experiment results showed that combinations of sequence, structure, and physicochemical features performed better than single feature type for identification of HIV-1 protease cleavage sites. In addition, incorporation of stepwise feature selection is effective to identify interpretable biological features to depict specificity of the substrates. Moreover, artificial neural networks perform significantly better than the other two classifiers. Finally, the proposed method achieved 80.0% ~ 97.4% in accuracy and 0.815 ~ 0.995 evaluated by independent test sets in a three-way data split procedure.

**Electronic supplementary material:**

The online version of this article (doi:10.1186/s12859-016-1337-6) contains supplementary material, which is available to authorized users.

## Background

### Introduction

In early 1980’s, human immunodeficiency virus (HIV) and acquired immune deficiency syndrome (AIDS) transition began in perishing modus with a leading cause of death. AIDS is an advanced stage infection with the HIV [1]. The first AIDS cases were reported in the United States in June 1981 by Center for Disease Control (CDC) [2]. Now it has been 35 years and still HIV is one of the major global public health issues. According to global HIV statistics, 36.9 million people had HIV and 1.2 million people died from AIDS-related illnesses (UNAIDS, 2015). However, after the confrontation with AIDS epidemic, unprecedented endeavors have been coordinated towards the advancement of antiretroviral treatments of AIDS that assault and repress the action of HIV-1 protease (HIV-1 PR).

HIV-1 protease is the principle etiologic agent of AIDS discovered by Gallo and coworkers in 1984 [3]. It is able to infect and destroy the human immune system, and allows life threating infection. HIV-1 PR, a homodimeric enzyme belonging to aspartate family also known as aspartyl retropepsin, plays a crucial role in viral maturation [4]. HIV constructs many of its protein in one long piece consisting of several tandemly linked proteins. HIV-1 PR has a responsibility to cleave Gag and Gag-Pol polyproteins into their component proteins responsible for the maturation of new virions, which can then infect new cells [5]. Thus, an HIV-specific protease is necessary for the HIV to make more functional viruses. Without HIV-1 PR, it is not possible for HIV to replicate due to unavailability of infectious virion and it remains uninfected. HIV-1 protease specifically binds with a precursor protein in octapeptide length, and cleaves it at the scissile bond represented as P4-P3-P2-P1↓P1′-P2′-P3′-P4′, where N-terminus as Pm (i.e., nonprime-side) and C-terminus as Pm’ (i.e., prime-side) in perceived octapeptide arrangement, though “↓” signifies a nearness of a scissile bond [5].

### Challenges of HIV-1 protease identification

HIV-1 PR is the key target of the most effective antiviral drugs for the treatment of HIV-1 infection as it processes the viral precursor Gag–Pol polyproteins, and allows maturation of the immature virion to make more functional viruses. However, the specificity of HIV-1 PR is partially understood because viral polyproteins do not share sequence homology and binding motifs between various substrates [5]. At present, researchers have partially succeeded to develop HIV protease inhibitors that are accessible for HIV treatment. However, they have conditional drawbacks such as poor bioavailability and excruciating infectiousness [6] that lead researchers to proceed with their endeavors to create novel and more potent compounds. Also, due to the tremendous amount of potential peptides, it is difficult to discover inhibitors by ordinary ways to deal with testing various types of peptides one by one, which is more labor-intensive and time-consuming.

### Previous work in HIV-1 protease cleavage site prediction

To conquer the difficulties to identify HIV protease, researchers are inclined towards in-silico approaches to predict HIV-1 protease cleavage sites [7]. In the past few years, several previous studies incorporated biological features with machine learning algorithms and gained better predictive performance compared to traditional approaches. You et al. [8] incorporated machine learning algorithms including artificial neural network (ANN) and support vector machine (SVM) to examine the specificity of an HIV-1 protease for the discovery and development of effective protease inhibitors. Kontijevkis et al. used an extensive dataset collected from HIV proteome research, and designed a rule-based predictive model on rough sets to analyze the specificity of HIV-1 protease [5]. Kim et al. organized their own datasets by compiling peptide sequences, and used a combination of neural networks and decompositional approaches to generate symbolic rules [9]. Ogul et al. used variable context markov chains (VCMC) to develop a generative model for HIV-1 cleavage specificity, and suggested that VCMC model is effective for prediction of cleavage sites of all proteases [10]. Nanni et al. developed a robust and reliable system in which genetic programming was used to design encoding techniques, and they showed the proposed ensemble method performed better than non-optimized SVM with standard encoding by cross-validation [11]. Jaeger et al. proposed a new fusion technique in which they added several classifiers including decision trees (DT), ANN, and SVM. They used cross-validation for evaluation and reported that the combined method achieved better performance than using a single classifier [12]. Kim et al. introduced a new feature selection method with multilayer perceptron (MLP) and used a decompositional approach to trained MLP. Li et al. developed a theoretical framework based on kernel methods to reduced dimensionality with linear support vector machine (LSVM) classifiers [13]. Newell proposed a new cascade detection algorithm to study the specificity on two datasets, and reported that the proposed method is useful in detection of multifactor synergies in several datasets [14]. Gök and Özcerit used OETMAP encoding schemes based on amino acid features together with linear classifiers. The encoding schemes improved prediction performance compared to standard amino acid encodings evaluated on two datasets by cross-validation [15]. Song and coworkers developed a protease specificity prediction server to predict unique substrates and their cleavage sites. They used support vector regression and bi-profile Bayesian feature extraction method to predict cleavage sites [16]. Niu et al. studied protease specificity correlation-based feature subset (CfsSubset) selection method combined with genetic algorithms [17]. Bozek et al. developed a model for structure-based prediction of HIV tropism and identification of important V3 loop properties for coreceptor usage [18]. Rögnvaldsson et al. proposed a method to combine linear support vector machine with orthogonal encoding schemes. They claimed that the model is effective for predicting cleavage sites by HIV-1 protease [19]. Liu et al. used feed forward back propagation neural network in their method along with feature selection schemes [20].

### Specific aims of this study

The advancement of reasonable HIV protease inhibitors can happen when we have a robust and suitable technique for anticipating the cleavage sites in proteins by HIV protease [7]. In this study, we propose a prediction method in which sequence, structural, and physicochemical features are incorporated in various machine learning algorithms. For feature selection, a bidirectional stepwise selection algorithm is incorporated to identify the discriminative features. Then the features are encoded by various encoding schemes and used as input for decision trees, logistic regression, and artificial neural networks. Moreover, a more rigorous three-way data split is applied to evaluate the objective performance of cleavage site prediction. The proposed HIV-1 protease specificity prediction method can further help the development of more potential HIV-1 protease inhibitors.

## Methods

### Datasets

In the present study, four benchmark datasets organized by Rögnvaldsson et al. [19] were used in our proposed method. The benchmark datasets are collections of octamers containing cleavage and non-cleavage sites as shown in Table 1. The 746, 1625, Schilling, and Impens datasets contain 746 (401 cleaved and 345 non-cleaved), 1625 (374 cleaved and 1251 non-cleaved), Schilling (434 cleaved and 2838 non-cleaved), and Impens (149 cleaved and 798 non-cleaved) octamers, respectively. The datasets are available in the supplementary material [Additional files 1, 2, 3 and 4].

**Table 1:** Four benchmark datasets for HIV-1 cleavage site prediction

Datasets	Octamers	Cleavage sites	Non-cleavage sites
746	746	401	345
1625	1625	374	1251
Schilling	3272	434	2838
Impens	947	149	798

### Feature extraction

Amino acids are the essential components of peptides and proteins, and each of 20 amino acids has unique but different properties. The combination of the properties of various residues within a protein can influence diversification and characteristics of the protein structure and function. The aim of the study is to develop a better prediction model using various combinations of features that can predict the HIV-1 protease cleavage sites. The present investigation involved extraction of three different kinds of features based on sequence, structure, and physicochemical properties. Several feature extraction methods in propy 1.0 software package [11] were employed to extract sequence-based and physicochemical-based features. For structure-based feature extraction, NetSurfP [21] web server was used. Besides, we considered AAindex [22] database for physicochemical properties. This database contains numerical indices which represent several physicochemical and biochemical properties of amino acids and amino acid pairs.

#### Sequence-based features

Sequence-based features include the composition of amino acids which contains 20 factors with each representing the occurrence frequency of one native amino acid in a given peptide. The selected sequence based features are amino acid composition (AAC), dipeptide composition (DipC), pseudo amino acid composition (PseAAC) [23]. AAC and its variations have been demonstrated that they are influential in predicting HIV-1 protease cleavage sites. Besides, protease has a preference for some amino acid compositions at their cleavage sites. For example, trypsin recognizes essential amino acid lysine and arginine and cleaves at carboxyl terminal. Afterward, the DipC was selected to represent occurrence frequencies of amino acid pairs in peptides. At last, the important point about PseAAC is that it is endowed with the information about AAC and also contains information beyond it, and hence can better reflect the features of peptides through a discrete model. In our study, 20 and 400 variables are used to represent AAC and DipC, respectively. Another 25 variables were utilized for PseAAC by propy 1.0 package.

#### Structure-based features

Structure-based features are important to study the substrate specificity of the HIV-1 protease with the aim of obtaining a better differentiation between cleavage and non-cleavage sites. Also, it was stated in statistics for structural and sequence comparisons of protein pairs that the structural comparison can explore almost double as many different relationships as sequence comparison [24]. In this work, two structure-based features including solvent accessibility (SA) and secondary structure elements (SSE) were selected. Through solvent accessibility, we can depict the exposed surface of the entire protein or individual amino acid. The significance of proper surface presentation of cleavage sites in the solvent-exposed region for efficient proteolysis is well evidenced. We thus predicted solvent accessibility using NetSurfP web server, and three columns were selected to represent the accessibility of a peptide, including buried or exposed (B/E) class, relative solvent accessibility (RSA), and absolute surface accessibility (ASA) for each residue in a peptide. Another structure-based feature we used in this study was the secondary structure which is characterized by folding of a peptide chain into the *α*-helixes, *β*-sheets, or random coils. The caspase substrate analysis indicated the considerable proportion of the cleavage sites located in *α*-helixes and *β*-sheets [16]. NetSurfP web server [21] was used for the secondary structure prediction, and generated three columns of probabilities for *α*-helix, *β*-sheet, and random coil for each residue in a peptide.

#### Physicochemical property features

Each peptide and proteins are the combinations of twenty amino acid components. These amino acids have common constituents of the amine groups, carboxyl groups, and side chains which have several functional groups, and these functional groups are responsible for distinct physical properties of each amino acid. In the study, we selected six physicochemical properties including hydrophobicity, polarizability, steric property, isoelectric point, volume, and polarity. Hydrophobicity is a physical property of amino acids representing the tendency of water to exclude non-polar molecules. Moreover, as stated earlier in previous studies, the hydrophobic nature of cleavage sites can efficiently bind with the substrates by Van der Waals interaction and help identify cleavage and non-cleavage sites in peptides [17]. The ability to form instant dipoles known as polarizability, through which the dynamical response of the closed system to external fields can be determined, and provide perception about a molecule’s internal structure can also be made [25]. Steric properties can be appraised by the attributes of an atom within the molecule. The overlapping electron clouds lead to the repulsion when the atoms are brought close to each other. A steric property encompasses various effects such as steric hindrance, steric shielding, steric attractions, chain crossing, and steric inhibition of resonance. These properties are largely responsible for the shapes (i.e., conformation) of molecules and also reactivity [26]. The isoelectric point can be defined as the pH at which amino acid is neutral [27]. Volume, a standard feature of native protein structures, is the dense packing of amino acid residues within interior regions, and a key parameter in understanding packing is the volume that individual amino acid residue occupies in different environments [28]. Polarity confers molecules and compounds with distinctive features regarding the structure and chemical interaction with other molecules. Due to this property, polar amino acids are exposed on the surface of proteins [29].

### Machine learning algorithms

In this study, three algorithms have been applied to predict the HIV-1 protease specificity, including DT, ANN, and logistic regression (LR). The sequence, structural, and physicochemical features are incorporated as inputs for DT, ANN, and LR to investigate discriminative biological features and construct an accurate predictive model. The descriptive and predictive modeling provides insights that drive better decision-making. Keeping this in mind, our research group was motivated to use SAS Enterprise Miner Workstation 13.2, having the suite of machine learning algorithms that enables to create accurate predictive and descriptive model. It also allowed us to compare several predictive models simultaneously. ANN, a machine learning approach resembling the biological neural network especially human brain, is fabricated to mimic the structure and function of our nervous system. It scores over the conventional rule-based programming owing to its broad applicability for the various tasks such as classification, sequence recognition, and novelty detection [24]. The important aspect about ANN is a non-parametric model while most statistical methods are parametric models that need the higher background of statistics. Moreover, ANN generates models to detect non-linear interactions between dependent and independent variables. DT is a simple yet effective machine learning algorithm to yield interpretable results with numerous conceivable results. It orders examples by shorting them down the tree from the root hub to leaf hub to arranging the cases [30]. There is a distinct advantage of applying decision trees to classify biomedical problems that make DT better predictors among others. The best characteristic of using trees is very intuitive and easy to explain. In addition, variable nonlinearity usually results in poor predictive performance while using other classifiers such as simple regressions. Another advantage of DT is that nonlinearity property in data does not influence the predictive performance of DT. Therefore, DT predictors can be applicable to data with nonlinear relationships. On the other hand, LR is a machine learning algorithm where the dependent variable is categorical. It calculates the probability of categorical dependent variable and other independent variables. The most important point of LR is that the expected values of response variables are modeled based on the combination of values taken by the predictors [31]. Our motivation to use LR is a white-box model that allows an interpretation of model parameters. It gives real probabilities of predicted class unlike DT and SVM and it is easier to update the model to take in new data with the help of online gradient descendent method.

### System architecture

**Fig. 1:**
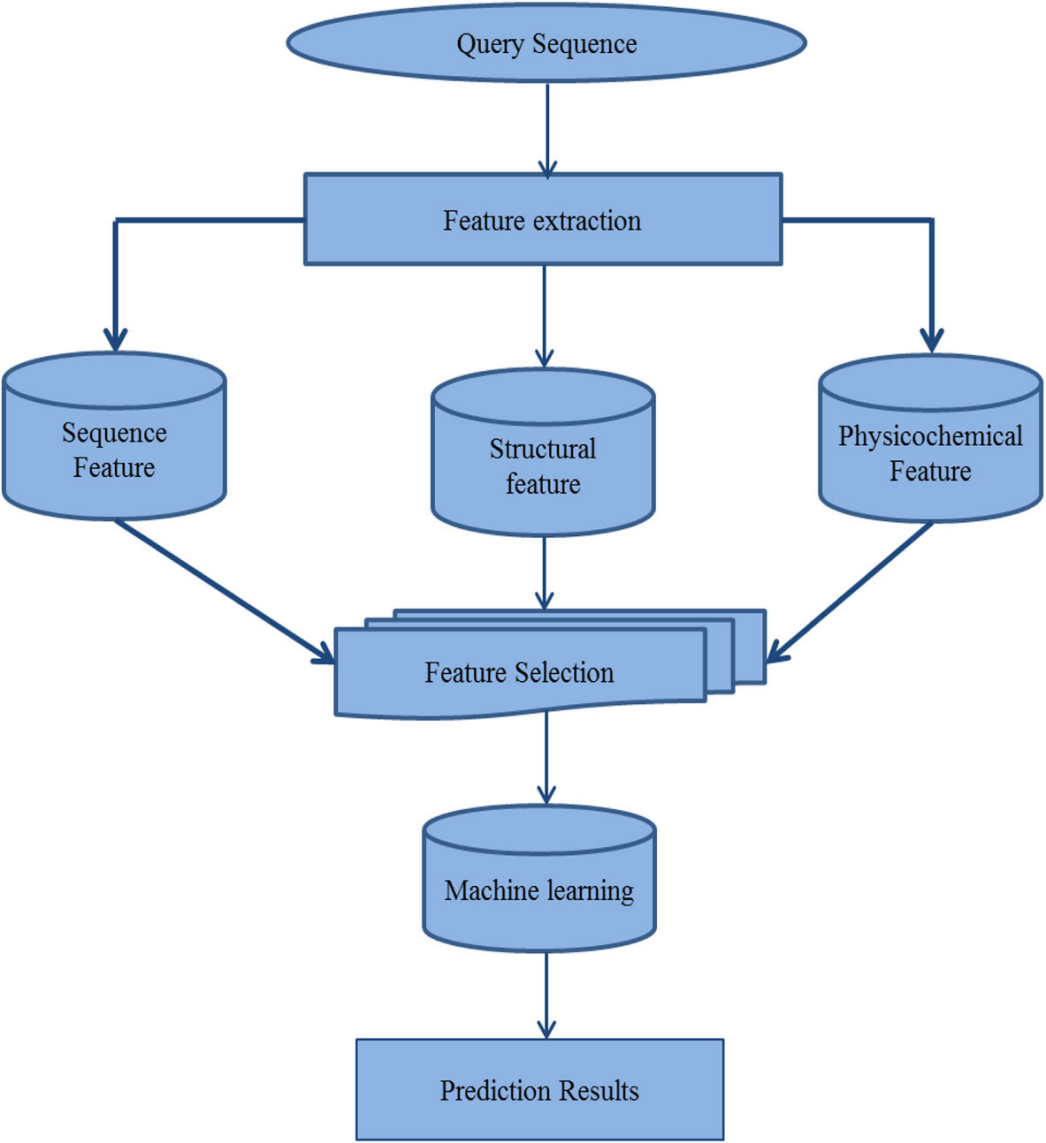
System architecture of the proposed method

The system architecture of our proposed method for predicting cleavage sites in HIV-1 protease is illustrated in Fig. 1. The analysis workflow involves several steps, including query protein peptide input, feature extraction, feature selection, machine learning algorithms, and the prediction results. First, octamers are extracted from query protein sequences by a sliding window of size eight. Then, the sequence features, structural features, and physicochemical properties were extracted and encoded with the aid of propy 1.0 software package. Further, a bidirectional stepwise selection algorithm is incorporated to select only the discriminative biological features as input to be submitted to machine learning algorithms for prediction. At last, examination of all models was made, and the execution of prediction model was illustrated. The proposed method is named as ProCleSSP (Protease Cleavage site prediction based on Sequence, Structural, and Physicochemical features).

### Evaluation measures

Model assessment is critical in regards to measuring the nature of predictions. In our experiments, we incorporated a three-way data split procedure to avoid overfitting and overestimation, and randomly divide our raw data into 90% as the training set, 5% as the validation set, and 5% as the test set. The training set is used to train a predictive model. The validation dataset is applied for feature selection and parameter tuning. The test dataset is incorporated as an independent set only to evaluate the real performance of a prediction method. As for the evaluation measures, we used accuracy and area under the receiver operating characteristics (ROC) curve to compare with other previous studies. The accuracy (*Acc*.) of a prediction method is calculated as the summation of true positives and true negatives divided by the total number of data. In addition, the area under the ROC curve (AUC) is used to assess performance during parameter selection, and is one of the most appropriate measures of performance as it is non-parametric and threshold independent. It is calculated from ROC curve which is a fundamental tool for diagnostic test evaluation. In an ROC curve, the true positive rate (i.e., sensitivity) is plotted in function of the false positive rate (i.e., 1-specificity) for different cutoff points of a parameter. In our study, we use AUC as an evaluation measure to select a combination of effective features and classifiers. Other metrics are also computed to allow more comprehensive evaluation of predictive performance. Sensitivity (*Sen.*) and specificity (*Spe.*) measure how well a classifier detects cleavage sites as cleavage sites and non-cleavage sites as non-cleavage sites, respectively. The following equations define these statistics, where *TP*, *TN*, *FP*, and *FN* denote the numbers of true positives, true negatives, false positives, and false negatives, respectively.

$$ \begin{array}{c}\hfill Sen.=\frac{TP}{TP+FN}\hfill \\ {}\hfill Spe.=\frac{TN}{TN+FP}\hfill \\ {}\hfill Acc.=\frac{TP+TN}{TP+TN+FP+FN}\hfill \end{array} $$

## Results and discussion

In ProCleSSP, the biological features are extracted from sequence-based, structure-based, and physicochemical properties. Then the extracted biological features from the four benchmark datasets (i.e., 746, 1625, Schilling, and Impens) are used as input features to three machine learning algorithms (i.e., ANN, DT, and LR), and predictive performance are optimized by AUC based on the validation set instead of the test set to avoid overfitting. Here, to compare the effects of various biological features, the predictive performance is analyzed by single feature type prediction and hybrid feature type prediction. For single feature type prediction, the performance of sequence-based features, structure-based features, and physicochemical properties are compared. In addition, the hybrid feature type prediction are conducted by the combination of various feature types, including sequence and structure features, sequence and physicochemical features, structure and physicochemical features, and all three types of features.

### Prediction performance based on single feature types

In our experiment, the effects of different biological features are compared individually. The prediction performance based on sequence features, structural features, and physicochemical features are detailed in the following sections.

#### Sequence-based features

Three types of sequence-based features (i.e., AAC, DipC, and PseAAC) are used to depict the effect of using sequence patterns to distinguish cleavage sites from non-cleavage sites. The predictive performance based on sequence features for the four benchmark datasets is shown in Table 2. We compare the accuracy and AUC of different algorithms based on AAC, DipC, PseAAC, and the combination of all three compositions. Experiment results show that incorporation of DipC performed better than using AAC or PseAAC itself. This suggests that DipC could be a better indicator to predict HIV-1 protease cleavage sites due to its capability to consider pairwise amino acid pair relationships. For the machine learning algorithms, ANN achieved better predictive performance except for the AUC of the Schilling dataset.

**Table 2:** Predictive performance of sequence features for the four benchmark dataset

Features	DT		LR		ANN	
	*Acc.*(%)	AUC	*Acc.*(%)	AUC	*Acc.*(%)	AUC
746 Dataset						
AAC	83.7	0.897	86.4	0.938	81.0	0.935
DipC	75.6	0.793	86.4	0.865	91.9	0.974
PseAAC	78.3	0.787	86.4	0.938	81.0	0.885
Seq_All	78.3	0.831	86.4	0.847	91.9	0.979 ^*^
1625 Dataset						
AAC	91.4	0.908	84.1	0.904	91.4	0.952
DipC	92.6	0.861	96.3	0.972	98.7	0.987
PseAAC	90.2	0.822	87.8	0.921	87.8	0.945
Seq_All	92.6	0.882	96.3	0.958	98.7	0.984
Schilling Dataset						
AAC	87.7	0.664	86.5	0.856	88.9	0.858
DipC	87.7	0.526	87.1	0.806	89.5	0.790
PseAAC	87.1	0.500	86.5	0.864	88.3	0.858
Seq_All	87.7	0.611	87.7	0.802	87.1	0.821
Impens Dataset						
AAC	85.1	0.500	80.8	0.857	89.3	0.886
DipC	85.1	0.500	82.9	0.579	93.6	0.893
PseAAC	87.2	0.721	78.7	0.814	87.2	0.868
Seq_All	87.2	0.802	85.1	0.696	89.3	0.875

^*^The best accuracy and AUC in each dataset are underlined

#### Structure-based features

Two structure-based features, SA and SSE, were incorporated individually or combined together to identify cleavage sites in our study. For solvent accessibility, we used three descriptors, including solvent accessibility class (i.e., exposed or buried), RSA, and ASA. For secondary structure, the probability of *α*-helix, *β*-sheet, and random coil are predicted by the NetSurfP web server. An octapeptide generates 24 descriptors for each of solvent accessibility and secondary structure features. The predictive performance using structural features for the four benchmark data sets is shown in Table 3. The results indicate that SA usually performed better than SSE when it is used individually or combined with SSE. This lends support on our assumption that the cleavage sites usually occur on the surface of a molecule and thus SA serves as an effective factor to identify cleavage sites in HIV-1 protease. When we compare machine learning algorithms, ANN also performed better than the others.

**Table 3:** Predictive performance of structural features for the four benchmark datasets

Features	DT		LR		ANN	
	*Acc.*(%)	AUC	*Acc.*(%)	AUC	*Acc.*(%)	AUC
746 Dataset						
SSE	62.1	0.626	59.4	0.715	78.3	0.838
SA	83.7	0.791	78.4	0.771	81.0	0.771
Str_All	83.7	0.791	70.2	0.806	78.4	0.897 ^*^
1625 Dataset						
SSE	81.7	0.756	76.8	0.673	85.3	0.742
SA	91.4	0.920	89.0	0.961	96.3	0.977
Str_All	91.5	0.920	85.4	0.936	89.0	0.935
Schilling Dataset						
SSE	87.1	0.500	88.3	0.775	88.3	0.800
SA	89.5	0.788	84.0	0.828	87.1	0.840
Str_All	89.5	0.788	83.4	0.824	85.8	0.843
Impens Dataset						
SSE	85.1	0.500	85.1	0.729	87.2	0.761
SA	89.3	0.736	89.3	0.918	95.7	0.950
Str_All	87.2	0.571	89.3	0.857	89.3	0.914

^*^The best accuracy and AUC in each dataset are underlined

#### Physicochemical features

In physicochemical properties, six properties including hydrophobicity, polarizability, steric properties, isoelectric point, volume, and polarity are incorporated to detect cleavage sites. Each property was encoded as 25 descriptors by PseAAC using propy 1.0 software package. The physicochemical properties were examined individually as well as in combinations. The predictive performance based on physicochemical properties for the benchmark datasets is shown in Table 4.

**Table 4:** Predictive performance of physicochemical property features for the four benchmark datasets

Features	DT		LR		ANN	
	*Acc.*(%)	AUC	*Acc.*(%)	AUC	*Acc.*(%)	AUC
746 Dataset						
Hydrophobicity	75.6	0.735	83.7	0.956	89.1	0.968 ^*^
Steric property	89.1	0.929	86.4	0.941	81.0	0.932
Polarizability	81.0	0.815	83.7	0.953	83.7	0.947
Isoelectric point	81.0	0.865	86.4	0.953	83.7	0.953
Polarity	83.7	0.838	83.7	0.912	86.4	0.909
Volume	83.7	0.838	54.0	0.500	54.0	0.500
Phy_All	84.9	0.882	93.6	0.885	97.3	0.953
1625 Dataset						
Hydrophobicity	87.8	0.849	84.1	0.896	86.5	0.874
Steric property	91.4	0.897	85.3	0.896	91.4	0.934
Polarizability	93.9	0.914	87.8	0.936	96.3	0.957
Isoelectric point	91.4	0.918	82.9	0.914	93.9	0.968
Polarity	86.5	0.847	87.8	0.904	89.0	0.919
Volume	92.6	0.896	89.0	0.933	93.9	0.974
Phy_All	92.7	0.882	92.7	0.921	92.7	0.944
Schilling Dataset						
Hydrophobicity	87.7	0.708	89.5	0.862	89.5	0.863
Steric property	88.3	0.721	86.5	0.837	88.3	0.843
Polarizability	89.5	0.683	89.5	0.854	90.8	0.853
Isoelectric point	88.3	0.733	87.7	0.858	89.5	0.860
Polarity	87.1	0.500	87.1	0.860	88.3	0.865
Volume	88.9	0.622	88.3	0.847	88.3	0.810
Phy_All	88.9	0.593	89.5	0.876	85.2	0.863
Impens Dataset						
Hydrophobicity	85.1	0.500	80.8	0.686	87.2	0.886
Steric property	89.3	0.845	82.9	0.825	89.3	0.893
Polarizability	85.1	0.500	85.1	0.864	89.3	0.943
Isoelectric point	85.1	0.500	78.7	0.850	93.6	0.982
Polarity	85.1	0.500	85.1	0.743	82.9	0.682
Volume	85.1	0.500	85.1	0.736	80.8	0.500
Phy_All	91.5	0.839	82.9	0.796	87.2	0.839

^*^The best accuracy and AUC in each dataset are underlined

### Prediction performance based on hybrid feature types

In our study, the prediction performance based on hybrid features is undertaken. Hybrid features denote the combinations of sequence, structure, and physicochemical features. The combinations of such features could contain more extensive information than the single feature types. In this section, four distinct combinations are used to explore the impact of the properties in protease cleavage site prediction. First, sequence and structure features are combined together to check whether these properties influenced the cleavage sites. The number of features obtained for this combination is 493 (i.e., 445 for sequence and 48 for structure features). Secondly, sequence and physicochemical features were consolidated, a total of 595 features were combined (i.e., 445 for sequence features and 150 physicochemical properties). Thirdly, structure and physicochemical features are combined and generated 198 features (i.e., 48 for structure and 150 for physicochemical features). The last combination is to combine all three feature types together, and yields a total of 643 features. The predictive performance of the four combinations for the benchmark dataset is illustrated in Table 5. For the feature combinations, it is frequently observed that combining multiple features together can compensate the properties of various biological features and further improve the predictive performance in terms of both accuracy and AUC. The only exception is the Impens dataset in which incorporation of solvent accessibility performed slightly better than any combination from Table 5. In addition, it is also interesting to observe that ANN and LR perform significantly better than DT when different types of features are combined as input for prediction of cleavage sites. This suggests that incorporation of more advanced machine learning algorithms, such as ANN, could be a better choice to identify discriminative features from heterogeneous data.

**Table 5:** Predictive performance of hybrid features for the four benchmark datasets

Features	DT		LR		ANN	
	*Acc.*(%)	AUC	*Acc.*(%)	AUC	*Acc.*(%)	AUC
746 Dataset						
Seq + Str	78.3	0.788	91.8	0.982	94.5	0.994 ^*^
Seq + Phy	83.7	0.838	86.4	0.968	94.5	0.976
Phy + Str	83.7	0.810	78.3	0.860	91.8	0.982
Seq + Str + Phy	75.6	0.841	97.2	0.991	97.2	0.988
1625 Dataset						
Seq + Str	89.0	0.910	96.3	0.980	95.1	0.992
Seq + Phy	89.0	0.785	97.5	0.958	98.7	0.990
Phy + Str	91.4	0.940	86.5	0.810	93.9	0.985
Seq + Str + Phy	91.4	0.956	95.1	0.980	97.5	0.990
Schilling Dataset						
Seq + Str	90.8	0.845	86.5	0.865	92.0	0.873
Seq + Phy	87.1	0.500	90.8	0.837	88.9	0.825
Phy + Str	85.1	0.500	80.8	0.603	80.8	0.596
Seq + Str + Phy	88.9	0.810	89.5	0.826	91.4	0.895
Impens Dataset						
Seq + Str	89.3	0.682	89.3	0.918	93.6	0.918
Seq + Phy	91.4	0.839	87.2	0.889	91.4	0.896
Phy + Str	85.1	0.500	82.9	0.889	93.6	0.932
Seq + Str + Phy	87.2	0.675	87.2	0.889	89.3	0.850

^*^The best accuracy and AUC in each dataset are underlined

### Best combinations of features and algorithms for each dataset

In our experiment, we used the AUC of the validation dataset to select a best combination of features and algorithms for each dataset, and then incorporated the test set to show the objective performance of cleavage site prediction in HIV-1 protease. The best combinations of features and algorithms for each dataset are listed in Table 6 and the ROC plots are shown in Figure S1–S4 of the supplementary material [Additional file 5]. Experiment results show that ProCleSSP achieved AUC of 0.994, 0.992, 0.895, and 0.950 based on validation sets for the 746, 1625, Schilling, and Impens datasets, respectively. We also attain accurate prediction accuracy of 94.5%, 95.1%, 91.4%, and 95.7% for the 746, 1625, Schilling, and Impens datasets, respectively. The sensitivity and specificity range from 57.1% ~ 100% and 88.2% ~ 100% for the validation sets, respectively. This suggests that imbalanced datasets for cleavage site identification could result in the observation that our method achieves higher specificity compared to sensitivity. However, if an independent test set, which has not been used to construct the classifier or tune features and parameters, is incorporated to evaluate the most objective performance of the prediction method, our results demonstrated that the performance could often be overestimated. For the feature selection, our method suggested that the best feature set is the combination of sequence and structural features together for the 746 and 1625 datasets. For the largest Schilling dataset, incorporation of all sequence, structural, and physicochemical features performed the best. For the Impens dataset, our validation results select SA as the best set of feature. However, it is interesting to notice that there is a large difference between validation and test performance. This might be resulted from the fact that the number of SA features is much smaller than others and thus this instability could lead to inadequate for prediction. On the other hand, ANN performed consistently better than the other two machine learning algorithms for prediction of HIV-1 protease cleavage sites. We incorporated a rigorous three-way data split procedure to prevent overfitting in our experiments, while most previous studies incorporated internal validation or cross-validation for performance evaluation. For example, ProCleSSP achieved slightly better performance (i.e., 95.1% in accuracy and 0.992 in AUC) in the 1625 dataset compared to performance (i.e., 93.0% in accuracy and 0.940 in AUC) in Kontijevskis et al. In addition, when compared with state-of-the-art method by Rögnvaldsson et al., their approach performed better than our method. Although ProCleSSP only attains comparable or slightly better performance compared with other approaches, a more objective performance of cleavage site prediction is illustrated in the proposed method.

**Table 6:** Predictive performance based on selected features and machine learning algorithms based on validation sets and test sets

Datasets	Features	Algorithm	*Sen.*(%)	*Spe.*(%)	*Acc*.(%)	AUC
746	Seq + Str	ANN	100.0 (100.0)^*^	88.2 (94.4)	94.5 (97.4)	0.994 (0.995)
1625	Seq + Str	ANN	94.7 (89.4)	95.2 (96.8)	95.1 (95.1)	0.992 (0.994)
Schilling	Seq + Str + Phy	ANN	57.1 (27.3)	96.5 (95.8)	91.4 (86.6)	0.895 (0.815)
Impens	SA	ANN	71.4 (44.4)	100.0 (89.8)	95.7 (80.0)	0.950 (0.816)

^*^The predictive performance of test set is shown in parenthesis

### Interpretable biological features for cleavage site identification

**Fig. 2:**
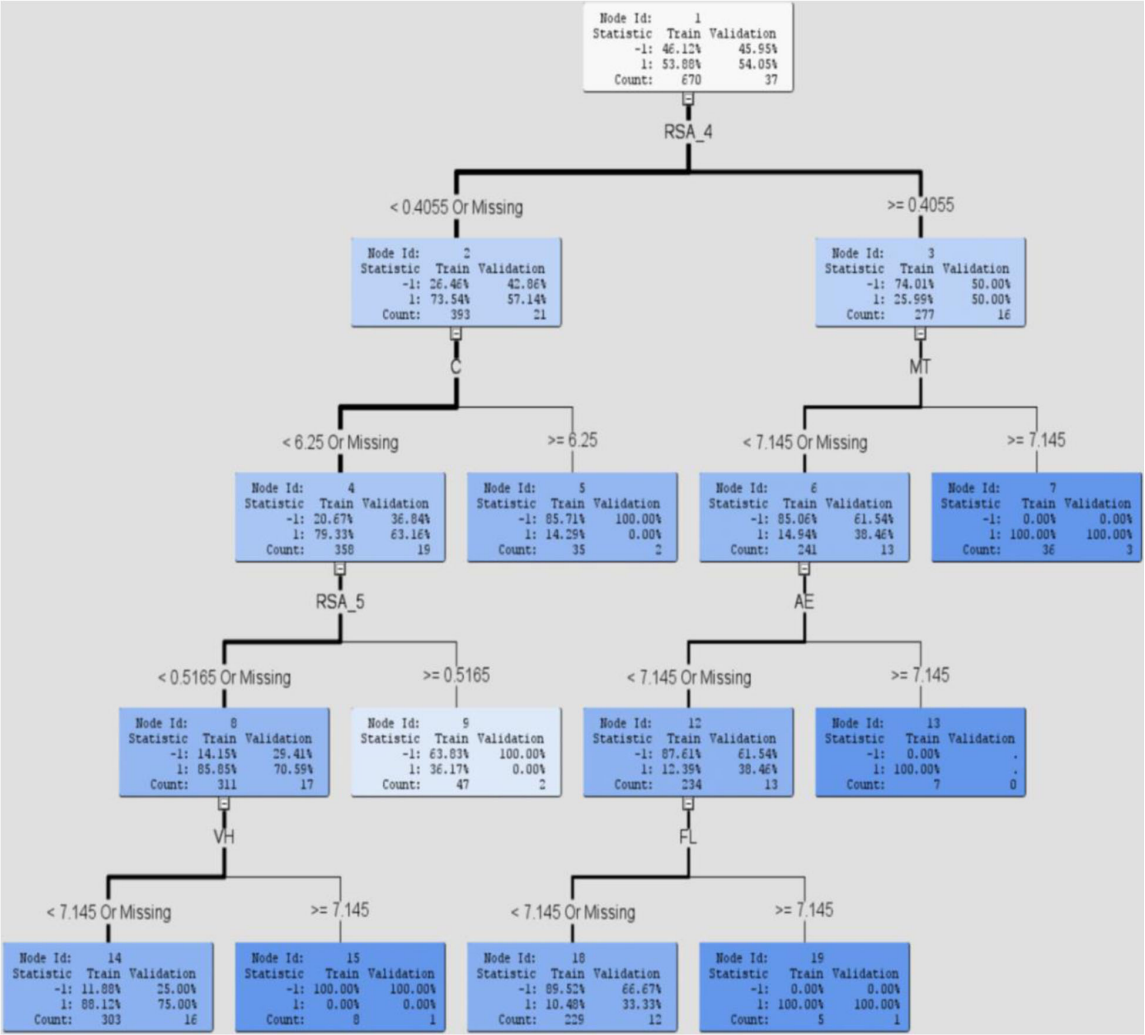
Decision tree of the 746 dataset based on Seq + Str features

In the proposed method, we incorporated several machine learning algorithms to predict cleavage sites. Although it has been demonstrated that ANN achieved the best predictive performance, discriminative biological features for cleavage sites can be interpretable by algorithms such as DT or LR. The interpretable models give a closed form of approximation of variables where the importance of each variable is explicit. Here, we draw attention to the decision tree model and variable importance for each dataset. In Fig. 2, the decision tree model for the 746 dataset based on Seq + Str features represents a hierarchal segmentation of the data. The original segment is the entire dataset, also known as the root node of the tree, and it is first portioned into two or more segments by applying a series of simple rules. Each rule assigns an observation on a segment based on the value of an explanatory variable for that observation. For example, the decision tree model first selects the RSA_4 variable (i.e., the RSA of the 4^th^ position in the octapeptide) as the first rule to distinguish cleavage sites and non-cleavage sites. If the RSA_4 value of an octamer is greater than or equal to 0.4055, we follow the right subtree; otherwise, the rules in the left subtree are applied. In a similar fashion, each resulting segment is further portioned into sub-segments, and each sub-segment is further portioned into more sub-segments. From the right subtree of the previous example, the second rule selected by decision tree model to identify cleavage sites is MT (i.e., the DipC of methionine and threonine in the octapeptide). When MT is greater than or equal to 7.145, the octapeptide is predicted as cleavage site in the right subtree; otherwise, rules from the left subtree are applied for further partition. This process continues until no more portioning is possible. This process of segmenting is called recursive portioning, and it results in a hierarchy of segments within segments. The decision trees for the other three datasets are illustrated in Figure S5–S7 of the supplementary material [Additional file 6]. In Table 7, variable importance of the decision tree model in Fig. 2 is ranked by reduction of Gini index for the training set. The top ranked variables RSA_4 and RSA_5 variables corresponded well with the findings that solvent accessibility served as a discriminative feature to predict cleavage sites [16]. Moreover, the selected RSA_4 and RSA_5 variables suggested that the centered position 4 and position 5 in the octapeptide play a crucial role for identification of cleavage sites, and these two positions have also been illustrated important in a traditional classification of HIV-1 protease substrates [5]. This lends support on our assumption that our method can identify important biological features to identify cleavage sites. In addition, combinations of hydrophobic and polar amino acid dipeptides (i.e., MT, VH, AE, and FL), which can form hydrogen bonds with others, are selected as important features preferred for cleavage sites [32].

**Table 7:** Interpretable biological features selected by decision tree model based on Seq + Str features for the 746 dataset

Rank	Variable	Description	Importance
1	RSA_4	RSA at the 4^th^ position of an octamer	1.0000
2	MT	DipC of methionine & threonine	0.7855
3	C	AAC of cysteine	0.6060
4	RSA_5	RSA at the 5^th^ position of an octamer	0.5238
5	VH	DipC of valine & histidine	0.4059
6	AE	DipC of alanine & glutamic acid	0.3769
7	FL	DipC of phenylalanine & leucine	0.3268

## Conclusions

To predict protease cleavage site, the understanding of HIV-1 protease specificity becomes imperative. In this study, we demonstrated that the combination of various sequence, structure, and physicochemical features can play a vital role in the identification of HIV-1 protease cleavage sites and understanding of the specificity of the substrates. We incorporated three machine learning algorithms to compare the predictive performance of protease cleavage sites. Experiment results suggested that the hybrid biological features performed better than the single feature types. In addition, the results also lend support on our assumption that incorporation of various biological features can compensate each other and achieve more accurate performance. Moreover, through this study, we can identify an effective set of feature combinations that help identify the highly favorable sites where cleavage events take place. The source codes and datasets are freely available for download as standalone software from the link provided here (https://drive.google.com/open?id=0B-_hwmxkV77wNlY0cUxoQmcyOWc).

## Additional files

Additional file 1: The 746 dataset. (PDF 370 kb)
Additional file 2: The 1625 dataset. (PDF 599 kb)
Additional file 3: The Schilling dataset. (PDF 401 kb)
Additional file 4: The Impens dataset. (PDF 1093 kb)
Additional file 5: The ROC plots for the four benchmark datasets. (PDF 675 kb)
Additional file 6: Decision trees of the 1625, Schilling, and Impens datasets. (PDF 464 kb)
