# Supplementary material for: Prediction of HIV-1 protease cleavage site using a combination of sequence, structural, and physicochemical features
Source: BMC Bioinformatics. 2016 Dec 23;17(Suppl 17):478. doi: 10.1186/s12859-016-1337-6 (PMC5259813; doi:10.1186/s12859-016-1337-6)
Supplement: Additional file 1: — The 746 dataset. (PDF 370 kb) [file 12859_2016_1337_MOESM1_ESM.pdf]

Additional File 1: The 746 dataset

AAAKFERQ,-1	ARVLAEAK,1	CELAAAMK,-1	FVHESLAD,-1	GVYQLSAL,-1	KTKVKVVQ,-1
AAAMKRHG,-1	ARVLAEAM,1	CKGTDVQA,-1	FVNNGLVK,-1	GWILAEHG,1	KTKVLKVQ,-1
AAAMSSAI,-1	ARVLAEAP,1	CKNGQTNC,-1	FVVNGLVK,-1	GWILGEHG,1	KTTQANKH,-1
AAKFERQH,-1	ARVLAEAR,1	CKPVNTFV,-1	GALTNAVL,-1	GWVMTEAL,1	KVFGRCEL,-1
AAKFESNF,-1	ARVLAEDM,-1	CNDGRTPG,-1	GAMVNQAL,-1	GYSLGNWV,-1	KVKVVQPK,-1
AAMKRHGL,-1	ARVLAEEM,1	CNIPCSAL,-1	GAVSLAMT,1	HAVSLAMT,1	KVLKVQPK,-1
AASSSNYC,-1	ARVLAEFM,1	CNQMMKSR,-1	GAVWLAMT,1	HESLADVQ,-1	KYPNCAYK,-1
AAVLAEAM,1	ARVLAEGM,1	CRETGSSK,-1	GDALLERN,1	HGLDNYRG,-1	LAAAMKRH,-1
ACEGNPYV,-1	ARVLAERM,1	CSALLSSD,-1	GDAYFSVP,1	HIIVACEG,-1	LADVQAVC,-1
ACKNGQTN,-1	ARVLAESM,1	CSQKNVAC,-1	GEMFFPVL,1	HLVEALYL,1	LAVSLAMT,1
ADVQAVCS,-1	ARVLAGAM,-1	CYQSYSTM,-1	GFAMAEAL,1	HMDSSTSA,-1	LCNIPCSA,-1
AEAMSQVT,1	ARVLALAM,1	DAINTEFK,1	GFIGVSYL,1	HYGFPTYG,1	LDNYRGYS,-1
AECFRIFD,1	ARVLANAM,-1	DCRETGSS,-1	GGNYPVQH,1	IIVACEGN,-1	LECLLSIP,1
AEELAEIF,1	ARVLAQAM,-1	DDLFFEAD,1	GGRINVAL,-1	ILQINSRW,-1	LEVNIVTD,1
AETFYTDG,1	ARVLAVAM,1	DGNGMNAW,-1	GGVYATRS,1	INSRWWCN,-1	LGNWVCAA,-1
AETFYVDG,1	ARVLFAAL,1	DGRTPGSR,-1	GILQINSR,-1	INVALVPK,-1	LKVLVVQP,1
AETFYVDK,1	ARVLFDAL,1	DGSTDYGI,-1	GKVLVVQP,1	IPCSALLS,-1	LLSSDITA,-1
AFVLAEAM,1	ARVLFEAL,1	DITASVNC,-1	GLAAPQFS,1	ITASVNCA,-1	LPVNGEFS,1
AGVLAEAM,1	ARVLFEAM,1	DKVLVVQP,1	GLDNYRGY,-1	ITDCRETG,-1	LQINSRWW,-1
AKFERQHM,-1	ARVLFIAL,1	DLVLLSAE,1	GLNMPALV,1	IVACEGNP,-1	LQNYPIVQ,1
AKFESNFN,-1	ARVLFNAL,1	DNYRGYSL,-1	GLTMVQEL,1	IVSDGNGM,-1	LQVLTLNI,-1
AKKIVSDG,-1	ARVLFQAL,1	DQILIEIC,1	GLTMVTQL,-1	KDIFPVTE,1	LSSDITAS,-1
AKVLAEAM,1	ARVLFTAL,1	DQNYPIVQ,1	GLVAFANL,1	KDRCKPVN,-1	LTKDRCKP,-1
AKVLVVQP,1	ARVLFVAL,1	DRCKPVNT,-1	GLVLQEGL,1	KELYPLTS,1	LTMVTQLV,-1
ALLSSDIT,-1	ARVLGEAM,1	DSADAEED,1	GMNAWVAW,-1	KETAAAKF,-1	LTNAVLVP,-1
ALTNAVLV,-1	ARVLIEAM,1	DSSTSAAS,-1	GNGMNAWV,-1	KFERQHMD,-1	LWMGYELH,1
AMKRHGLD,-1	ARVLLEAM,1	DTVLEEMS,1	GNPYVPVH,-1	KFESNFNT,-1	MDSSTSAA,-1
AMVNQALV,-1	ARVLMEAM,1	DVQAVCSQ,-1	GNWVCAAK,-1	KGSGALTN,-1	MKRHGLDN,-1
ANKHIIVA,-1	ARVLNEAM,1	DVQAWIRG,-1	GQANFLGK,1	KGSGAMVN,-1	MKSRNLTK,-1
AQNYPIVL,1	ARVLVEAM,1	DYGILQIN,-1	GQDFPMYL,1	KGSGGRIN,-1	MMKSRNLT,-1
AQNYPIVQ,1	ARVMAEAM,1	EEIMLAYQ,1	GQNYPIVQ,1	KGSGLTMV,-1	MNAWVAWR,-1
AQTFYVNL,1	ARVNAEAM,1	EGNPYVPV,-1	GQTNCYQS,-1	KGSGTWMV,-1	MQNYPIVQ,1
AQVLAEAM,1	ARVVAEAM,-1	EKVYLAWV,1	GQVNFLGK,1	KGSGVYQL,-1	MRVLAEAM,1
ARALAEAM,1	ARVYAEAM,1	ELAAAMKR,-1	GQVNYEEF,1	KGTDVQAW,-1	MSITDCRE,-1
ARGLAEAM,1	ARVYPEAL,1	ELEFPEGG,1	GRCELAAA,-1	KHIIVACE,-1	MVHSLVPK,-1
ARILAEAM,1	ASILPVIP,-1	ELELAENR,1	GRINVALV,-1	KIVSDGNG,-1	MVNQALVP,-1
ARLMAEAL,1	ASSSNYCN,-1	ELILPVKR,1	GRTPGSRN,-1	KKIVSDGN,-1	MVTQLVPK,-1
ARNLAEAM,-1	ASVNCAKK,-1	ERQHMDSS,-1	GSGALTNA,-1	KKLVVQPK,-1	NAVSLAMT,1
ARNLFEAL,1	ATAMMATA,1	ERVLAEAM,1	GSGAMVNQ,-1	KLVLAQLS,1	NAWVAWRN,-1
ARNLFIAL,1	ATIMMITA,1	ESLADVQA,-1	GSGGRINV,-1	KMMLLAKA,1	NCAKKIVS,-1
ARNLFQAL,1	ATIMMQRG,1	ESNFNTQA,-1	GSGLTMVT,-1	KNGQTNCY,-1	NCAYKTTQ,-1
ARNLFVAL,1	ATIYYITA,1	ETAAAKFE,-1	GSGTWMVH,-1	KNVACKNG,-1	NCYQSYST,-1
ARNYPEAL,1	ATNRNTDG,-1	ETGSSKYP,-1	GSGVYQLS,-1	KPVNTFVH,-1	NDGRTPGS,-1
ARNYPIVL,1	ATVLAEAM,1	ETTALVCD,1	GSHLVEAL,1	KQNYPIVQ,1	NFNTQATN,-1
ARRLAEAM,1	ATVLTVAL,1	FAVSLAMT,1	GSRNLCNI,-1	KQTFPIQQ,-1	NGMNAWVA,-1
ARSLAEAM,-1	AVCSQKNV,-1	FERQHMDS,-1	GSSKYPNC,-1	KRHGLDNY,-1	NGQTNCYQ,-1
ARVAAEAM,1	AWIRGCRL,-1	FESNFNTQ,-1	GSTDYGIL,-1	KSGVFSVN,-1	NIPCSALL,-1
ARVFAEAM,1	AWRNRCKG,-1	FGRCELAA,-1	GTDVQAWI,-1	KSGVFVNN,-1	NKHIIVAC,-1
ARVIAEAM,1	AWVAWRNR,-1	FHVNGLVK,-1	GTWMVHSL,-1	KSGVNVVN,-1	NLCNIPCS,-1
ARVLAAAM,-1	AYKTTQAN,-1	FNTQATNR,-1	GVALSALV,-1	KSGVQVVN,-1	NLTKDRCK,-1
ARVLADAM,-1	CAAKFESN,-1	FQAYPLRE,1	GVFSVNGL,-1	KSRNLTKD,-1	NPYVPVHF,-1
ARVLAEAA,1	CAKKIVSD,-1	FQVNGLVK,-1	GVFVNNGL,-1	KSRVNVVN,-1	NQMMKSRN,-1
ARVLAEAE,1	CAYKTTQA,-1	FRSGVETT,1	GVNVVNGK,-1	KSRVQVVN,-1	NQNYPIVQ,1
ARVLAEAG,1	CEGNPYVP,-1	FSVNGLVK,-1	GVQVVNGK,-1	KTKKLVVQ,-1	NRCKGTDV,-1
NRNTDGST,-1	PGLSLAMT,1	RCKPVNTF,-1	SFNYPQIT,1	SKNYPIVQ,1	SSKYPNCA,-1
NSRWWCND,-1	PGNFFQSR,1	REAFRVFD,1	SFTFPQIT,1	SKVLVVQP,1	SSLYPALT,1
NSVMIALV,1	PGNFLQSR,1	RETGSSKY,-1	SFTYYTDS,1	SKYPNCAY,-1	SSNYCNQM,-1
NTDGSTDY,-1	PGNFPQSR,1	RGYSLGNW,-1	SFVFVVNG,1	SLADVQAV,-1	SSSNYCNQ,-1
NTFVHESL,-1	PGNFVQSR,1	RHGLDNYR,-1	SFYFPQIT,1	SLGNWVCA,-1	SSTSAASS,-1
NTQATNRN,-1	PGNLLQSR,1	RINVALVP,-1	SGAFMTRG,1	SLNLPVAK,1	STDYGILQ,-1

NVACKNGQ,-1	PGNYLQSR,1	RKILFLDG,1	SGALTNAV,-1	SLNLRETN,1	STLLIENS,-1
NWVCAAKF,-1	PGSRNLCN,-1	RKVLFLDG,1	SGAMVNQA,-1	SLTYYTDS,1	STMSITDC,-1
NYCNQMMK,-1	PGVSLAMT,1	RNLCNIPC,-1	SGAYLIQG,1	SNFNTQAT,-1	STSAASSS,-1
NYRGYSLG,-1	PGVWLAMT,1	RNLTKDRC,-1	SGGRINVA,-1	SNNYPIVQ,1	SVNCAKKI,-1
PAASFAMT,1	PHVSLAMT,1	RNRCKGTD,-1	SGIFLETS,1	SNYCNQMM,-1	SYNFPQIT,1
PAASLAMT,1	PHVWLAMT,1	RNTDGSTD,-1	SGIFVVNG,1	SQAFPLRA,1	SYSTMSIT,-1
PAAWLAMT,1	PIVGAETF,1	RQANFLGK,1	SGIMFESN,1	SQAYPIVQ,1	TAAAKFER,-1
PAGSFAMT,1	PKVLVVQP,1	RQHMDSST,-1	SGIMFQSA,1	SQCYPIVQ,1	TASVNCAK,-1
PAGSLAMT,1	PLIMAVVN,1	RQNYPIAL,1	SGINFESG,1	SQIYPIVQ,1	TAVLVVQP,1
PAGWLAMT,1	PMVGVLDA,1	RQNYPIVQ,1	SGIYLVEN,1	SQKNVACK,-1	TDCRETGS,-1
PAHSFAMT,1	PNCAYKTT,-1	RQVLFLEK,1	SGIYTVQS,1	SQKYPIVQ,-1	TDGSTDYG,-1
PAHSLAMT,1	PNVSLAMT,1	RQVNFLGK,1	SGIYYSVS,1	SQLYPIVQ,1	TDVLVVQP,1
PAILPIIS,1	PPVSLAMT,1	RRANFLGK,1	SGLFTEYG,1	SQNFPIVQ,1	TDVQAWIR,-1
PAILVHTP,-1	PQNFLQSR,1	RRVLAEAM,1	SGLTMVTQ,-1	SQNMPIVQ,1	TDYGILQI,-1
PALSFAMT,1	PQNYPIVQ,1	RRVNFLGK,1	SGLYYVTE,1	SQNPPIVQ,-1	TFNFPQIT,1
PALSLAMT,1	PQVLPVMH,1	RTPGSRNL,-1	SGMWFEAP,1	SQNSPIVQ,-1	TFTFPVVF,1
PALWLAMT,1	PRASLAMT,1	RVNVVNGK,-1	SGNFAAFS,1	SQNYAIVQ,1	TFVHESLA,-1
PASSLAMT,1	PRGSLAMT,1	RVQVVNGK,-1	SGNFVVNG,1	SQNYDIVQ,-1	TFVLVVQP,1
PASWLAMT,1	PRHSLAMT,1	RWWCNDGR,-1	SGNMLVYS,1	SQNYKIVQ,-1	TGSSKYPN,-1
PAVALAMT,1	PRLSLAMT,1	SAASSSNY,-1	SGNMVMFG,1	SQNYLIVQ,1	TGVLVVQP,1
PAVELAMT,1	PRNFLQSR,1	SALLSSDI,-1	SGNYFVET,1	SQNYPAVQ,1	TKALVVQP,1
PAVGLAMT,1	PRNFPVAQ,1	SCNFPQIT,1	SGNYFVQG,1	SQNYPIDQ,1	TKDLVVQP,1
PAVHLAMT,1	PRVALAMT,1	SDAYYADS,1	SGNYLVTS,1	SQNYPIEQ,1	TKDRCKPV,-1
PAVIFAMT,1	PRVGLAMT,1	SDAYYTDS,1	SGSYVEYQ,1	SQNYPIFQ,1	TKFLVVQP,1
PAVILAMT,1	PRVLAEAM,1	SDCYYCDS,1	SGTFQVQL,1	SQNYPIIQ,1	TKILVVQP,1
PAVLAAMT,1	PRVLLAMT,1	SDCYYTDS,1	SGTWMVHS,-1	SQNYPILQ,1	TKKLVVQP,-1
PAVLFAMT,1	PRVSLAMT,1	SDEYYEDS,1	SGTYYTDS,1	SQNYPIVE,1	TKLLVVQP,1
PAVLGAMT,1	PRVWLAMT,1	SDEYYTDS,1	SGTYYTGS,1	SQNYPIVL,1	TKNLVVQP,1
PAVLLAMT,1	PSVSLAMT,1	SDGNGMNA,-1	SGVFAVTQ,1	SQNYPIVP,1	TKVAVVQP,1
PAVRLAMT,1	PSVWLAMT,1	SDGYYTDS,1	SGVFHVNG,1	SQNYPIVQ,1	TKVFVVQP,1
PAVSAAMT,1	PTLLTEAP,1	SDITASVN,-1	SGVFQVNG,1	SQNYPKVQ,-1	TKVGVVQP,1
PAVSEAMT,1	PTVSLAMT,1	SDIYYTDS,1	SGVFSVNG,-1	SQNYPLVQ,1	TKVKVVQP,-1
PAVSFAMT,1	PTVWLAMT,1	SDLYYTDS,1	SGVFTEER,1	SQNYPNVQ,1	TKVLFVQP,1
PAVSGAMT,1	PVHFDASV,-1	SDNYPIVQ,1	SGVFVEMP,1	SQNYPTVQ,1	TKVLIVQP,1
PAVSLALT,1	PVILPIQA,1	SDTYYADS,1	SGVFVENG,1	SQNYPVVQ,1	TKVLKVQP,-1
PAVSLAMT,1	PVNTFVHE,-1	SDTYYCDS,1	SGVFVETS,1	SQNYTIVQ,1	TKVLLVQP,1
PAVSLANT,1	PYVGSGLY,-1	SDTYYEDS,1	SGVFVNNG,-1	SQNYYIDQ,1	TKVLPVQP,1
PAVSLAYT,1	PYVPVHFD,-1	SDTYYGDS,1	SGVFVQNG,1	SQNYYIVQ,1	TKVLSVQP,1
PAVSLGMT,1	QAIYLALQ,1	SDTYYIDS,1	SGVFVVNG,1	SQNYYNQS,1	TKVLVVQP,1
PAVSLHMT,1	QANFLGKI,-1	SDTYYLDS,1	SGVFVVQG,1	SQNYYTDQ,1	TKVMVVQP,1
PAVSLLMT,1	QANKHIIV,-1	SDTYYTDQ,1	SGVFVVTG,1	SQNYYTVQ,1	TKVYVVQP,1
PAVSLSMT,1	QATNRNTD,-1	SDTYYTDS,1	SGVFYSRE,1	SQQYPIVQ,-1	TLNFPISP,1
PAVSQAMT,1	QAVCSQKN,-1	SDTYYTFS,1	SGVFYTLV,-1	SQTYPIVQ,1	TLNFPQIT,1
PAVSRAMT,1	QAWIRGCR,-1	SDTYYTGS,1	SGVHFISR,1	SQTYYIDQ,1	TLVLVVQP,1
PAVSVAMT,1	QGNFLQSR,1	SDTYYTLS,1	SGVHVEYT,1	SQTYYIVQ,1	TMSITDCR,-1
PAVVLAMT,1	QHMDSSTS,-1	SDTYYTQS,1	SGVLFVSS,1	SQTYYTDQ,1	TMVTQLVP,-1
PAVWAAMT,1	QINSRWWC,-1	SEEYPIMI,1	SGVMFQTD,1	SQTYYTDS,1	TNAVLVPK,-1
PAVWFAMT,1	QITLPKRP,1	SFIGMESA,1	SGVMPTMS,1	SQTYYTQS,1	TNCYQSYS,-1
PAVWGAMT,1	QITLWQRP,1	SFNFGQIT,1	SGVNVVNG,-1	SQTYYTVQ,1	TNRNTDGS,-1
PAVWLAMT,1	QKNVACKN,-1	SFNFLQIT,1	SGVQVVNG,-1	SQVYPIVQ,1	TPGSRNLC,-1
PAVWVAMT,1	QLSALVPK,-1	SFNFPLIT,1	SGVYHVST,1	SRNLCNIP,-1	TQANKHII,-1
PAWSLAMT,1	QMIFEEHG,1	SFNFPPIT,1	SGVYLATD,1	SRNLTKDR,-1	TQATNRNT,-1
PCSALLSS,-1	QMMKSRNL,-1	SFNFPQDT,1	SGVYQLSA,-1	SRSLYASS,1	TQDFWEVQ,1
PDVSLAMT,1	QSYSTMSI,-1	SFNFPQII,1	SITDCRET,-1	SRVNVVNG,-1	TQIMFETF,1
PFAAAQQR,1	QTNCYQSY,-1	SFNFPQIT,1	SKAFLADT,1	SRVQVVNG,-1	TQNYPIVQ,1
PFIFEEEP,1	RCELAAAM,-1	SFNFPQVT,1	SKDLIAEI,1	SRWWCNDG,-1	TSAASSSN,-1
PFVSLAMT,1	RCKGTDVQ,-1	SFNSPQIT,1	SKLLATVV,-1	SSDITASV,-1	TSCYCHGT,1
TSLLTLDD,-1					
TSVLVVQP,1					
TTQANKHI,-1					
TVVLVVQP,1					
TWMVHSLV,-1					
VACEGNPY,-1					

VACKNGQT,-1
VAWRNRCK,-1
VCAAKFES,-1
VCSQKNVA,-1
VEICTEME,1
VEVAEEEE,1
VFGRCELA,-1
VFSVNGLV,-1
VFVNNGLV,-1
VHESLADV,-1
VKVLVVQP,1
VNCAKKIV,-1
VNQALVPK,-1
VNTFVHES,-1
VPVHFDAS,-1
VQAVCSQK,-1
VQAWIRGC,-1
VSDGNGMN,-1
VVAMPVVI,-1
VYQLSALV,-1
WCNDGRTP,-1
WMVHSLVP,-1
WRNRCKGT,-1
WVAWRNRC,-1
WVCAAKFE,-1
WWCNDGRT,-1
YCNQMMKS,-1
YEEFVQMM,1
YFNFPQIT,1
YGILQINS,-1
YKTTQANK,-1
YPNCAYKT,-1
YQLSALVP,-1
YQSYSTMS,-1
YRGYSLGN,-1
YSLGNWVC,-1
YSTMSITD,-1
YVPVHFDA,-1
