# Supplementary material for: Prediction of HIV-1 protease cleavage site using a combination of sequence, structural, and physicochemical features
Source: BMC Bioinformatics. 2016 Dec 23;17(Suppl 17):478. doi: 10.1186/s12859-016-1337-6 (PMC5259813; doi:10.1186/s12859-016-1337-6)
Supplement: Additional file 2: — The 1625 dataset. (PDF 599 kb) [file 12859_2016_1337_MOESM2_ESM.pdf]

Additional File 2: The 1625 dataset

SLNLRETN,1	AKFERQHM,-1	YCNQMMKS,-1	AMKRHGLD,-1	AAKFESNF,-1	RRVLAEAM,1
AECFRIFD,1	QTNCYQSY,-1	VQAWIRGC,-1	QMMKSRNL,-1	KVFGRCEL,-1	ARVLAEEM,1
HLVEALYL,1	SSSNYCNQ,-1	VNTFVHES,-1	PVNTFVHE,-1	YGILQINS,-1	ARVLAEGM,1
TQIMFETF,1	PCSALLSS,-1	TMSITDCR,-1	KSRNLTKD,-1	ITASVNCA,-1	HAVSLAMT,1
AEELAEIF,1	VNCAKKIV,-1	IPCSALLS,-1	NCYQSYST,-1	AWRNRCKG,-1	PASSLAMT,1
PFIFEEEP,1	GSRNLCNI,-1	NVACKNGQ,-1	NKHIIVAC,-1	LSSDITAS,-1	PHVSLAMT,1
PIVGAETF,1	NGMNAWVA,-1	SDGNGMNA,-1	AASSSNYC,-1	AWIRGCRL,-1	PAVWLAMT,1
ETTALVCD,1	HESLADVQ,-1	DVQAWIRG,-1	STMSITDC,-1	ASVNCAKK,-1	PDVSLAMT,1
GGVYATRS,1	DGNGMNAW,-1	KTTQANKH,-1	SSKYPNCA,-1	SITDCRET,-1	PRVSLAMT,1
DAINTEFK,1	TDCRETGS,-1	QHMDSSTS,-1	NQMMKSRN,-1	KRHGLDNY,-1	PAVALAMT,1
DDLFFEAD,1	GTDVQAWI,-1	GSTDYGIL,-1	GMNAWVAW,-1	RNRCKGTD,-1	PAVSLANT,1
SFIGMESA,1	TQANKHII,-1	TAAAKFER,-1	ATNRNTDG,-1	KNGQTNCY,-1	PPVSLAMT,1
PTLLTEAP,1	NDGRTPGS,-1	SALLSSDI,-1	TASVNCAK,-1	DITASVNC,-1	PALSLAMT,1
DQILIEIC,1	FESNFNTQ,-1	MNAWVAWR,-1	AAAKFERQ,-1	YSLGNWVC,-1	FAVSLAMT,1
AQTFYVNL,1	ALLSSDIT,-1	TSAASSSN,-1	FNTQATNR,-1	NFNTQATN,-1	PAVGLAMT,1
QITLWQRP,1	CAKKIVSD,-1	KYPNCAYK,-1	WRNRCKGT,-1	SSTSAASS,-1	GAVSLAMT,1
GSHLVEAL,1	STDYGILQ,-1	RWWCNDGR,-1	AKFESNFN,-1	SVNCAKKI,-1	PAVSEAMT,1
REAFRVFD,1	TNCYQSYS,-1	NTFVHESL,-1	YQSYSTMS,-1	RTPGSRNL,-1	PAVSFAMT,1
YEEFVQMM,1	PYVPVHFD,-1	VCAAKFES,-1	ESNFNTQA,-1	KFERQHMD,-1	NAVSLAMT,1
GQVNYEEF,1	SDITASVN,-1	FVHESLAD,-1	CSALLSSD,-1	MSITDCRE,-1	PNVSLAMT,1
LPVNGEFS,1	CEGNPYVP,-1	QSYSTMSI,-1	QINSRWWC,-1	MKSRNLTK,-1	PAVSGAMT,1
ELEFPEGG,1	RNTDGSTD,-1	NLTKDRCK,-1	NYCNQMMK,-1	NRNTDGST,-1	PAVSLALT,1
VEVAEEEE,1	YSTMSITD,-1	CKNGQTNC,-1	ESLADVQA,-1	RCELAAAM,-1	PAVELAMT,1
DTVLEEMS,1	KFESNFNT,-1	NAWVAWRN,-1	ERQHMDSS,-1	TDYGILQI,-1	PAVHLAMT,1
GDALLERN,1	HMDSSTSA,-1	NYRGYSLG,-1	CNQMMKSR,-1	ARVLMEAM,1	PAWSLAMT,1
AAKFERQH,-1	KHIIVACE,-1	SRNLCNIP,-1	AWVAWRNR,-1	ARVLIEAM,1	PAVSLGMT,1
MDSSTSAA,-1	DGSTDYGI,-1	QAWIRGCR,-1	WWCNDGRT,-1	ARVLFEAM,1	PAVSLSMT,1
SSNYCNQM,-1	FGRCELAA,-1	MMKSRNLT,-1	VAWRNRCK,-1	ARVLLEAM,1	PGVSLAMT,1
TPGSRNLC,-1	NRCKGTDV,-1	TNRNTDGS,-1	KKIVSDGN,-1	ARVLVEAM,1	PAGSLAMT,1
GSSKYPNC,-1	ETAAAKFE,-1	FERQHMDS,-1	VHESLADV,-1	AFVLAEAM,1	PAVSLHMT,1
ETGSSKYP,-1	RQHMDSST,-1	RNLCNIPC,-1	AYKTTQAN,-1	ARVLNEAM,1	PAVSRAMT,1
LTKDRCKP,-1	YVPVHFDA,-1	VCSQKNVA,-1	ELAAAMKR,-1	ARVFAEAM,1	PAVRLAMT,1
RCKGTDVQ,-1	KIVSDGNG,-1	AAAMKRHG,-1	GLDNYRGY,-1	ARVLAEAE,1	PAVSLLMT,1
SLGNWVCA,-1	CKPVNTFV,-1	VQAVCSQK,-1	GRCELAAA,-1	PRVLAEAM,1	LAVSLAMT,1
PGSRNLCN,-1	SRNLTKDR,-1	KPVNTFVH,-1	SSDITASV,-1	ARVLAEAA,1	PAVSLAYT,1
VFGRCELA,-1	NTDGSTDY,-1	LGNWVCAA,-1	SNYCNQMM,-1	AAVLAEAM,1	PAVSQAMT,1
NCAKKIVS,-1	GNWVCAAK,-1	RHGLDNYR,-1	NLCNIPCS,-1	ARVAAEAM,1	PAVLLAMT,1
NSRWWCND,-1	TTQANKHI,-1	RGYSLGNW,-1	SLADVQAV,-1	ARVLAEAR,1	GAVWLAMT,1
SQKNVACK,-1	QANKHIIV,-1	KNVACKNG,-1	STSAASSS,-1	ARVLAEAK,1	PAVWVAMT,1
CAYKTTQA,-1	WVCAAKFE,-1	NPYVPVHF,-1	GQTNCYQS,-1	ARVLAVAM,1	PAHSFAMT,1
SRWWCNDG,-1	NGQTNCYQ,-1	GYSLGNWV,-1	IIVACEGN,-1	ARVIAEAM,1	PAVWFAMT,1
ASSSNYCN,-1	LAAAMKRH,-1	KETAAAKF,-1	AAMKRHGL,-1	ARVNAEAM,1	PAHSLAMT,1
CYQSYSTM,-1	PNCAYKTT,-1	TGSSKYPN,-1	TQATNRNT,-1	AKVLAEAM,1	PAVSVAMT,1
AVCSQKNV,-1	HGLDNYRG,-1	DRCKPVNT,-1	CELAAAMK,-1	ARVYAEAM,1	PAGWLAMT,1
GNGMNAWV,-1	TFVHESLA,-1	ACKNGQTN,-1	CNIPCSAL,-1	ARGLAEAM,1	PAVWGAMT,1
AKKIVSDG,-1	SNFNTQAT,-1	LLSSDITA,-1	YRGYSLGN,-1	ATVLAEAM,1	PAVLAAMT,1
RNLTKDRC,-1	IVSDGNGM,-1	DYGILQIN,-1	NCAYKTTQ,-1	ARVLAEAG,1	PAVSAAMT,1
SYSTMSIT,-1	TDVQAWIR,-1	KGTDVQAW,-1	ANKHIIVA,-1	AGVLAEAM,1	PAASLAMT,1
CAAKFESN,-1	CKGTDVQA,-1	DGRTPGSR,-1	ILQINSRW,-1	ARVLAEFM,1	PRGSLAMT,1
CSQKNVAC,-1	TKDRCKPV,-1	IVACEGNP,-1	WCNDGRTP,-1	MRVLAEAM,1	PSVWLAMT,1
QATNRNTD,-1	DVQAVCSQ,-1	ACEGNPYV,-1	WVAWRNRC,-1	ARRLAEAM,1	PRASLAMT,1
INSRWWCN,-1	RETGSSKY,-1	DNYRGYSL,-1	ADVQAVCS,-1	ARVLAEAP,1	PAASFAMT,1
YKTTQANK,-1	ITDCRETG,-1	HIIVACEG,-1	TDGSTDYG,-1	ARALAEAM,1	PAVILAMT,1
CRETGSSK,-1	QAVCSQKN,-1	LADVQAVC,-1	VACKNGQT,-1	ARVMAEAM,1	PGLSLAMT,1
VPVHFDAS,-1	LDNYRGYS,-1	NTQATNRN,-1	NIPCSALL,-1	ARVLAERM,1	PAGSFAMT,1
EGNPYVPV,-1	GRTPGSRN,-1	QKNVACKN,-1	KDRCKPVN,-1	ERVLAEAM,1	PAVWAAMT,1
VACEGNPY,-1	CNDGRTPG,-1	RCKPVNTF,-1	GILQINSR,-1	ARILAEAM,1	PRVALAMT,1
GNPYVPVH,-1	SAASSSNY,-1	DCRETGSS,-1	DSSTSAAS,-1	ARVLALAM,1	PALSFAMT,1
MKRHGLDN,-1	SKYPNCAY,-1	VSDGNGMN,-1	PVHFDASV,-1	ARVLGEAM,1	PRHSLAMT,1
NWVCAAKF,-1	LCNIPCSA,-1	LQINSRWW,-1	YPNCAYKT,-1	ARVLAESM,1	PAVLFAMT,1

PRVGLAMT,1	SQNFPIVQ,1	RQVLFLEK,1	GKVLVVQP,1	FVNNGLVK,-1	SKRTFLIK,-1
PRLSLAMT,1	SQNYPILQ,1	ARLMAEAL,1	TKLLVVQP,1	MVTQLVPK,-1	SYRRMFGG,-1
PTVSLAMT,1	SQLYPIVQ,1	TLNFPISP,1	TKVYVVQP,1	SGVQVVNG,-1	DTIGRLQD,-1
PAAWLAMT,1	SQNMPIVQ,1	PFAAAQQR,1	TDVLVVQP,1	SRVNVVNG,-1	LKKLHEEE,-1
PRVWLAMT,1	SQNVPIVQ,-1	ATIMMQRG,1	TFVLVVQP,1	LTNAVLVP,-1	THSKRTFL,-1
PFVSLAMT,1	SQNIPIVQ,-1	RKILFLDG,1	TKDLVVQP,1	TNAVLVPK,-1	HEEEIQEL,-1
PTVWLAMT,1	SQNGPIVQ,-1	PQNFLQSR,1	TKNLVVQP,1	KSGVNVVN,-1	KNLQEAEE,-1
PAVVLAMT,1	SQNDPIVQ,-1	SFNFPQIT,1	TAVLVVQP,1	SGVFVNNG,-1	RFLEQQNK,-1
PAVIFAMT,1	SQNYPKVQ,-1	PRNFPVAQ,1	TLVLVVQP,1	KSGVFVNN,-1	REEAENTL,-1
PAVLGAMT,1	SQNKPIVQ,-1	FRSGVETT,-1	TKVLLVQP,1	GSGALTNA,-1	GVRLLQDS,-1
PSVSLAMT,1	SQNSPIVQ,-1	AEAMSQVT,-1	TSVLVVQP,1	SGTWMVHS,-1	RDNLAEDI,-1
PGVWLAMT,1	SQNPPIVQ,-1	RQAGFLGL,-1	SQVYPIVQ,1	GVQVVNGK,-1	LGDLYEEE,-1
PRVLLAMT,1	SQKYPIVQ,-1	PAILPIIS,1	TKVAVVQP,1	KSRVQVVN,-1	EAANRNND,-1
PALWLAMT,1	SDGYYTDS,1	SSLYPALT,1	PKVLVVQP,1	SGLTMVTQ,-1	IDKVRFLE,-1
PAHWLAMT,1	ATIYYITA,1	GGNYPVQH,1	TKALVVQP,1	GSGLTMVT,-1	ESTEYRRQ,-1
PHVWLAMT,1	SDTYYLDS,1	TFTFPVVF,1	SKVLVVQP,1	AMVNQALV,-1	SVSSSSYR,-1
KQNYPIVQ,1	SDNYPIVQ,1	KDIFPVTE,1	LKVLVVQP,1	GAMVNQAL,-1	IGRLQDEI,-1
TQNYPIVQ,1	SQTYYTQS,1	PMVGVLDA,1	AKVLVVQP,1	LTMVTQLV,-1	SLQEEIAF,-1
DQNYPIVQ,1	ATAMMATA,1	ATVLTVAL,1	TGVLVVQP,1	GTWMVHSL,-1	LSEAANRN,-1
NQNYPIVQ,1	SLTYYTDS,1	KLVLAQLS,1	TKVGVVQP,1	KGSGTWMV,-1	LNDRFANY,-1
GQNYPIVQ,1	SDTYYADS,1	SQAFPLRA,1	TKVLKVQP,-1	RVQVVNGK,-1	RETNLDSL,-1
LQNYPIVQ,1	SQTYYTDS,1	LECLLSIP,1	TKVKVVQP,-1	SGVNVVNG,-1	LLQDSVDF,-1
RQNYPIVQ,1	SDAYYTDS,1	EEIMLAYQ,1	TKKLVVQP,-1	GSGTWMVH,-1	RLGDLYEE,-1
PQNYPIVQ,1	SQNYYNQS,1	ELILPVKR,1	SQNYAIVQ,1	NSVMIALV,1	RPSSSRSY,-1
AQNYPIVQ,1	SFTYYTDS,1	PLIMAVVN,1	SQNYKIVQ,-1	SGMWFEAP,1	LVDTHSKR,-1
MQNYPIVQ,1	SDTYYTDQ,1	TSCYHCGT,1	SQNYDIVQ,-1	GWVMTEAL,1	QLTNDKAR,-1
SQNYPIVQ,1	SQNYYTDQ,1	DLVLLSAE,1	SGVFVVTG,1	GLTMVQEL,1	DALKGTNE,-1
ATIMMQRE,1	SDAYYADS,1	SKAFLADT,1	SGNFVVNG,1	SGNFAAFS,1	KILLAELE,-1
AAAMSSAI,1	SDEYYTDS,1	PAVSLAMT,1	SGVFVENG,1	GFAMAEAL,1	HLREYQDL,-1
RQANFLGK,1	AETFYTDG,1	KMMLLAKA,1	SGVFHVNG,1	SGVFVEMP,1	LKGTNESL,-1
PGNFLQSR,1	SGTYYTDS,1	SEEYPIMI,1	SGVFVQNG,1	SGVYHVST,1	RISLPLPN,-1
PLFAGISE,1	SDTYYTQS,1	FQAYPLRE,1	SGVFQVNG,1	SGINFESG,1	PGVRLLQD,-1
ARVLAEAM,1	SDCYYCDS,1	TSLLTLDD,-1	SGIYTVQS,1	GFIGVSYL,1	DSLPLVDT,-1
PLIMANVN,1	SQNYYIDQ,1	KQTFPIQQ,-1	SGVFVVQG,1	SGVHFISR,1	GEESRISL,-1
PYVGSGLY,-1	SDTYYGDS,1	PAILVHTP,-1	GRINVALV,1	SGVYLATD,1	VDFSLADA,-1
LFAGISDW,-1	SQNYPTVQ,1	SKLLATVV,-1	SGVFSVNG,1	SGIYYSVS,1	GKSRLGDL,-1
VVAMPVVI,-1	AETFYVDG,1	LQVLTLNI,-1	SGVFAVTQ,1	SGAFMTRG,1	SRLGDLYE,-1
SRSLYASS,1	SDTYYCDS,1	STLLIENS,-1	SGVYQLSA,1	SGIYLVEN,1	PDLTAALR,-1
SQNYPQVQ,1	SQTYYTVQ,1	ASILPVIP,-1	SGIFVVNG,1	SGIFLETS,1	IQELQAQI,-1
ARNYPIAQ,1	SDTYYTFS,1	PGNLLQSR,1	SGVFVVNG,1	SGNYLVTS,1	IDVDVSKP,-1
SRNYPEVQ,1	SDCYYTDS,1	QGNFLQSR,1	SFVFVVNG,1	SGVFYSRE,1	QELNDRFA,-1
SRVLAEAM,1	SDEYYEDS,1	PRNFLQSR,1	KSRVNVVN,-1	GQDFPMYL,1	TNESLERQ,-1
SRVLAIAM,1	SDLYYTDS,1	GQVNFLGK,1	SRVQVVNG,-1	SGVFTEER,1	KNTRTNEK,-1
SQNYPEAQ,1	SGTYYTGS,1	RRVNFLGK,1	GALTNAVL,-1	SGLYYVTE,1	GPGTASRP,-1
AQVLAEAM,1	SDTYYEDS,1	GQANFLGK,1	VNQALVPK,-1	SGNYFVQG,1	GGPGTASR,-1
ARVLAIAM,1	SDIYYTDS,1	RQVNFLGK,1	MVNQALVP,-1	GEMFFPVL,1	RTNEKVEL,-1
SQNYPEVQ,1	SQTYYIDQ,1	PGNFFQSR,1	TWMVHSLV,-1	SGNYFVET,1	LQEAEEWY,-1
ARNYPQAQ,1	SQTYYTDQ,1	PGNFVQSR,1	MVHSLVPK,-1	GLNMPALV,1	EDIMRLRE,-1
SQNYPIAQ,1	ATIMMITA,1	PGNYLQSR,1	WMVHSLVP,-1	SGTFQVQL,1	NYIDKVRF,-1
SRNYPEAQ,1	SQNYPIDQ,1	PGNFPQSR,1	GVFVNNGL,-1	SGIMFQSA,1	AVEAANYQ,-1
ARNYPEAQ,1	SDTYYTLS,1	RRANFLGK,1	KSGVQVVN,-1	SGIMFESN,1	NDALRQAK,-1
SRNYPIAQ,1	SDTYYTDS,1	TKFLVVQP,1	RVNVVNGK,-1	SGLFTEYG,1	FSLADAIN,-1
ARVLAQAM,1	SQNYYTVQ,1	TKVFVVQP,1	SGALTNAV,-1	SGVMFQTD,1	DLSEAANR,-1
SRNYPIVQ,1	SQNYYIVQ,1	DKVLVVQP,1	VFVNNGLV,-1	SGVFVETS,1	YSLGSALR,-1
TKVLVVQP,1	SQTYYIVQ,1	TKVLFVQP,1	KGSGALTN,-1	GLVAFANL,1	QSLTCEVD,-1
PVILPIQA,1	SDTYYIDS,1	TKILVVQP,1	GLTMVTQL,-1	SGNMLVYS,1	TRSSAVRL,-1
PQVLPVMH,1	SDTYYTGS,1	SQIYPIVQ,1	ALTNAVLV,-1	SGNMVMFG,1	QMREMEEN,-1
SQAYPIVQ,1	SQNPYIVQ,-1	TKVLPVQP,1	GSGAMVNQ,-1	SGSYVEYQ,1	FRQDVDNA,-1
SQCYPIVQ,1	QVIPYNQS,-1	TVVLVVQP,1	TMVTQLVP,-1	SGVHVEYT,1	RSSAVRLR,-1
SQNYPLVQ,1	SDGYYGDS,-1	TKVLIVQP,1	KGSGLTMV,-1	SGVLFVSS,1	SFRQDVDN,-1
SQNYPIFQ,1	GLAAPQFS,1	VKVLVVQP,1	KGSGAMVN,-1	GLVLQEGL,1	NFAVEAAN,-1
SQTYPIVQ,1	KELYPLTS,1	TKVLSVQP,1	SGAMVNQA,-1	SGVMPTMS,1	SQHHDDLE,-1
SQFYPIVQ,1	SLNLPVAK,1	TKVMVVQP,1	GVNVVNGK,-1	SGAYLIQG,1	EMLQREEA,-1

KSRLGDLY,-1	RLREKLQE,-1	EMARHLRE,-1	QEQHVQID,-1	LDLERKVE,-1	SVPGVRLL,-1
NTLQSFRQ,-1	EQLKGQGK,-1	DLTAALRD,-1	SVDFSLAD,-1	IATYRKLL,-1	ENTLQSFR,-1
NETSQHHD,-1	KARVEVER,-1	NMKEEMAR,-1	IEIATYRK,-1	LERKVESL,-1	RVEVERDN,-1
SLGSALRP,-1	LERQMREM,-1	QREEAENT,-1	EIQELQAQ,-1	ANYIDKVR,-1	FLKKLHEE,-1
MARHLREY,-1	TCEVDALK,-1	QDTIGRLQ,-1	SLARLDLE,-1	QQYESVAA,-1	ELQAQIQE,-1
EEEMRELR,-1	QQNKILLA,-1	GQVINETS,-1	IKTVETRD,-1	LGSALRPS,-1	EEEIQELQ,-1
FADLSEAA,-1	QESTEYRR,-1	MKEEMARH,-1	SSRSYVTT,-1	GQGKSRLG,-1	AVRLRSSV,-1
RRQVQSLT,-1	REYQDLLN,-1	TRDGQVIN,-1	FANYIDKV,-1	KSKFADLS,-1	MRELRRQV,-1
NKILLAEL,-1	QDLLNVKM,-1	RNNDALRQ,-1	GTASRPSS,-1	VRFLEQQN,-1	KTVETRDG,-1
STEYRRQV,-1	RPSTSRSL,-1	RLDLERKV,-1	LDSLPLVD,-1	IQNMKEEM,-1	TVETRDGQ,-1
ERDNLAED,-1	ERQMREME,-1	KPDLTAAL,-1	LREKLQEE,-1	EQQNKILL,-1	LPLVDTHS,-1
LEQLKGQG,-1	KLLEGEES,-1	LLEGEESR,-1	SRISLPLP,-1	VTTSTRTY,-1	VEVERDNL,-1
SRSYVTTS,-1	EKLQEEML,-1	ESLERQMR,-1	DLLNVKMA,-1	AQIQEQHV,-1	NNDALRQA,-1
VETRDGQV,-1	QIDVDVSK,-1	HVQIDVDV,-1	DNLAEDIM,-1	SSAVRLRS,-1	MSTRSVSS,-1
ALDIEIAT,-1	KGTNESLE,-1	STRTYSLG,-1	VESLQEEI,-1	MLQREEAE,-1	LAEDIMRL,-1
HSKRTFLI,-1	WYKSKFAD,-1	EWYKSKFA,-1	SALRPSTS,-1	VPGVRLLQ,-1	VAAKNLQE,-1
VRQQYESV,-1	FLEQQNKI,-1	KVELQELN,-1	LNVKMALD,-1	TSQHHDDL,-1	QAQIQEQH,-1
AELEQLKG,-1	EIQNMKEE,-1	EEIAFLKK,-1	EEMLQREE,-1	TNLDSLPL,-1	AALRDVRQ,-1
INETSQHH,-1	LPLPNFSS,-1	SLPLVDTH,-1	DALRQAKQ,-1	RRQVDQLT,-1	RTYSLGSA,-1
LRSSVPGV,-1	QEAEEWYK,-1	TIGRLQDE,-1	QNMKEEMA,-1	LEQQNKIL,-1	AANYQDTI,-1
MFGGPGTA,-1	KLQEEMLQ,-1	AFLKKLHE,-1	LRQAKQES,-1	RSYVTTST,-1	MRLREKLQ,-1
AENTLQSF,-1	ARVEVERD,-1	DRFANYID,-1	TEFKNTRT,-1	NLAEDIMR,-1	DKVRFLEQ,-1
ERKVESLQ,-1	GSALRPST,-1	SVAAKNLQ,-1	DVDVSKPD,-1	ELEQLKGQ,-1	YIDKVRFL,-1
AEDIMRLR,-1	DFSLADAI,-1	LDIEIATY,-1	PLVDTHSK,-1	TNEKVELQ,-1	ATYRKLLE,-1
VSKPDLTA,-1	QNKILLAE,-1	AKNLQEAE,-1	EYQDLLNV,-1	DQLTNDKA,-1	SSSYRRMF,-1
ASSPGGVY,-1	SLPLPNFS,-1	VEAANYQD,-1	YKSKFADL,-1	ALRPSTSR,-1	LTAALRDV,-1
ALKGTNES,-1	VDNASLAR,-1	LQSFRQDV,-1	DVSKPDLT,-1	NDKARVEV,-1	ALRQAKQE,-1
RDGQVINE,-1	FKNTRTNE,-1	EYRRQVQS,-1	CEVDALKG,-1	DIMRLREK,-1	LRPSTSRS,-1
EEMRELRR,-1	VRLRSSVP,-1	NEKVELQE,-1	VINETSQH,-1	SSYRRMFG,-1	KRTFLIKT,-1
ESLQEEIA,-1	RQMREMEE,-1	QHVQIDVD,-1	ANYQDTIG,-1	EAEEWYKS,-1	AANRNNDA,-1
ELQELNDR,-1	YQDTIGRL,-1	EVERDNLA,-1	QYESVAAK,-1	DTHSKRTF,-1	RQVDQLTN,-1
ATRSSAVR,-1	SSSRSYVT,-1	NTRTNEKV,-1	RQDVDNAS,-1	NYQDTIGR,-1	RLLQDSVD,-1
ISLPLPNF,-1	LRRQVDQL,-1	RFANYIDK,-1	SYVTTSTR,-1	LQDEIQNM,-1	KVRFLEQQ,-1
LQEEMLQR,-1	AEEWYKSK,-1	TTSTRTYS,-1	RQQYESVA,-1	NASLARLD,-1	DIEIATYR,-1
QELQAQIQ,-1	DKARVEVE,-1	VKMALDIE,-1	DLYEEEMR,-1	LAELEQLK,-1	MREMEENF,-1
YESVAAKN,-1	QVINETSQ,-1	TYRKLLEG,-1	DNASLARL,-1	KMALDIEI,-1	EMRELRRQ,-1
QEEMLQRE,-1	MEENFAVE,-1	DSVDFSLA,-1	PGTASRPS,-1	QIQEQHVQ,-1	SKPDLTAA,-1
RDVRQQYE,-1	ETNLDSLP,-1	EMEENFAV,-1	YQDLLNVK,-1	TLQSFRQD,-1	ADLSEAAN,-1
LRDVRQQY,-1	EIAFLKKL,-1	ETSQHHDD,-1	LKGQGKSR,-1	EEWYKSKF,-1	TRTNEKVE,-1
NDRFANYI,-1	LQELNDRF,-1	QDSVDFSL,-1	FAVEAANY,-1	EEIQELQA,-1	IMRLREKL,-1
LQAQIQEQ,-1	SLTCEVDA,-1	LIKTVETR,-1	VQIDVDVS,-1	QAKQESTE,-1	RELRRQVD,-1
EAENTLQS,-1	TASRPSSS,-1	STRSVSSS,-1	YASSPGGV,-1	DVDNASLA,-1	KQESTEYR,-1
LREYQDLL,-1	TRTYSLGS,-1	SKFADLSE,-1	RSSVPGVR,-1	TYSLGSAL,-1	DLERKVES,-1
KGQGKSRL,-1	ARLDLERK,-1	EIATYRKL,-1	RKLLEGEE,-1	SSVPGVRL,-1	QVDQLTND,-1
ANRNNDAL,-1	SLERQMRE,-1	NVKMALDI,-1	RMFGGPGT,-1	ELRRQVDQ,-1	SSSSYRRM,-1
LARLDLER,-1	PSSSRSYV,-1	ESRISLPL,-1	REMEENFA,-1	LEGEESRI,-1	DEIQNMKE,-1
QDVDNASL,-1	EVDALKGT,-1	VERDNLAE,-1	PNFSSLNL,-1	VSSSSYRR,-1	VDQLTNDK,-1
EEAENTLQ,-1	GDLYEEEM,-1	FGGPGTAS,-1	TAALRDVR,-1	NLQEAEEW,-1	RTFLIKTV,-1
RSVSSSSY,-1	ELNDRFAN,-1	SEAANRNN,-1	KFADLSEA,-1	QVQSLTCE,-1	TSTRTYSL,-1
AKQESTEY,-1	QGKSRLGD,-1	IQEQHVQI,-1	RQVQSLTC,-1	KVESLQEE,-1	RQAKQEST,-1
KKLHEEEI,-1	KLHEEEIQ,-1	MALDIEIA,-1	VRLLQDSV,-1	FLIKTVET,-1	VDTHSKRT,-1
REKLQEEM,-1	LTNDKARV,-1	YRKLLEGE,-1	GTNESLER,-1	ESVAAKNL,-1	NLDSLPLV,-1
ARHLREYQ,-1	VDVSKPDL,-1	ASRPSSSR,-1	QEEIAFLK,-1	QLKGQGKS,-1	ENFAVEAA,-1
LQDSVDFS,-1	AAKNLQEA,-1	EFKNTRTN,-1	YRRQVQSL,-1	LLNVKMAL,-1	TESIVIWG,-1
LHEEEIQE,-1	KEEMARHL,-1	TNDKARVE,-1	ASLARLDL,-1	LQEEIAFL,-1	LRGTKALT,-1
VELQELND,-1	RRMFGGPG,-1	LYEEEMRE,-1	ALRDVRQQ,-1	EQHVQIDV,-1	HGYQLEKE,-1
QDEIQNMK,-1	DGQVINET,-1	PLPNFSSL,-1	YRRMFGGP,-1	RLRSSVPG,-1	WPLTEEKI,-1
QSFRQDVD,-1	LPNFSSLN,-1	EKVELQEL,-1	RKVESLQE,-1	ETRDGQVI,-1	AIKKKDST,-1
TEYRRQVQ,-1	LTCEVDAL,-1	NRNNDALR,-1	TFLIKTVE,-1	SRPSSSRS,-1	ISPIETVP,-1
EAANYQDT,-1	YEEEMREL,-1	RHLREYQD,-1	TRSVSSSS,-1	RLQDEIQN,-1	SDLEIGQH,-1
EENFAVEA,-1	GRLQDEIQ,-1	LLAELEQL,-1	EEMARHLR,-1	EGEESRIS,-1	PYNTPVFA,-1
VQSLTCEV,-1	EESRISLP,-1	ILLAELEQ,-1	VDALKGTN,-1	NESLERQM,-1	AIFQSSMT,-1
IAFLKKLH,-1	YVTTSTRT,-1	SAVRLRSS,-1	DVRQQYES,-1	LQREEAEN,-1	SVPLDEDF,-1

EKEGKISK,-1	IPSINNET,-1	NTTNQKTE,-1	KYTAFTIP,-1	DFRELNKR,-1	GWKGSPAI,-1
KDSWTVND,-1	LNWASQIY,-1	IPHPAGLK,-1	SESELVNQ,-1	QWTYQIYQ,-1	SINNETPG,-1
NETPGIRY,-1	LVDFRELN,-1	PFKKQNPD,-1	KKKSVTVL,-1	TIPSINNE,-1	DKLVSAGI,-1
WKGSPAIF,-1	TWIPEWEF,-1	VGSDLEIG,-1	VTVLDVGD,-1	TEEKIKAL,-1	GPKVKQWP,-1
GTKALTEV,-1	IKKKDSTK,-1	FQSSMTKI,-1	IVIYQYMD,-1	EDFRKYTA,-1	TEEAELEL,-1
WWTEYWQA,-1	PVFAIKKK,-1	GVYYDPSK,-1	VKQLTEAV,-1	HGVYYDPS,-1	GQHRTKIE,-1
LALQDSGL,-1	VQLGIPHP,-1	HPAGLKKK,-1	KTGKYARM,-1	GLTTPDKK,-1	SPAIFQSS,-1
YARMRGAH,-1	KLGKAGYV,-1	VGKLNWAS,-1	QATWIPEW,-1	QYALGIIQ,-1	NPDIVIYQ,-1
YMDDLYVG,-1	GGNEQVDK,-1	GIRYQYNV,-1	TTNQKTEL,-1	WTVNDIQK,-1	YQIYQEPF,-1
IAEIQKQG,-1	KLLRGTKA,-1	AFTIPSIN,-1	TEMEKEGK,-1	YQLEKEPI,-1	KITTESIV,-1
DDLYVGSD,-1	DFRKYTAF,-1	QLCKLLRG,-1	KILEPFKK,-1	RETKLGKA,-1	GHLVIHPI,-1
GIKVRQLC,-1	PISPIETV,-1	DEDFRKYT,-1	LEPFKKQN,-1	NNETPGIR,-1	LGIPHPAG,-1
WETWWTEY,-1	KQGQGQWT,-1	PEKDSWTV,-1	KNLKTGKY,-1	MEKEGKIS,-1	YVDGAANR,-1
DSQYALGI,-1	KKQNPDIV,-1	PDKSESEL,-1	QKTELQAI,-1	ATWIPEWE,-1	PGMDGPKV,-1
PLDEDFRK,-1	VNQIIEQL,-1	PGHLVIHP,-1	EQVDKLVS,-1	GNEQVDKL,-1	QSSMTKIL,-1
LNKRTQDF,-1	AENREILK,-1	TKWRKLVD,-1	EQLIKKEK,-1	LRWGLTTP,-1	LCKLLRGT,-1
TKALTEVI,-1	GMDGPKVK,-1	GKAGYVTN,-1	LEKEPIVG,-1	KLVGKLNW,-1	DSTKWRKL,-1
KIKALVEI,-1	DHDHPFHG,-1	LYVGSDLE,-1	LPQGWKGS,-1	PIHHHHHH,-1	DHPFHGYQ,-1
RYQYNVLP,-1	NREILKEP,-1	IYQEPFKN,-1	GPENPYNT,-1	QVDKLVSA,-1	GQGQWTYQ,-1
KKKDSTKW,-1	VSAGIRKI,-1	FAIKKKDS,-1	WGKTPKFK,-1	ITTESIVI,-1	KGIGGNEQ,-1
GIPHPAGL,-1	TNKGRQKV,-1	RELNKRTQ,-1	TKILEPFK,-1	TNDVKQLT,-1	QKVVPLTN,-1
HDHPFHGY,-1	ASQIYPGI,-1	ESIVIWGK,-1	PDKKHQKE,-1	EELRQHLL,-1	RKLVDFRE,-1
TDSQYALG,-1	DKSESELV,-1	YQYNVLPQ,-1	PPLVKLWY,-1	HHPGHLVI,-1	GRQKVVPL,-1
SELVNQII,-1	VFAIKKKD,-1	ANRETKLG,-1	IYPGIKVR,-1	LPIQKETW,-1	HHHPGHLV,-1
AGYVTNKG,-1	LIKKEKVY,-1	PIETVPVK,-1	RMRGAHTN,-1	PFKNLKTG,-1	IIQAQPDK,-1
VLPQGWKG,-1	GKTPKFKL,-1	SWTVNDIQ,-1	IKVRQLCK,-1	GSPAIFQS,-1	EVIPLTEE,-1
KPGMDGPK,-1	GKISKIGP,-1	LLRWGLTT,-1	TEAVQKIT,-1	DIVIYQYM,-1	SKIGPENP,-1
EPFKNLKT,-1	QKQGQGQW,-1	EILKEPVH,-1	KVVPLTNT,-1	IWGKTPKF,-1	GYVTNKGR,-1
QGQWTYQI,-1	VDKLVSAG,-1	LRQHLLRW,-1	NDVKQLTE,-1	LVGKLNWA,-1	KLWYQLEK,-1
KLKPGMDG,-1	IGPENPYN,-1	EKIKALVE,-1	QWPLTEEK,-1	ISKIGPEN,-1	GIGGNEQV,-1
KWRKLVDF,-1	VNTPPLVK,-1	VHGVYYDP,-1	VNDIQKLV,-1	ETWETWWT,-1	QAQPDKSE,-1
YVTNKGRQ,-1	GSDLEIGQ,-1	IQKQGQGQ,-1	AWVPAHKG,-1	TKIEELRQ,-1	IPLTEEAE,-1
QDSGLEVN,-1	VIHPISPI,-1	PAHKGIGG,-1	EFVNTPPL,-1	QGWKGSPA,-1	PDKWTVQP,-1
YQEPFKNL,-1	SPIETVPV,-1	EKDSWTVN,-1	IHDHDHPF,-1	FRELNKRT,-1	SAGIRKIL,-1
RTKIEELR,-1	LPEKDSWT,-1	VPLDEDFR,-1	TWETWWTE,-1	NTPVFAIK,-1	YYDPSKDL,-1
IVLPEKDS,-1	KDSTKWRK,-1	TPDKKHQK,-1	KGRQKVVP,-1	VIYQYMDD,-1	KSVTVLDV,-1
SQYALGII,-1	FKNLKTGK,-1	PENPYNTP,-1	ETWWTEYW,-1	ALQDSGLE,-1	TPVFAIKK,-1
YTAFTIPS,-1	EIQKQGQG,-1	RGTKALTE,-1	QEPFKNLK,-1	TPPLVKLW,-1	KISKIGPE,-1
TVQPIVLP,-1	LKTGKYAR,-1	EPVHGVYY,-1	NWASQIYP,-1	IIEQLIKK,-1	VLDVGDAY,-1
GAHTNDVK,-1	VQPIVLPE,-1	LVIHPISP,-1	YVGSDLEI,-1	WTVQPIVL,-1	PLTEEAEL,-1
ETPGIRYQ,-1	TNTTNQKT,-1	LKKKKSVT,-1	KALTEVIP,-1	KEPVHGVY,-1	QLEKEPIV,-1
GIIQAQPD,-1	ELNKRTQD,-1	QYNVLPQG,-1	ENPYNTPV,-1	KQNPDIVI,-1	VQKITTES,-1
AEIQKQGQ,-1	DSWTVNDI,-1	KEPPFLWM,-1	KGSPAIFQ,-1	FRKYTAFT,-1	SSMTKILE,-1
TWWTEYWQ,-1	VIWGKTPK,-1	TEYWQATW,-1	KKSVTVLD,-1	NVLPQGWK,-1	HHHHHPGH,-1
IQKETWET,-1	LTEEAELE,-1	KQLTEAVQ,-1	PFHGYQLE,-1	SIVIWGKT,-1	TTESIVIW,-1
SQIYPGIK,-1	TYQIYQEP,-1	RKYTAFTI,-1	YELHPDKW,-1	KLVDFREL,-1	PQGWKGSP,-1
QIIEQLIK,-1	QLTEAVQK,-1	FVNTPPLV,-1	FKKQNPDI,-1	HHHHHHPG,-1	EVQLGIPH,-1
QNPDIVIY,-1	DGPKVKQW,-1	QHLLRWGL,-1	QLGIPHPA,-1	ARMRGAHT,-1	WASQIYPG,-1
QYMDDLYV,-1	STKWRKLV,-1	NEQVDKLV,-1	VTDSQYAL,-1	VDFRELNK,-1	KVKQWPLT,-1
TTPDKKHQ,-1	GKLNWASQ,-1	KTPKFKLP,-1	HLVIHPIS,-1	TAFTIPSI,-1	PIHDHDHP,-1
TVLDVGDA,-1	TGKYARMR,-1	EPPFLWMG,-1	NPYNTPVF,-1	YALGIIQA,-1	TEVIPLTE,-1
DVKQLTEA,-1	ALGIIQAQ,-1	HTNDVKQL,-1	ILEPFKKQ,-1	QPIVLPEK,-1	LVKLWYQL,-1
GQWTYQIY,-1	ALTEVIPL,-1	YQYMDDLY,-1	IEQLIKKE,-1	EWEFVNTP,-1	IQKLVGKL,-1
IEELRQHL,-1	EAVQKITT,-1	HQKEPPFL,-1	PAGLKKKK,-1	VDGAANRE,-1	KIGPENPY,-1
ETVPVKLK,-1	LLRGTKAL,-1	QKLVGKLN,-1	LGIIQAQP,-1	FTIPSINN,-1	IYQYMDDL,-1
RQKVVPLT,-1	AVQKITTE,-1	LKEPVHGV,-1	EPFKKQNP,-1	ELRQHLLR,-1	HKGIGGNE,-1
KWTVQPIV,-1	NDIQKLVG,-1	PIQKETWE,-1	MRGAHTND,-1	HLLRWGLT,-1	WRKLVDFR,-1
PEWEFVNT,-1	WGLTTPDK,-1	KHQKEPPF,-1	GKYARMRG,-1	AGLKKKKS,-1	MDDLYVGS,-1
KTELQAIY,-1	EMEKEGKI,-1	DKWTVQPI,-1	HPISPIET,-1	IETVPVKL,-1	GAANRETK,-1
HPGHLVIH,-1	INNETPGI,-1	PKFKLPIQ,-1	LKPGMDGP,-1	QPDKSESE,-1	NLKTGKYA,-1
EYWQATWI,-1	NTPPLVKL,-1	WQATWIPE,-1	PLTNTTNQ,-1	LEIGQHRT,-1	EIGQHRTK,-1
KQWPLTEE,-1	PKVKQWPL,-1	KKHQKEPP,-1	QHRTKIEE,-1	WEFVNTPP,-1	VKQWPLTE,-1
VYYDPSKD,-1	ILKEPVHG,-1	PGIKVRQL,-1	SMTKILEP,-1	SVTVLDVG,-1	LVSAGIRK,-1

QKETWETW,-1	DGAANRET,-1	QMIFEEHG,1			
GLKKKKSV,-1	PGIRYQYN,-1	GWILAEHG,1			
PLVKLWYQ,-1	KSESELVN,-1	QAIYLALQ,1			
KKKKSVTV,-1	KETWETWW,-1	DSADAEED,1			
EEKIKALV,-1	ESELVNQI,-1	SKDLIAEI,1			
VIPLTEEA,-1	KLPIQKET,-1				
KKDSTKWR,-1	REILKEPV,-1				
IPEWEFVN,-1	VKLKPGMD,-1				
WEVQLGIP,-1	LTTPDKKH,-1				
KLNWASQI,-1	QKITTESI,-1				
TPKFKLPI,-1	WTYQIYQE,-1				
YPGIKVRQ,-1	IGGNEQVD,-1				
HRTKIEEL,-1	RWGLTTPD,-1				
NKGRQKVV,-1	LTEEKIKA,-1				
MDGPKVKQ,-1	TPGIRYQY,-1				
WVPAHKGI,-1	QGQGQWTY,-1				
HPFHGYQL,-1	VLPEKDSW,-1				
IHHHHHHP,-1	KLVSAGIR,-1				
MTKILEPF,-1	IQAQPDKS,-1				
ELVNQIIE,-1	HPDKWTVQ,-1				
GYQLEKEP,-1	PIVLPEKD,-1				
KYARMRGA,-1	VPVKLKPG,-1				
YNTPVFAI,-1	PVHGVYYD,-1				
VVPLTNTT,-1	RQLCKLLR,-1				
DIQKLVGK,-1	QKEPPFLW,-1				
LQDSGLEV,-1	LTNTTNQK,-1				
KVRQLCKL,-1	QIYPGIKV,-1				
PLTEEKIK,-1	LTEAVQKI,-1				
IKALVEIC,-1	VPAHKGIG,-1				
KEGKISKI,-1	RQHLLRWG,-1				
PHPAGLKK,-1	NRETKLGK,-1				
LTEVIPLT,-1	KFKLPIQK,-1				
QLIKKEKV,-1	AHKGIGGN,-1				
ENREILKE,-1	TNQKTELQ,-1				
VKLWYQLE,-1	FHGYQLEK,-1				
IGQHRTKI,-1	NQIIEQLI,-1				
DLEIGQHR,-1	LVNQIIEQ,-1				
IVIWGKTP,-1	DKKHQKEP,-1				
KIEELRQH,-1	PSINNETP,-1				
LAWVPAHK,-1	PDIVIYQY,-1				
LGKAGYVT,-1	WYQLEKEP,-1				
AQPDKSES,-1	TVNDIQKL,-1				
PVKLKPGM,-1	FSVPLDED,-1				
LHPDKWTV,-1	IFQSSMTK,-1				
LDEDFRKY,-1	WTEYWQAT,-1				
YNVLPQGW,-1	TKLGKAGY,-1				
CKLLRGTK,-1	ETKLGKAG,-1				
VRQLCKLL,-1	TVPVKLKP,-1				
VTNKGRQK,-1	PAIFQSSM,-1				
IHPISPIE,-1	FKLPIQKE,-1				
RGAHTNDV,-1	NQKTELQA,-1				
IVTDSQYA,-1	KAGYVTNK,-1				
VPLTNTTN,-1	WIPEWEFV,-1				
IRYQYNVL,-1	HDHDHPFH,-1				
EGKISKIG,-1	ELELAENR,1				
AHTNDVKQ,-1	GWILGEHG,1				
HHHHPGHL,-1	HYGFPTYG,1				
LWYQLEKE,-1	GDAYFSVP,1				
DLYVGSDL,-1	LWMGYELH,1				
ELHPDKWT,-1	EKVYLAWV,1				
QIYQEPFK,-1	VEICTEME,1				
AANRETKL,-1	TQDFWEVQ,1				
YWQATWIP,-1	LEVNIVTD,1				
