# Supplementary material for: Prediction of HIV-1 protease cleavage site using a combination of sequence, structural, and physicochemical features
Source: BMC Bioinformatics. 2016 Dec 23;17(Suppl 17):478. doi: 10.1186/s12859-016-1337-6 (PMC5259813; doi:10.1186/s12859-016-1337-6)
Supplement: Additional file 3: — The Schilling dataset. (PDF 401 kb) [file 12859_2016_1337_MOESM3_ESM.pdf]

Additional File 3: The Schilling dataset

AAAGKSGG,-1	ASASASAE,-1	DNGSGMCK,-1	EEVMPVLE,1	EQVANSAF,-1	GAFTCYLD,-1
AAAVDAGM,-1	ASATFPMQ,-1	DPEVTDEE,-1	EFDGGVVM,-1	EQVELPDG,-1	GAGSVPAI,-1
AAGKSGGG,-1	ASCEDIAR,-1	DPGDSDII,-1	EFQTKTEE,-1	ESGAPGEE,-1	GAGTYGAS,-1
AALALEYG,1	ASDVLELT,1	DQGGLEAV,-1	EGDQKPLA,-1	ESIDGQPG,1	GAKFKKYE,-1
AANDGPMP,-1	ASSTTSTG,-1	DQKPLAQR,-1	EGEEEEEE,-1	ESISKTNR,-1	GAPGEEQR,-1
AASAAAVD,-1	ATEDKEAL,-1	DQVANSAF,-1	EGGGAKFK,-1	ESKEQVAN,-1	GAPGGGKR,-1
AATEPELL,-1	ATEPELLD,-1	DREDLVYQ,1	EGIVLGGG,-1	ESPIEQLE,-1	GASATFPM,-1
AAVDAGMA,-1	ATFPMQCS,-1	DSFGGDAQ,-1	EGLKMVEK,-1	ESTGSIAK,-1	GASCEDIA,-1
ADGYAVDR,-1	ATVKKGKP,-1	DTAVLVID,1	EGSLQNGV,-1	ETAKGDYP,-1	GASSTTST,-1
ADKVPKTA,-1	ATVTKPVG,-1	DTIEIITD,-1	EGVLKVIK,-1	ETGDAGAS,-1	GAVEKGVP,-1
ADLQNDEV,-1	AVCKAGAV,-1	DTSVDLEE,-1	EHGLVVIA,1	ETGEALSE,-1	GCILVVAA,1
ADLTDTAQ,-1	AVDAGMAM,-1	DVDGTLTA,-1	EIAASKCR,-1	ETKDQVAN,-1	GCVVGTKK,-1
ADTSVDLE,-1	AVDGEPLG,-1	DVEALENS,1	EIDNAPEE,-1	ETQNGDVS,-1	GDAGASAT,-1
AEFQTKTE,-1	AVDLVEEE,1	DVFSPIGE,-1	EIEAIELP,1	ETTAGEQS,-1	GDAQADEG,-1
AEGGGAKF,-1	AVEKGVPL,-1	DVGQLGLG,-1	EIIAFSDR,-1	EVEQVELP,-1	GDEMVSLK,1
AEKELEER,-1	AVPETLKK,-1	DVSEETMG,-1	EIIAFSNR,-1	EVLVAGFG,-1	GDEPGPQR,-1
AELKALLQ,-1	CIMLSGET,1	DVVMAATE,1	EILSSLLP,-1	EVPAVPET,-1	GDKNGGTR,-1
AENQAESR,-1	CKAGAVEK,-1	DWVFVSSK,1	EIPALSVS,-1	EVQALDDT,-1	GDQKPLAQ,-1
AENVVIVA,-1	CKAGFAGD,-1	DYASVEVS,1	EIQEFVKD,1	EVQDAELK,-1	GDVGQLGL,-1
AEVEQVEL,-1	CLIINNHN,-1	DYDSVEQD,1	EIVLPENA,1	EVSLADLQ,-1	GDVSEETM,-1
AFMGPLKK,-1	CLNFIEEN,1	DYPLEAVR,-1	EKAQDEIP,-1	EVTDEEIK,-1	GDYPLEAV,-1
AFTCYLDA,-1	CPSGPQPR,-1	EADDDKKR,-1	EKDKPSLR,-1	EYEQLSSE,-1	GEAFTILS,1
AGASATFP,-1	CPTEIIAF,-1	EAEGQQTR,1	EKDQDGGR,-1	EYGAGTYG,-1	GEALSEET,-1
AGAVEKGV,-1	CVVGTKKR,-1	EAENQAES,-1	EKGVPLYR,-1	EYGASCED,-1	GEEEEEEE,-1
AGFAGDDA,-1	CYLDAGLA,-1	EAIYDICR,-1	EKKAFMGP,-1	EYIEENKY,-1	GESKEQVA,-1
AGIRLLPS,1	DADLTDTA,-1	EAKELVGR,-1	EKKKEVPA,-1	FADKVPKT,-1	GETAKGDY,-1
AGKSGGGG,-1	DAELKALL,-1	EALKKQLP,-1	EKQQQDQV,-1	FAGDDAPR,-1	GETKDQVA,-1
AGMAMAGQ,-1	DAGASATF,-1	EALSEETT,-1	EKSLGLSK,-1	FDGGVVMG,-1	GFAGDDAP,-1
AGQSPVLR,-1	DAGMAMAG,-1	EANFPANV,-1	EKSVSQKV,-1	FDIAVDGE,-1	GFGFVSFE,-1
AGSVPAIQ,-1	DAQADEGQ,-1	EAPIYELT,-1	EKVFLAQK,1	FDVDGTLT,-1	GFSLLATE,1
AGTYGASS,-1	DCAFMVDN,1	EAPLNPKA,-1	EKVLATVT,1	FEEYGKID,1	GFTPGGGG,-1
AIIDPGDS,-1	DCIMLSGE,1	EAVLTEEL,1	ELCHQWLR,-1	FETGDAGA,-1	GFVSFERH,1
AINQGGLT,-1	DDDYVEEG,1	EDKEALKK,-1	ELDEEEQL,-1	FEVLQIDP,1	GGAKFKKY,-1
AKFKKYEE,-1	DDLDFETG,1	EDQGGLEA,-1	ELDGAPGG,-1	FEVSLADL,1	GGDAQADE,-1
AKGDYPLE,-1	DEDGLELD,1	EEADDDKK,-1	ELEMNSDL,-1	FEYIEENK,-1	GGDKNGGT,-1
AKLAEQAE,-1	DEEEQLLR,-1	EEAENQAE,-1	ELETLRAQ,1	FGDFNLPR,-1	GGGAKFKK,-1
AKVSTASV,-1	DEGVLKVI,-1	EEAKELVG,-1	ELKALLQS,-1	FGFVSFER,-1	GGGCALLR,-1
ALCLFDVD,1	DEIPALSV,-1	EEDREVAA,1	ELKKEEIK,-1	FGGDAQAD,-1	GGGGSSSS,-1
ALDGACGR,-1	DELDQKMR,-1	EEEAENQA,-1	ELLDDQEA,-1	FIEDQGGL,-1	GGGGSSVS,-1
ALEKDKPS,-1	DESTGSIA,-1	EEEEEEEE,-1	ELPDGKKR,-1	FKKYEEID,-1	GGGSGGLA,-1
ALIAAQYS,1	DEVLVAGF,-1	EEEEEEEG,-1	ELPMDPKL,-1	FLKANQKK,-1	GGGSSSSG,-1
ALKKQLPG,-1	DEYEQLSS,-1	EEEEEEGG,-1	ELSLLEKS,1	FLLLTLEQ,1	GGGSSVSM,-1
ALLQSSAS,-1	DFVMLKGC,1	EEEEEGGL,-1	ELSLLGKK,-1	FMGPLKKD,-1	GGLTSVAV,-1
ALSEETTA,-1	DGAPGGGK,-1	EEEEGGLR,-1	ELTDDNFE,-1	FNLQILPK,1	GGSCPSGP,-1
AMAGQSPV,-1	DGDEPGPQ,-1	EEEMTVVE,1	ELTSQFTG,-1	FPANVMDV,-1	GGSGGLAS,-1
ANDGPMPQ,-1	DGGVVMGS,-1	EEESGAPG,-1	EMADGYAV,-1	FPMQCSAL,-1	GGSSSSGG,-1
ANFPANVM,-1	DGPMPQTR,-1	EEGEEEEE,-1	EMEKQQQD,-1	FPSLILQK,1	GGSSVSMI,-1
ANSAFVER,-1	DGQEAPIY,-1	EEGIVLGG,-1	EMNPGYEV,-1	FQTKAEVE,-1	GGTTMYPG,-1
ANVMDVIA,-1	DGTLTAPR,-1	EEIAASKC,-1	EMNSDLKA,-1	FQTKTEEE,-1	GGVVMGSD,-1
APIYELTS,-1	DIAALVVD,1	EEIDNAPE,-1	ENAEKELE,-1	FQTMEEKK,1	GIIYGTDG,-1
APLNPKAN,-1	DIAVDGEP,-1	EEKKAFMG,-1	ENDEVLVA,-1	FSILNTPK,1	GIVLGGGC,-1
APPGKEKQ,-1	DISPKQDE,-1	EEKKKEVP,-1	ENSAGATY,-1	FTCYLDAG,-1	GKIDTIEI,-1
AQADEGQA,-1	DKEALKKQ,-1	EELDEEEQ,-1	ENVVIVAS,-1	FTPGGGGS,-1	GKSGGGGS,-1
AQDEIPAL,-1	DKVPKTAE,-1	EELREILS,1	EPDTKLAR,-1	FVCPTEII,-1	GLGENVME,-1
AQKMMIGR,-1	DLALLEDL,1	EENDEVLV,-1	EPELLDDQ,-1	FVKDVFSP,-1	GLKMVEKD,-1
AQNRAEIK,1	DLEPTVID,-1	EEPDTKLA,-1	EPTLDELD,-1	FVSFERHE,-1	GLSKGNKY,-1
AQYSGAQV,-1	DLQNDEVA,-1	EESGAPGE,-1	EPTVIDEV,-1	FVTEEDKR,-1	GLTSVAVR,-1
ARALDGAC,-1	DLTDTAQT,-1	EESPIEQL,-1	EQDGDEPG,-1	FYSDSFGG,1	GLVLTLGQ,1
ARVLTVIN,1	DMVMATVK,1	EETGEALS,-1	EQKNIAVE,-1	GAAMVEMA,1	GMAMAGQS,-1
ASAAAVDA,-1	DNEAIYDI,-1	EETTAGEQ,-1	EQLSSEAL,-1	GADTSVDL,-1	GMCKAGFA,-1

GMCQQLPR,-1	ISKMLFVE,-1	KYSAQGER,-1	LQNGVTAA,-1	NSAGATYI,-1	QEYGAGTY,-1
GMNFKTPR,-1	ISKMVPTS,-1	LADLQNDE,-1	LRAQLLQR,-1	NSDLKAQL,-1	QFQLYEEP,1
GMVMMVPG,1	ISPKQDEG,-1	LAIIDPGD,-1	LSDPKKKT,-1	NTPKKLGN,-1	QGDVGQLG,-1
GNKYSAQG,-1	ITGESKEQ,-1	LAQKMMIG,-1	LSEETTAG,-1	NVELSLLG,1	QGGLEAVR,-1
GQEAPIYE,-1	ITGETKDQ,-1	LASASASA,-1	LSGETAKG,-1	NVMDVIAR,-1	QGGLTSVA,-1
GQGDVGQL,-1	ITQDLLET,1	LATEDKEA,-1	LSGGTTMY,-1	NVVIVASQ,-1	QIDPEVTD,-1
GQLGLGEN,-1	IVLGGGCA,-1	LDAWLEMN,1	LSKGNKYS,-1	PAAFTEAE,1	QKASNLKR,-1
GQPGAFTC,-1	IYELTSQF,-1	LDDQEAKR,-1	LSKPSKGQ,-1	PANVMDVI,-1	QKNIAVET,-1
GRAMQVAK,1	IYGTDGQE,-1	LDEEEQLL,-1	LSLLGKKK,-1	PAVPETLK,-1	QKTESISK,-1
GRGYFEYI,1	KAEVEQVE,-1	LDELDQKM,-1	LSLLQDSG,-1	PEEMVTFK,1	QKVAAAMP,-1
GRVFEVSL,1	KAFMGPLK,-1	LDFTFVCP,1	LSNLQVTQ,1	PEESPIEQ,-1	QLGLGENV,-1
GSCPSGPQ,-1	KAGAVEKG,-1	LDGAPGGG,-1	LSSEALEA,-1	PEIEEVQA,1	QLPGVKSE,-1
GSGGLASA,-1	KAGFAGDD,-1	LDNLVAIL,1	LSSLLPFV,-1	PELLDDQE,-1	QLSSEALE,-1
GSGMCKAG,-1	KALLQSSA,-1	LEDLEKQR,-1	LTDDNFES,-1	PENAEKEL,-1	QNDEVAFR,-1
GSLQNGVT,-1	KAQDEIPA,-1	LEERILGA,1	LTDTAQTR,-1	PETLKKKR,-1	QNEGLTID,1
GSSSSGGG,-1	KDADLTDT,-1	LEGDQKPL,-1	LTELSMQD,1	PEVTDEEI,-1	QNGDVSEE,-1
GSSVSMIA,-1	KDQVANSA,-1	LEGLKMVE,-1	LTIDLKNF,-1	PGAFTCYL,-1	QNGVTAAD,-1
GSVPAIQR,-1	KDVFSPIG,-1	LEKDKPSL,-1	LTSQFTGL,-1	PGAGSVPA,-1	QNPIAQPL,-1
GTDGQEAP,-1	KEALKKQL,-1	LEKSLGLS,-1	LVIDNGSG,-1	PGDSDIIR,-1	QPGAFTCY,-1
GTNAAEFQ,1	KEQVANSA,-1	LEKSVSQK,-1	LVIGGGSG,-1	PGGGGSSV,-1	QPTVGMNF,-1
GTTMYPGI,-1	KEVPAVPE,-1	LELDGAPG,-1	LVNQNPIA,-1	PGIFYSDS,1	QQQDQVDR,-1
GTYGASST,-1	KFKKYEEI,-1	LELEMNSD,-1	LVTVAAAG,-1	PGVKSEGK,-1	QQTRAAAG,-1
GVKSEGKR,-1	KGCDVVVI,1	LELKKEEI,-1	LVVDNGSG,-1	PGYEVAPR,-1	QSERQKAS,1
GVLKVIKR,-1	KGCVVGTK,-1	LELTDDNF,-1	LVYAPPGK,-1	PIAQPLAS,-1	QTKAEVEQ,-1
GVSLAVCK,1	KGDYPLEA,-1	LEMNPGYE,-1	LVYQAKLA,-1	PIEQLEER,-1	QTKTEEEE,-1
GVVMGSDS,-1	KGIIYGTD,-1	LEMNSDLK,-1	LYANTVLS,1	PIYELTSQ,-1	QTQKENLR,-1
GWELIEPT,1	KGNKYSAQ,-1	LENSAGAT,-1	MADGYAVD,-1	PKKLGNSL,-1	QTRAAAGR,-1
GYCLIINN,-1	KHIYYITG,1	LEPTVIDE,-1	MAGQSPVL,-1	PKQDEGVL,-1	QVAKVSTA,-1
HMVSDEYE,1	KIDTIEII,-1	LEQKNIAV,-1	MAMAGQSP,-1	PKSAKQKE,-1	QVANSAFV,-1
IAPDTSPR,-1	KKAFMGPL,-1	LETKGEPR,-1	MCKAGFAG,-1	PKTAENFR,-1	QVELPDGK,-1
IAQPLASR,-1	KKEEIKQR,-1	LEVMVRIR,-1	MDPKLNKR,-1	PKVFFIQA,1	QVPRVMST,-1
IAVDGEPL,-1	KKEVPAVP,-1	LEYGASCE,-1	MEDRLQAL,1	PLMMYISK,1	QVTQPTVG,-1
IAVETDVR,-1	KKGKPELR,-1	LFADVVPK,1	MEKQQQDQ,-1	PLNPKANR,-1	QYSGAQVR,-1
IDNAPEER,-1	KKKEVPAV,-1	LGADTSVD,-1	MFAAEETG,1	PMDPKLNK,-1	RALDGACG,-1
IDNGSGMC,-1	KKLGNSLL,-1	LGENVMER,-1	MGPLKKDR,-1	PMEGVDIS,1	RAVFVDLE,1
IDPEVTDE,-1	KKQLPGVK,-1	LGGGCALL,-1	MLFVEPIL,-1	PMQCSALR,-1	RFDMAENV,1
IDPGDSDI,-1	KKYEEIDN,-1	LGLGENVM,-1	MNIGSDKL,1	PPGKEKQR,-1	RGYCLIIN,-1
IDTIEIIT,-1	KLAEQAER,-1	LGLSKGNK,-1	MNPGYEVA,-1	PRVMSTQR,1	RHGLYEKK,1
IEDQGGLE,-1	KLDFLEGD,1	LGQGDVGQ,-1	MNSDLKAQ,-1	PSAFLELK,1	RLLRGGSC,1
IEENDEVL,-1	KLGNSLLR,-1	LIEMEKQQ,1	MQDEELMK,-1	PSGMCQQL,-1	RSFRARAL,1
IEENKYSR,-1	KMLFVEPI,-1	LIINNHNF,-1	MVDNEAIY,-1	PSPREMIN,1	RVCTLAII,1
IELPMDPK,-1	KMVEKDQD,-1	LIYDFIED,1	MVEKDQDG,-1	PTEIIAFS,-1	RVMSTQRV,-1
IEMEKQQQ,-1	KMVPTSDK,-1	LKALLQSS,-1	MVPGAGSV,-1	PTILEEAK,1	SAAAVDAG,-1
IEPTLDEL,-1	KNIAVETD,-1	LKANQKKR,-1	MVPTSDKG,-1	PTLDELDQ,-1	SAGATYIR,-1
IEYYFGDF,1	KPSKGQKR,-1	LKGCVVGT,-1	MYPGIADR,-1	PTVFFDIA,1	SASASAER,-1
IGGGSGGL,-1	KPVGGDKN,-1	LKKEEIKQ,-1	NAEKELEE,-1	PTVGMNFK,-1	SATFPMQC,-1
IGILVEKA,1	KQDEGVLK,-1	LKKQLPGV,-1	NDEVLVAG,-1	PTVIDEVR,-1	SCPSGPQP,-1
IIDPGDSD,-1	KQLPGVKS,-1	LKMVEKDQ,-1	NDGPMPQT,-1	PVGGDKNG,-1	SDEYEQLS,1
IINNHNFA,-1	KQQQDQVD,-1	LLDDQEAK,-1	NEAIYDIC,-1	PVLEEKER,-1	SDLKAQLR,-1
IIYGTDGQ,-1	KSAKQKER,-1	LLELEMNS,1	NFPANVMD,-1	PVLLTEAP,1	SDPKKKTR,-1
ILAADEST,1	KSEMEVQD,1	LLETKGEP,-1	NGDVSEET,-1	PYEFPEES,1	SDSFGGDA,-1
ILGADTSV,-1	KSGGGGSS,-1	LLGKKKKR,-1	NGSGMCKA,-1	QADEGQAR,-1	SEALEAAR,-1
ILPKSAKQ,-1	KSIYYITG,1	LLPSGMCQ,-1	NGVTAADR,-1	QAKLAEQA,-1	SEENYQAL,1
ILQKTESI,-1	KSLGLSKG,-1	LLQDSGEV,-1	NHINVELS,1	QALDADFR,-1	SEETMGSR,-1
ILSDPKKK,-1	KSVSQKVA,-1	LLQSSASR,-1	NIAVETDV,-1	QALDDTER,-1	SEETTAGE,-1
ILSSLLPF,-1	KTEEEEID,-1	LPGVKSEG,-1	NKYSAQGE,-1	QALEKDKP,-1	SENMEEEA,1
INNHNFAK,-1	KTESISKT,-1	LPKSAKQK,-1	NLALAASA,1	QDAELKAL,-1	SETQNGDV,-1
INQGGLTS,-1	KVAAAMPV,-1	LPMDPKLN,-1	NLTLSETQ,1	QDEELMKR,-1	SFELFADK,1
INQTQKEN,-1	KVEEIAAS,-1	LPSGMCQQ,-1	NPGYEVAP,-1	QDEGVLKV,-1	SFGGDAQA,-1
IPALSVSR,-1	KVPKTAEN,-1	LQALEKDK,-1	NPIAQPLA,-1	QDEIPALS,-1	SGAPGEEQ,-1
IQDGYLSL,1	KVSTASVG,-1	LQDSGEVR,-1	NQGGLTSV,-1	QDGDEPGP,-1	SGETAKGD,-1
IQIMKVEE,1	KWEGLVYA,1	LQKTESIS,-1	NQNPIAQP,-1	QDGYLSLL,1	SGEYFLKA,1
IRLRELCH,1	KYEEIDNA,-1	LQNDEVAF,-1	NQTQKENL,-1	QEAPIYEL,-1	SGFTPGGG,-1

SGGGGSSS,-1	TILSDPKK,-1	VEQDGDEP,-1	YEGSLQNG,-1		
SGGLASAR,-1	TIMAVEFD,1	VEQVELPD,-1	YEKKKTSR,-1		
SGGTTMYP,-1	TKAEVEQV,-1	VEVSKLKR,-1	YELTSQFT,-1		
SGMCKAGF,-1	TKDQVANS,-1	VFFDIAVD,1	YEQLSSEA,-1		
SGMCQQLP,-1	TKILAEGG,1	VFSPIGER,-1	YFEYIEEN,1		
SKDADLTD,-1	TKPVGGDK,-1	VGGDKNGG,-1	YGAGTYGA,-1		
SKEQVANS,-1	TKTEEEEI,-1	VGMNFKTP,-1	YGASCEDI,-1		
SKGNKYSA,-1	TLDELDQK,-1	VGQLGLGE,-1	YGASSTTS,-1		
SKMLFVEP,1	TLEQKNIA,-1	VIAPDTSP,-1	YGTDGQEA,-1		
SKMVPTSD,-1	TLGQGDVG,-1	VIDNGSGM,-1	YIEENKYS,-1		
SKPSKGQK,-1	TLVESSTS,1	VIGGGSGG,-1	YIEQTLVT,1		
SLADLQND,-1	TLVTVAAA,-1	VINQTQKE,-1	YISKMVPT,-1		
SLGLSKGN,-1	TMYPGIAD,-1	VIPAGVPR,-1	YITGESKE,-1		
SLKDYCTR,-1	TPGGGGSS,-1	VISKMLFV,-1	YITGETKD,-1		
SLLGKKKK,-1	TPKKLGNS,-1	VIVASQKR,-1	YLDAGLAR,-1		
SLLQDSGE,-1	TQNGDVSE,-1	VKDVFSPI,-1	YLSLLQDS,-1		
SLQNGVTA,-1	TQPTVGMN,-1	VKKGKPEL,-1	YQAKLAEQ,-1		
SMQDEELM,-1	TRAAVEEG,1	VLGGGCAL,-1	YQALDADF,-1		
SNIFYEGS,1	TRVALEKS,1	VLNFYEAN,1	YQELLVNQ,1		
SPIEQLEE,-1	TSGFTPGG,-1	VLQALEGL,1	YSDSFGGD,-1		
SPKQDEGV,-1	TSQFTGLK,-1	VLSGGTTM,-1			
SQALLELE,1	TSVDLEET,-1	VLSKPSKG,-1			
SQEFLASA,1	TTAGEQST,-1	VLVAGFGR,-1			
SQKVAAAM,-1	TTMYPGIA,-1	VMSTQRVA,-1			
SSEALEAA,-1	TVAAAGKS,-1	VNQNPIAQ,-1			
SSKDADLT,-1	TVFFDIAV,1	VPAVPETL,-1			
SSLLPFVR,-1	TVGMNFKT,-1	VPETLKKK,-1			
SSSSGGGR,-1	TVINQTQK,-1	VPGAGSVP,-1			
SSTSGFTP,-1	TVKKGKPE,-1	VPKTAENF,-1			
SSTTSTGR,-1	TVLSGGTT,-1	VPRVMSTQ,-1			
SSVSMIAS,-1	TVTKPVGG,-1	VPTSDKGR,-1			
STGSIAKR,-1	TVVEEADD,-1	VQALDDTE,-1			
STSGFTPG,-1	TYGASSTT,-1	VQDAELKA,-1			
SVDLEETG,-1	VAAAGKSG,-1	VSEETMGS,-1			
SVSMIASR,-1	VAAAMPVR,-1	VSFERHED,-1			
SVSQKVAA,-1	VAANDGPM,-1	VSLADLQN,-1			
SWTGFQTK,1	VAILDINR,-1	VSLKDYCT,-1			
SYDLVLSK,1	VAINFVTE,1	VSQKVAAA,-1			
SYDYLVIG,1	VAKVSTAS,-1	VSSKDADL,-1			
SYTAQEYG,1	VANSAFVE,-1	VSTASVGR,-1			
TAGEQSTR,-1	VCKAGAVE,-1	VTDEEIKK,-1			
TAKGDYPL,-1	VCPTEIIA,-1	VTFKKTKR,-1			
TCYLDAGL,-1	VDAGMAMA,-1	VTKPVGGD,-1			
TDDNFESR,-1	VDGEPLGR,-1	VTQPTVGM,-1			
TDEEIKKR,-1	VDGTLTAP,-1	VTVAAAGK,-1			
TDGQEAPI,-1	VDISPKQD,-1	VVAANDGP,-1			
TEAELEER,-1	VDLEETGR,-1	VVDNGSGM,-1			
TEAPLNPK,-1	VDLEPTVI,-1	VVEEADDD,-1			
TEDKEALK,-1	VDNEAIYD,-1	VVIAPDTS,-1			
TEEEEIDR,-1	VDNGSGMC,-1	VVIPAGVP,-1			
TEELDEEE,-1	VEEADDDK,-1	VVIVASQK,-1			
TEIIAFSD,-1	VEEESGAP,-1	VVMGSDSR,-1			
TEIIAFSN,-1	VEEGEEEE,-1	VVPKTAEN,-1			
TEPELLDD,-1	VEEGIVLG,-1	VVVIPAGV,-1			
TESISKTN,-1	VEEIAASK,-1	VYAPPGKE,-1			
TFPMQCSA,-1	VEEKKKEV,-1	VYQAKLAE,-1			
TGDAGASA,-1	VEFDGGVV,-1	WKALIEME,1			
TGEALSEE,-1	VEKAQDEI,-1	YAFKAINQ,1			
TGESKEQV,-1	VEKDQDGG,-1	YAPPGKEK,-1			
TGETKDQV,-1	VEKGVPLY,-1	YCLIINNH,-1			
TGGRLEVM,1	VELPDGKK,-1	YEANFPAN,-1			
TIDLKNFR,-1	VELSLLGK,-1	YEEIDNAP,-1			
TIEIITDR,-1	VEMADGYA,-1	YEEPDTKL,-1			
