# Supplementary material for: Prediction of HIV-1 protease cleavage site using a combination of sequence, structural, and physicochemical features
Source: BMC Bioinformatics. 2016 Dec 23;17(Suppl 17):478. doi: 10.1186/s12859-016-1337-6 (PMC5259813; doi:10.1186/s12859-016-1337-6)
Supplement: Additional file 4: — The Impens dataset. (PDF 1093 kb) [file 12859_2016_1337_MOESM4_ESM.pdf]

Additional File 4: The Impens dataset

AAAAAPAK,-1	ADVMIATQ,1	AGFQDIPK,-1	ALDLGGSS,-1	APVAAATT,-1	AVADVTTP,-1
AAAAPAKV,-1	ADVTTPQT,-1	AGGAPSVG,-1	ALDLGGTN,-1	APVLLDAL,-1	AVAGGAPS,-1
AAAELGAR,-1	ADYKPPAT,-1	AGGDGAEA,-1	ALDNVDAR,-1	APVNVTTE,-1	AVANDEEL,-1
AAAPAKVE,-1	AEADGAGL,-1	AGGMHTVC,-1	ALEGVVLS,1	APVVPQAL,-1	AVAVTMGP,-1
AAAPVAAA,-1	AEAGGMHT,-1	AGGPGPGG,-1	ALELLFDQ,1	AQEGVILD,1	AVDAVIAE,-1
AAAPVVPQ,-1	AEAILAAD,-1	AGGVMTAL,-1	ALESPERP,1	AQESVGIY,-1	AVDGEPLG,-1
AAATTAAP,-1	AEALGTLM,-1	AGHQEEAK,-1	ALGGNSSP,-1	AQHYQDTL,-1	AVDLPAVS,1
AADMLGSY,-1	AEAMNYEG,-1	AGIMDHEE,-1	ALGIAQPK,-1	AQIAAENE,-1	AVDVLVSS,-1
AAEIEGAG,-1	AEAYLGYP,1	AGIVGNLR,-1	ALIEMEKQ,1	AQIDPVEK,-1	AVEAANYQ,-1
AAELEMEL,-1	AECFDEIT,-1	AGKDDYVK,-1	ALILHDDE,-1	AQIIEACD,-1	AVEVFEGE,1
AAELVALA,1	AEDGPPEL,-1	AGKVDDAR,-1	ALINAAIQ,-1	AQNDLIWN,1	AVFGEEGL,-1
AAEQGRPP,-1	AEDKENYK,-1	AGPDTNGS,-1	ALIQQATT,-1	AQNLMQSV,-1	AVHDAILE,-1
AAEVVHPG,-1	AEDMEEEQ,1	AGPTALLA,-1	ALKDDSWL,-1	AQNPANMS,-1	AVHVLCNS,-1
AAGAVASY,-1	AEDNADTL,-1	AGQGEVLV,-1	ALLAHEIG,1	AQPVLVFK,-1	AVIAELKK,-1
AAGDLAPI,-1	AEEAEEVG,1	AGTPMFVV,-1	ALLDAVTQ,-1	AQQVLWNC,-1	AVIPGGSS,-1
AAGGDGAE,-1	AEEELQQA,1	AGVAPESF,-1	ALLDGSNV,-1	AQTEALMR,-1	AVISASAS,-1
AAGIVGNL,-1	AEEKAPVK,-1	AGVFMASH,1	ALLFVHYL,-1	AQTLQQSR,-1	AVITVPAY,-1
AAICAGPT,-1	AEEKLQAS,-1	AGVIAGLN,-1	ALMKEEGV,-1	AQVLAQER,-1	AVLDGADC,-1
AAKDPSAV,-1	AEELMNFS,1	AHALAEGL,1	ALMLQGVD,-1	ARPPISMN,-1	AVLDQVEA,-1
AALCSEAA,-1	AEEVVQER,-1	AHAVDAVK,-1	ALNEANLS,-1	ASASFFLD,-1	AVLGHELG,-1
AALGGNSS,-1	AEEYKEAR,-1	AHEIGFGS,-1	ALNGKEVA,-1	ASDGIMVA,-1	AVLISEAQ,-1
AALIQQAT,-1	AEGIIHDT,-1	AHILSPWG,-1	ALNPDFKP,-1	ASEISTWD,-1	AVLQPTPQ,-1
AAMQDPEV,-1	AEGLGVIA,-1	AHPPSAEV,-1	ALSDHHIY,-1	ASFLNDVG,-1	AVLTVADM,-1
AANYQDTI,-1	AEHELNLA,-1	AHQVVVAK,-1	ALSDVCDV,-1	ASGKPIAA,-1	AVMNTWTK,-1
AAPAAAAA,-1	AEIEGAGA,-1	AIAAMNEP,-1	ALSLAVDK,-1	ASGMDEMA,-1	AVNAFQEA,1
AAPAKVEA,-1	AEIVQLVG,-1	AIAIGDLV,-1	ALTAASEA,-1	ASGVCIDS,-1	AVPVQDLG,-1
AAPLPMMP,-1	AEKDEFEH,-1	AIAPPLLQ,-1	ALVAEEHL,1	ASGVSVLP,-1	AVQNMVSH,-1
AAPVAAAT,-1	AEKLPNLT,-1	AIAPVLLD,-1	ALVDVIED,-1	ASIPLPDK,-1	AVSEKDIQ,-1
AAPVVPQA,-1	AELEQLKG,-1	AIATGGAV,-1	ALVHPDLD,-1	ASLDQPTQ,-1	AVVDLIDA,1
AASMSAFD,-1	AELVDSVL,-1	AICAGPTA,-1	ALYDICFR,-1	ASLEGDSG,-1	AVVILDAG,-1
AASQSFLQ,-1	AEMLLANL,-1	AIDEVVVT,1	ALYPEGQA,-1	ASLKPEFV,-1	AWAGAPLL,1
AASSSSLE,-1	AENTLQSF,-1	AIDGMNSI,-1	AMATVTAL,-1	ASMSAFDP,-1	AYAHELPK,-1
AATEDLWE,-1	AEPADFES,-1	AIEASEAA,1	AMEDGEID,1	ASNNELVR,-1	AYFNDSQR,-1
AATTAAPA,-1	AEQGRPPE,-1	AIEKPTYA,-1	AMFDQSQI,-1	ASPAHAVD,-1	AYVDLEKD,1
AAVAGGAP,-1	AEQLEQTK,-1	AIEPPPLD,-1	AMLGQAEE,-1	ASPLLQYF,-1	CAAYAHEL,-1
AAVMNTWT,-1	AESEKWQQ,-1	AIFNLLNL,-1	AMNYEGSP,-1	ASQEIAEK,-1	CADDRADL,-1
AAVPVQDL,-1	AESGVQHK,-1	AIGDLVKS,-1	AMQDPEVM,-1	ASQPGVDG,-1	CAGPTALL,-1
AAYAHELP,-1	AEVAQHYQ,-1	AILENANV,-1	AMVEYEID,1	ASQSFLQP,-1	CCENILLN,1
ACALLLLF,1	AEVKPLVE,-1	AILGITES,-1	ANALDNVD,-1	ASSQAVSN,-1	CEECIQLE,1
ACIGEKLD,-1	AEVPQAQP,-1	AILSAIAA,-1	ANAYHCEK,-1	ASSSSLEK,-1	CEVTNLND,-1
ADAVAVTM,-1	AEVVHPGD,-1	AILTQLHP,-1	ANDDIIVN,1	ASTCPDDE,-1	CFEAVVVT,1
ADDRADLA,-1	AFDGIIFQ,1	AIMENANV,-1	ANDEELNQ,-1	ASVADTAE,-1	CGIHETTF,-1
ADDVTLDD,-1	AFEAVVVT,1	AIQPGCIN,-1	ANILHDDC,-1	ASVLRISS,-1	CIGEKLDE,-1
ADEELLFN,1	AFGDVSKP,-1	AIQPNTTG,-1	ANKIGFEE,-1	ATAASSSS,-1	CIGENGEL,-1
ADELALVD,1	AFGSGGGL,-1	AIRPYVSR,-1	ANKVPAAA,-1	ATDFGEAL,-1	CILGQNGI,-1
ADESGQIF,-1	AFIQDPDG,-1	AISLFYEL,-1	ANSGAVKR,-1	ATDNQAIK,-1	CINYDLVK,-1
ADGAGLQL,-1	AFLADPSA,-1	AIVEALNG,-1	ANTLAEKD,-1	ATEDLWES,-1	CLEAYTGI,1
ADIEDFKA,-1	AFLHPEEF,-1	AIVLPMNH,-1	ANYQDTIG,-1	ATEMLVHN,1	CLEHGIQP,-1
ADILFNFS,1	AFNLFVGN,1	AIYDTPCI,-1	APAAAAAP,-1	ATGFIDGD,-1	CLELFSEL,1
ADIYTEHA,1	AFQDVAQN,-1	AIYKPVTD,-1	APAGITLK,-1	ATGGKYVP,-1	CLELFTEL,1
ADKFDENA,-1	AFVDFLSD,1	AKDDLSGA,-1	APAKVEAK,-1	ATHEDAVA,-1	CNDYVALV,1
ADKVIVTS,-1	AGAFVIVT,1	AKDPSAVA,-1	APESAPLK,-1	ATHEEAVA,-1	CNENSLFK,-1
ADLTGELM,-1	AGAGNSVL,-1	AKGDYPLE,-1	APGAGAIP,-1	ATILSMMD,1	CNLNELVK,-1
ADLVVGLC,-1	AGAVASYD,-1	ALAAVPVQ,-1	APLAAGIV,-1	ATMPSDVL,-1	CNPIISGL,-1
ADMLGSYG,-1	AGDALLQM,-1	ALAIVEAL,-1	APLLGWNS,-1	ATQGELVR,-1	CPELIEAF,1
ADPVSAQH,-1	AGDAPGEA,1	ALAQAVNA,-1	APLPMMPL,-1	ATQGHQDQ,-1	CPILPAKK,-1
ADSKPGTI,-1	AGDEESYE,-1	ALATLHDR,-1	APMFVMGV,-1	ATTAAPAA,-1	CSEAALQA,-1
ADSQPPVF,-1	AGDLAPIN,-1	ALCSEAAL,-1	APMHDLLL,-1	ATTLDESL,-1	CSQEESIK,-1
ADTHIPGS,-1	AGDQELLH,-1	ALDFEQEM,1	APPLLQEL,-1	ATVTTCHS,-1	CSSAPGPL,-1
ADTNNHQI,-1	AGEYAVHV,1	ALDLAVLD,1	APTQWLDG,-1	ATYDKLCK,-1	CTGDVTAW,-1

CVAGDEES,-1	DFTGTLIV,1	DLTGELMR,-1	DVDSLVIE,1	EDAAELVA,-1	EEYIQQLC,-1
CWELYCLE,1	DGAEAPAK,-1	DLTLYQSN,1	DVDSSVCR,-1	EDAVAAMA,-1	EFAIQPNT,-1
DAANFEQF,1	DGAGLQLR,-1	DLTNLIHT,-1	DVESYIHR,-1	EDAVAAMS,-1	EFALAIVE,1
DADIEDFK,-1	DGALNVDL,-1	DLVFIFWA,1	DVGDVDAK,-1	EDAYHEHQ,1	EFATHEDA,-1
DAEAILAA,-1	DGATILSM,-1	DLVGVLHD,1	DVGTGAGL,-1	EDDEVETA,1	EFATHEEA,-1
DAEDGPPE,-1	DGDGTITT,-1	DLVKSTLG,-1	DVIEDKLK,-1	EDDVGTGA,-1	EFDAVVVG,1
DAGAQYGK,-1	DGDLIESF,1	DLVVGLCT,-1	DVIQHETI,-1	EDELWASF,1	EFLTPVEE,-1
DAGKVDDA,-1	DGGDLTNL,-1	DMDQVENH,-1	DVIYIEAN,1	EDGPPELL,-1	EFPWGPKP,-1
DAIPAVEV,-1	DGHHHYEG,-1	DMEDLVHD,-1	DVLEVTKK,-1	EDGQEYAQ,-1	EFQTNLVP,-1
DAIQPGCI,-1	DGLNASQI,-1	DMLGSYGS,-1	DVLVSSGE,-1	EDHLAWSK,-1	EFSITYKP,-1
DALLFVHY,-1	DGLSNILK,-1	DMLLANKV,-1	DVPCGNIV,-1	EDIIDTGK,-1	EFVDIINA,-1
DALTDPSR,-1	DGMNSIAR,-1	DNAPPPEL,-1	DVQPHDLG,-1	EDIIQGFR,-1	EFVTHEDA,-1
DANTLAEK,-1	DGNKVTLD,-1	DNDSESDY,-1	DVTTPQTV,-1	EDKENYKK,-1	EGAELVDS,1
DATAQMAM,-1	DGQEYAQV,-1	DNEAIYDI,-1	DVVQAEAG,1	EDKGNQVY,-1	EGANAYHC,-1
DAVAAMAK,-1	DGQMPSDK,-1	DNEKDLVK,-1	DWCPTGFK,-1	EDKINALI,-1	EGDLIEHF,1
DAVAAMSK,-1	DGQQTIIA,-1	DNFGIVEG,-1	DWELMNDI,1	EDMEDLVH,-1	EGDMIVCA,1
DAVAVTMG,-1	DGSLDLNA,-1	DNPALADI,-1	DYDLIIIG,1	EDNADTLA,-1	EGDYVLFH,1
DAVIAELK,-1	DGSNVVFK,-1	DPAEKDEK,-1	DYGPHFQG,-1	EDNEKDLV,-1	EGEETFEA,-1
DAVTQQEK,-1	DGSQNTNV,-1	DPAGHQEE,-1	DYKPPATR,-1	EDPAGHQE,-1	EGEPGNKV,-1
DCAFLSAF,1	DGTIVDDD,-1	DPEGLEFP,1	DYPLEAVR,-1	EDPSTVLR,-1	EGETEANN,-1
DCAFMVDN,1	DGTVLCEL,-1	DPEVIPEN,-1	EAACLIVS,1	EDSEFTLA,-1	EGEVISLG,-1
DCIMLSGE,1	DGVADVSI,-1	DPLGGSDT,-1	EAAEQGRP,-1	EDSSGNVV,-1	EGGVDVGD,-1
DDAEAILA,-1	DGVANALD,-1	DPNLSELR,-1	EAALQAIR,-1	EDTFIADL,1	EGHLGVTK,-1
DDDDEEAE,1	DGVSIPGE,-1	DPQPEHPL,-1	EAANYQDT,-1	EDTNLCAI,-1	EGIFTGSA,-1
DDDETEVL,-1	DGYWIEIL,1	DPTGTYHG,-1	EAASLLGK,-1	EDVPCGNI,-1	EGIIHDTE,-1
DDDFFQNL,1	DHFLFDKP,1	DPVSAQHA,-1	EACDVCLK,-1	EDVQPHDL,-1	EGLGVIAC,-1
DDDYFLCL,1	DIAMTELP,1	DQDILDLV,-1	EADGAGLQ,-1	EEAEEKAP,-1	EGLTLNLE,-1
DDEEIELA,1	DIAVDGEP,-1	DQDLITII,1	EAEADGAG,-1	EEAENTLQ,-1	EGNDLYHE,-1
DDGGDLTN,-1	DICFTSVQ,-1	DQELLHQA,-1	EAEEKAPV,-1	EEAIRPYV,-1	EGNEQFIN,-1
DDGVSIPG,-1	DIEDFKAK,-1	DQLHEGAK,-1	EAEMIQLQ,1	EEALHYLT,-1	EGQAPVKK,-1
DDHDPVDK,-1	DIELVSNS,1	DQLKPGGR,-1	EAENTLQS,-1	EEAVAAMS,-1	EGTLLKPN,-1
DDHDSVDK,-1	DIENAYKK,-1	DQLQGGQL,-1	EAFDISKK,-1	EEDAAELV,-1	EGVAHIID,-1
DDIGHGVK,-1	DIKPIWQR,-1	DQPTQTVV,-1	EAFLNSQK,-1	EEDLVVWV,1	EHAHQVVV,-1
DDLDFFIG,-1	DIPAAVNA,-1	DQQSFGIK,-1	EAGGMHTV,-1	EEDSDEEE,-1	EHCSSIVM,-1
DDLSGADI,-1	DKATYDKL,-1	DQSQIQEF,-1	EAGKDDYV,-1	EEEAIQLD,1	EHELNLAR,-1
DDRADLAK,-1	DKDGDGTI,-1	DQVENHEF,-1	EAHGTGTK,-1	EEEDLAEI,1	EHFSQFGT,-1
DDVDSSVC,-1	DKFDENAK,-1	DQVIQSLI,-1	EAIAPPLL,-1	EEEDSDEE,-1	EHGDSSVP,-1
DDVFLSVP,1	DKGNQVYR,-1	DSDLQLER,-1	EAIEKPTY,-1	EEEEDSDE,-1	EHGIDPTG,-1
DDVGTGAG,-1	DKINALIK,-1	DSDMMLNI,1	EAINVEQA,1	EEELLVIF,1	EHGIQPDG,-1
DEAIEKPT,-1	DKPVSPLL,-1	DSEFFLTT,1	EAIRPYVS,-1	EEEQAFKR,-1	EHLKNPVI,-1
DECGAGVF,-1	DKVIVTSK,-1	DSEFTLAS,-1	EAIVLPMN,-1	EEGETEAN,-1	EHLTVDAR,-1
DEEEVELC,1	DLDALAAE,1	DSKPGTIR,-1	EAIYDICR,-1	EEGLTLNL,-1	EHPDKFLK,-1
DEELNQLL,-1	DLDLTADS,1	DSLAANVK,-1	EALGIAQP,-1	EEHLTVDA,-1	EHQANLLR,-1
DEEMDLAI,-1	DLEEAEEY,1	DSPSAQLA,-1	EALGTLMR,-1	EEIAVQFV,1	EHSLVIDT,-1
DEEMLFIY,1	DLEGENIE,-1	DSQPPVFK,-1	EALHYLTK,-1	EEIEIVLA,1	EHSMDTLL,-1
DEESEQAT,-1	DLELINVC,1	DSSGNVVN,-1	EALKDDSW,-1	EEKAPVKK,-1	EHSVNYKP,-1
DEESYEVF,-1	DLENLPAS,-1	DSSVPVWS,-1	EALNGKEV,-1	EEKLQASV,-1	EHVAAAEL,-1
DEHGIDPT,-1	DLEPTVID,-1	DSVLDVVR,-1	EALYDICF,-1	EELAAAER,-1	EIDGNKVT,-1
DEHSVNYK,-1	DLGGSSFR,-1	DSVYLSEV,1	EAMNYEGS,-1	EELNQLLK,-1	EIDLQKMP,-1
DEILEASD,1	DLGGTNFR,-1	DTHIPGSP,-1	EANKIGFE,-1	EEMDLAIE,-1	EIEGAGAA,-1
DESGPSIV,-1	DLGSTVIK,-1	DTLALVFE,1	EANLSNLK,-1	EEMGHCDS,-1	EIEGEEIE,-1
DETIKNPR,-1	DLLLWNNA,1	DTLLATLK,-1	EANSGAVK,-1	EENFAVEA,1	EIENNPTV,-1
DEVLVNHF,1	DLLSNITL,1	DTNLCAIH,-1	EAPLAAGI,-1	EENLDCPE,-1	EIGRPLGK,-1
DEYCVQQL,-1	DLMTQAGV,-1	DTNLVALM,1	EASDGIMV,-1	EENLMDAQ,-1	EIIEGMKF,-1
DFAAEVVH,-1	DLNAAESG,1	DTNNHQIK,-1	EASLEGDS,-1	EEQQMHLN,-1	EIINEDIA,-1
DFALVQRP,1	DLNEVPVK,-1	DTPTQEDW,-1	EASLESAK,-1	EEQTVDGR,-1	EILQEEED,-1
DFEPSTES,-1	DLNFIKQS,-1	DTQVVVFK,-1	EATDNQAI,-1	EESEQATE,-1	EIRDMLLA,-1
DFESLLLS,1	DLQKMPLG,-1	DTSRPPLE,-1	EATGGKYV,-1	EESQVAGT,-1	EISEDKTK,-1
DFGEALVR,-1	DLRPFLAK,-1	DVAPLSLG,-1	EAVAAMSK,-1	EESYEVFK,-1	EISFGTTK,-1
DFKPPADY,-1	DLSGADIK,-1	DVAQNPAN,-1	EAVQNMVS,-1	EEVDEEGK,1	EITYVELQ,1
DFLSDEIK,-1	DLSLAVLQ,1	DVCDVPTA,-1	ECADDRAD,-1	EEVDFAGW,1	EIVQLVGK,-1
DFMGITLA,1	DLSNGKPR,-1	DVDHQIAK,-1	ECFDEITY,-1	EEVGVEHL,-1	EKDEFEHK,-1
DFQLIGIQ,-1	DLTGDLES,-1	DVDLAEVK,1	ECNLNELV,-1	EEVLAVLG,1	EKDFAAEV,-1

EKDIQDLK,-1	EPGDYNIN,-1	EVGDTQVV,-1	FESLPENA,-1	FPMTHGNT,-1	GDDLTVTN,1
EKEQIVPK,-1	EPGNKVNL,-1	EVGVEHLL,-1	FETLKPST,-1	FPSEIVGK,-1	GDEESYEV,-1
EKIEKEQS,-1	EPIDEYCV,-1	EVIPENTD,-1	FEVPFAPS,-1	FPWGPKPF,-1	GDEMTSLS,1
EKLPNLTH,-1	EPTTAFNL,-1	EVISDEHG,1	FFGTHETA,-1	FQALLQYA,1	GDEMVSLK,1
EKLQASVR,-1	EPTVIDEV,-1	EVISLGLP,-1	FFIGDEAI,1	FQDGDLTL,-1	GDEYFTFI,1
EKPTYATK,-1	EQAFQTIA,-1	EVISWLDA,1	FGDFVALS,1	FQDVAQNP,-1	GDGAEAPA,-1
EKQQQDQV,-1	EQEMATAA,-1	EVIYMIEP,1	FGDVSKPE,-1	FQEAASLL,-1	GDGTITTK,-1
EKWQQALK,-1	EQFINAAK,-1	EVKPLVEK,-1	FGEEGLTL,-1	FQLIGIQD,-1	GDHCIIGR,-1
ELAEDKEN,-1	EQGETPLT,-1	EVMNSFEL,-1	FGSGGGLL,-1	FQNLDGVA,-1	GDIPAAVN,-1
ELASQPGV,-1	EQGHLLPE,-1	EVNLLVEE,1	FGTHETAF,-1	FQSSANYA,-1	GDLAPINA,-1
ELAYEQVA,-1	EQGRPPEH,-1	EVPFAPSG,-1	FGVGFYSA,-1	FQTLLDAG,-1	GDLESFKK,-1
ELCPILPA,-1	EQILPTLV,-1	EVPQAQPV,-1	FHHEGGVD,-1	FQTNLVPY,-1	GDLTNLIH,-1
ELDAFLKE,1	EQIVPKPE,-1	EVTHQFVK,-1	FHSYFMEA,1	FRDGDILG,-1	GDLVKSTL,-1
ELDDLLVD,1	EQLEQTKR,-1	EVTKENLL,-1	FIENNVSK,-1	FRPDNFVF,-1	GDQELLHQ,-1
ELDLSLSD,1	EQLKGQGK,-1	EVTNLNDY,-1	FIGGLAAQ,-1	FSDNAPPP,-1	GDQQSFGI,-1
ELEAVQNM,1	EQLLQDPK,-1	EVVDKDSI,-1	FIHGGHTA,-1	FSELAEDK,-1	GDSDLQLE,-1
ELEIIEGM,1	EQQMHLNV,-1	EVVHPGDL,-1	FILVGPEG,-1	FSGSSHQD,-1	GDSSVPVW,-1
ELEMIGFI,1	EQQQAYRP,-1	EVVITHLA,-1	FIQDPDGY,-1	FSITYKPV,-1	GDTQVVVF,-1
ELEQLKGQ,-1	EQTVDGRP,-1	EVVTESEK,-1	FIQSIIST,-1	FSKGEENL,-1	GDVAEGDL,-1
ELGLVDGQ,-1	ERPFLAIL,-1	EVVYMTEP,1	FISSGLSK,-1	FSPDGHLF,-1	GDVSKPER,-1
ELIKVEGK,-1	ESCGIHET,-1	EWCDGCEA,-1	FITDCKDP,-1	FSPHTKPW,-1	GDVTAWTK,-1
ELINALYP,1	ESDYMFMA,1	EWIPNNVK,-1	FKDDYFAK,-1	FSQFGTVE,-1	GDYNINIL,-1
ELINGYIQ,-1	ESEKWQQA,-1	EYALAAVA,1	FKEEEPVK,-1	FTDVNSIL,-1	GDYPLEAV,-1
ELIPQLVA,-1	ESEMEDAY,-1	EYAQEAVK,-1	FKPPADYK,-1	FTELAEDK,-1	GEADVEFA,1
ELLDYEDD,-1	ESENKIPA,-1	EYASPAHA,-1	FLADPSAF,-1	FTFITDCK,-1	GEADVEFV,1
ELLPEAQH,-1	ESEQATEM,-1	EYCVQQLK,-1	FLAHILSP,-1	FTGSADIK,-1	GEAEADGA,-1
ELNGNQPM,-1	ESFLDISR,-1	EYEVVTES,-1	FLAILGGA,-1	FVDIINAK,-1	GEAFVQFA,1
ELNQYSAK,-1	ESGELNQY,-1	EYIQQLCE,-1	FLAPISSS,-1	FVDWCPTG,-1	GEEDEEAL,1
ELNVPFFK,-1	ESGPSIVH,-1	EYTFIQNV,1	FLAQTEAL,-1	FVESDADE,-1	GEEFEEQT,1
ELPPTHPI,-1	ESGVINLK,-1	FAAEVVHP,-1	FLCLPSNT,-1	FVGNLNFN,-1	GEEGLTLN,-1
ELQKEEAQ,-1	ESGVQHKP,-1	FADTHIPG,-1	FLDALIVS,1	FVGSGVSG,-1	GEENLMDA,-1
ELSLLGKK,-1	ESHQDADI,-1	FAIQPNTT,-1	FLDISRPK,-1	FVGSPVED,-1	GEFSITYK,-1
ELVALAQA,1	ESHQFQPK,-1	FAIYKPVT,-1	FLDLTVQQ,1	FVGSQATD,-1	GEHGDSSV,-1
ELVDSVLD,-1	ESLLPGIR,-1	FAKPPDQK,-1	FLFCSEYR,-1	FVITKPDV,-1	GEHTPSAL,-1
ELVFKEDG,1	ESLPENAS,-1	FAPVNVTT,-1	FLFRDGDI,-1	FVLDEADV,-1	GEIDGNKV,-1
ELYCLEHG,1	ESQVAGTP,-1	FAQTLQQS,-1	FLHELNVP,-1	FVMGVNHE,-1	GELLYVAD,1
EMADFLSD,1	ESSSPEVK,-1	FASQEIAE,-1	FLHPEEFE,-1	FVSFSEVQ,-1	GELNQYSA,-1
EMATAASS,-1	ESTGVFTT,-1	FASTCPDD,-1	FLKEPALN,-1	FVTHEDAV,-1	GEPGNKVN,-1
EMDGTENK,-1	ESTPPSEE,-1	FATHEDAV,-1	FLNDVGPK,-1	FVTLAPTQ,-1	GERPVVQR,-1
EMDLAIEA,-1	ETAAGGDG,-1	FATHEEAV,-1	FLPFADDK,-1	FWAPESAP,-1	GETAKGDY,-1
EMELNEHS,1	ETAFLGPK,-1	FCLSEDKK,-1	FLQAAKDP,-1	FYELSEND,1	GETEANNH,-1
EMGHCDSV,-1	ETAGGVMT,-1	FCSEYRPK,-1	FLQALSLA,-1	GADDIELL,1	GETEDTFI,-1
EMILTEKE,1	ETAKGDYP,-1	FDDHDPVD,-1	FLQDVSAS,-1	GADFLVTE,1	GETEDTFM,-1
EMLLANLR,-1	ETDLLILF,1	FDDHDSVD,-1	FLQLVDAG,-1	GADLAALC,1	GETNPADS,-1
ENANVLAR,-1	ETEANNHK,-1	FDGALNVD,-1	FLQSHENQ,-1	GAEAPAKK,-1	GEVISLGL,-1
ENASGKPI,-1	ETEDTFIA,-1	FDIAVDGE,-1	FLSDAIPG,-1	GAELVDSV,1	GFAFVTFD,1
ENDLNFIK,-1	ETEDTFMA,-1	FDKATYDK,-1	FLSDEIKE,-1	GAEYVVES,1	GFAGLVQE,1
ENEEDEHS,1	ETGKITDF,-1	FDKDGDGT,-1	FLSTSIAQ,-1	GAGFQDIP,-1	GFEFTLMV,1
ENGGSLGS,-1	ETGVLKPG,-1	FDKPVSPL,-1	FLTDLYPE,-1	GAGGAGLR,-1	GFGAQGPK,-1
ENIEIVFA,1	ETHGHVGA,-1	FDPNLSEL,-1	FLTPVEEA,-1	GAGGPGPG,-1	GFGFVTFD,1
ENILHVSE,-1	ETIGEILK,-1	FDQLHEGA,-1	FLTTASGV,-1	GAGLLEIK,-1	GFIENNVS,-1
ENKIPATQ,-1	ETLKPSTL,-1	FDQSQIQE,-1	FLVGGASL,-1	GAGNNWAK,-1	GFQLVFLP,1
ENLDCPEL,-1	ETLPTEYD,-1	FEALGIAQ,-1	FMADLVVG,1	GANAYHCE,-1	GFSGIESS,1
ENLLDFIK,-1	ETNPADSK,-1	FEAPNQEK,-1	FMAPGAGA,-1	GAPMHDLL,-1	GGAPSVGI,-1
ENLMDAQV,-1	ETPTGYIE,-1	FEATDNQA,-1	FMEAIAPP,-1	GAPSVGIK,-1	GGASLKPE,-1
ENPEHNDF,-1	ETTFNSIM,-1	FEDTNLCA,-1	FMYLNEVA,-1	GASLKPEF,-1	GGAVFGEE,-1
ENQEIYQK,-1	ETTQAMAF,-1	FEGEPGNK,-1	FNCEVTNL,-1	GAVALLIG,1	GGDGAEAP,-1
ENSEGGLH,-1	ETVAESAE,-1	FEIGHFFR,-1	FNIPFTGN,-1	GAVASYDY,-1	GGDLTNLI,-1
ENTDLVTL,1	EVAQHYQD,-1	FEIGHFLR,-1	FNKSAPEL,-1	GAVFGEEG,1	GGFGAQGP,-1
ENTLQSFR,-1	EVDDAETL,1	FEIGRPLG,-1	FNSIATQG,-1	GCEAVLLG,1	GGGCALLR,-1
ENVRPDIV,-1	EVEAIALA,1	FELIKVEG,-1	FNTDEDTK,-1	GCINYDLV,-1	GGGSGGLA,-1
EPALNEAN,-1	EVELVQMV,1	FEQEMATA,-1	FPIPEEYT,-1	GCVAGDEE,-1	GGLAAQEV,-1
EPASLLEL,-1	EVENGGSL,-1	FEQFLQER,-1	FPLTAQHE,-1	GDAIPAVE,-1	GGMAFHSY,-1

GGMHTVCL,-1	GPGGFGAQ,-1	GVSLVEQL,1	HQRPPSEM,-1	IEGMKFDR,-1	IISTVEPA,-1
GGNSSPSA,-1	GPGLSQEA,-1	GVSVLPQN,-1	HSENFQTL,1	IEHFSQFG,-1	IIVNWVNR,-1
GGPGPGGF,-1	GPGPGGFG,-1	GVTGGWDN,-1	HSLVIDTL,-1	IEHIQVNK,-1	IKPIWQRP,-1
GGSGGLAA,-1	GPGTSFEF,-1	GVVEVENP,1	HSMDTLLA,-1	IEIDFETL,1	ILDAGAQY,-1
GGSGGLAS,-1	GPLGECII,-1	GVVIAADM,-1	HSQAVEEL,-1	IEILNPNK,-1	ILDDGGDL,-1
GGSLGSKK,-1	GPNAPLIF,-1	GWNSFVVW,-1	HSSLAEPR,-1	IEKPTYAT,-1	ILDDIGHG,-1
GGSSTPLI,-1	GQAEEVVQ,-1	GWQFVNLG,1	HSSVYPTQ,-1	IELAYEQV,-1	ILDDVDSS,-1
GGTTMYPG,-1	GQELAVAD,1	GWVLGEHG,1	HSVNYKPP,-1	IELLPEAQ,-1	ILEASLEG,-1
GGVDVGDV,-1	GQEYAQVI,-1	GYDYGPHF,-1	HTECCHGD,-1	IEMDGTEN,-1	ILEDLVFP,1
GGVMTALI,-1	GQGEVLVY,-1	GYEFDICF,1	HTSQSGDE,-1	IENNPTVK,-1	ILEGHLGV,-1
GGVTGGWD,-1	GQIFICSQ,1	GYIESLPR,-1	HVAAAELG,-1	IENTPEEK,-1	ILEGNDIE,-1
GHELGHWK,-1	GQNGISDL,-1	GYIFLEYA,1	HVLCNSED,-1	IEPIDEYC,-1	ILENANVL,-1
GHLLPERK,-1	GQQTIIAC,-1	GYPVTNAV,-1	HVSFPLEG,-1	IEPPPLDA,-1	ILETLPTE,-1
GHQDQSIR,-1	GQSGAGNN,-1	HAHQVVVA,-1	HYEGAVVI,1	IESFLDIS,-1	ILFKDDYF,-1
GHVFEESQ,1	GRPPEHTS,-1	HAITATQK,-1	HYLAVVLL,1	IESGVINL,-1	ILGITESF,-1
GHYLGEFS,1	GSEVVDKK,-1	HDDEVTVT,1	HYQDTLIR,-1	IESHQFQP,-1	ILGQNGIS,-1
GHYTEGAE,-1	GSGGGLLQ,-1	HDNFGIVE,-1	HYTEGAEL,-1	IESSSPEV,-1	ILHDDCAF,-1
GIDPTGTY,-1	GSGGLAAA,-1	HDPETLLR,-1	IAADMLGS,-1	IETIGEIL,-1	ILISSEEG,1
GIDVQQVS,-1	GSGGLASA,-1	HEDAVAAM,-1	IAAMNEPK,-1	IEVMNSFE,-1	ILLDQGQL,-1
GIEAAEML,1	GSGVSGGE,-1	HEEAVAAM,-1	IAASQSFL,-1	IFDHEFTK,-1	ILLNAAWL,-1
GIEETILE,1	GSLLWAAG,-1	HEEFSEHA,1	IAAVMNTW,-1	IFESLPEN,-1	ILNKPGLK,-1
GIEQQVLR,-1	GSPVEDNE,-1	HEELMLGD,1	IACIGEKL,-1	IFQGQSLK,-1	ILPVGAAN,-1
GIFTGSAD,-1	GSQATDFG,-1	HEGGVDVG,-1	IADLVVGL,-1	IFTDVNSI,-1	ILPVGAES,-1
GIHETTFN,-1	GSQFFVTL,1	HEHQANLL,-1	IAFGSGGG,-1	IFTGSADI,-1	ILQAEHEL,-1
GIIHDTET,-1	GSQNTNVD,-1	HEIGFGSK,-1	IAGLNVLR,-1	IFWAPESA,-1	ILSAIAAM,-1
GIMDHEEA,-1	GSQQADFL,-1	HELNVPFF,-1	IAIGDLVK,-1	IGDLVKST,-1	ILSMMDVD,1
GIQPDGQM,-1	GSSHQDLS,-1	HENQEIYQ,-1	IALATLHD,-1	IGDMAILG,1	ILSPWGAE,-1
GISAIDGM,-1	GSSTPLIP,-1	HESGELNQ,-1	IALDLGGS,-1	IGEHTPSA,-1	ILTLLQGV,1
GITESFQV,-1	GSVPAPAC,-1	HETAFLGP,-1	IAMATVTA,-1	IGEKLDER,-1	ILTQLHPR,-1
GITKPAIR,-1	GSYGSLAR,-1	HETTFNSI,-1	IAPPLLQE,-1	IGENGELL,-1	ILVGDLMR,-1
GKEVAAQV,-1	GTEPTTAF,-1	HEVNLLVE,1	IAPVLLDA,-1	IGFIENNV,-1	ILVGPEGC,-1
GKITDFIK,-1	GTGAGLLE,-1	HEWCDGCE,-1	IASVADTA,-1	IGGGSGGL,-1	IMAKDDLS,-1
GKPIAAVM,-1	GTHETAFL,-1	HFCEEFGK,-1	IATGGAVF,-1	IGGLAAQE,-1	IMDHEEAR,-1
GLAAQEVM,-1	GTIVDDDD,-1	HFPMTHGN,-1	IATQGELV,-1	IGHVDSGK,-1	IMENANVL,-1
GLAGAGNS,-1	GTLLKPNM,-1	HFQGILEA,1	IATQGHQD,-1	IGKEDAVR,-1	INALYPEG,-1
GLCTGQIK,-1	GTPMFVVK,-1	HFSQFGTV,-1	IAVDGEPL,-1	IGPNAPLI,-1	INEDIAKR,-1
GLDIPEVD,-1	GTSFEFAL,-1	HGDLLECA,1	IAVNAVLT,1	IHDNFGIV,-1	INILFADT,1
GLDVPQVS,-1	GTTHQTSL,-1	HGDSDLQL,-1	ICAGPTAL,-1	IHETTFNS,-1	INSSITTK,-1
GLESIISI,1	GTTMYPGI,-1	HGDSSVPV,-1	ICDYELHE,1	IHGGHTAK,-1	INTACFEA,-1
GLGVIACI,-1	GTVLCELI,-1	HGHVGADL,-1	ICFTSVQK,-1	IHLPSPVT,-1	INVCNENS,-1
GLLDSPAL,-1	GTVLTEHV,1	HGIDPTGT,-1	ICSQEESI,-1	IHNGIITN,-1	INYDLPNN,-1
GLNASQIR,-1	GTYHGDSD,-1	HGIQPDGQ,-1	IDAIQPGC,-1	IHSSLAEP,-1	INYDLPTN,-1
GLSQEAAR,-1	GVADLTGE,-1	HGNTGFSG,-1	IDDKGILR,-1	IIACIESH,1	IPFTGNVK,-1
GLTAFEAT,1	GVADVSIE,-1	HHEGGVDV,-1	IDEYCVQQ,-1	IIAGHESG,1	IPGGSSTP,-1
GLTLGGQK,-1	GVAHIIDP,-1	HHIYLEGT,1	IDGMNSIA,-1	IIDDKGIL,-1	IPVDISDS,-1
GLTLNLED,-1	GVANALDN,-1	HILSPWGA,-1	IDGNKVTL,-1	IIDIITHR,-1	IPVLVGAL,-1
GLVDGQEL,-1	GVAPESFE,-1	HIPGSPFK,-1	IDGQQTII,-1	IIDILTKR,-1	IPVQTQHP,-1
GMDEMAVE,-1	GVCIDSEF,-1	HIVEAEAM,1	IDGTHIAK,-1	IIEACDVC,-1	IQAMPTLI,-1
GMHTVCLS,-1	GVDDLDFF,-1	HKPSAPQG,-1	IDLQKMPL,-1	IIEGMKFD,-1	IQDGSLDL,-1
GMLDPAEK,-1	GVDGFLVG,1	HLFQVEYA,1	IDNPALAD,-1	IIENTPEE,-1	IQDGSQNT,-1
GMNVAGVS,-1	GVDLLADA,1	HLKNPVIA,-1	IDNSQGAY,-1	IIFQGQSL,-1	IQDGYLSL,1
GMYGIENE,-1	GVDQFLVK,-1	HLNLSGNK,-1	IDPTGTYH,-1	IIGGGSGG,-1	IQDPDGYW,-1
GNACWELY,-1	GVDVGDVD,-1	HLPSPVTA,-1	IDQLKPGG,-1	IIGKEDAV,-1	IQDTSRPP,-1
GNDLYHEM,-1	GVEELESE,1	HMHAYALE,-1	IEACDVCL,-1	IIHDTETK,-1	IQELVEAI,1
GNEQFINA,-1	GVFTTMEK,-1	HNAQNLMQ,-1	IEAEHTLR,-1	IIHSSLAE,-1	IQGITKPA,-1
GNKVNLAE,-1	GVGFYSAF,-1	HNGIITNY,-1	IEAFLNSQ,-1	IIIGGGSG,-1	IQHETIGK,-1
GNKVTLDW,-1	GVHQGAGF,-1	HPDFEPST,-1	IEAHGTGT,-1	IINEDIAK,-1	IQIDLETG,1
GNLNFNKS,-1	GVIACIGE,-1	HPEEFEHM,-1	IEANSGAV,-1	IINSSITT,-1	IQLDGLNA,-1
GNQPMEIK,-1	GVIAGLNV,-1	HPELFEAL,1	IEDPSTVL,-1	IINYDLPN,-1	IQLEPTFI,-1
GNSSPSAK,-1	GVMTALIK,-1	HPLSCHGW,-1	IEELFLDL,1	IIPVLVGA,-1	IQLKPDVV,-1
GNVTTCND,-1	GVQHKPSA,-1	HPPSAEVE,-1	IEENKYSR,-1	IIQLKPDV,-1	IQLQEELA,-1
GPDTNGSQ,-1	GVSGGEEG,-1	HQDADIED,-1	IEESAIDE,-1	IISAPSAD,-1	IQNVPLED,-1
GPEAPLAA,-1	GVSIPGEY,-1	HQGAGFQD,-1	IEGAGAAK,-1	IISIIHSS,-1	IQPDGQMP,-1

IQPGCINY,-1	IYDTPCIQ,-1	LAGAGNSV,-1	LEDVQPHD,-1	LGWNSFVV,-1	LLLGQTDD,-1
IQPNTTGK,-1	IYIPLPDE,-1	LAHEIGFG,-1	LEFAIQPN,-1	LGYPVTNA,-1	LLLSRPVL,-1
IQQATTVK,-1	IYKPVTDF,-1	LAHILSPW,-1	LEFPWGPK,-1	LHDPETLL,-1	LLNAAWLK,-1
IQSIISTV,-1	IYTNYEAG,1	LAIFNLLN,-1	LEGANAYH,-1	LHELNVPF,-1	LLNLCYLD,1
IQSSPPKD,-1	IYVPLTDK,-1	LAILGGAK,-1	LEGEETFE,-1	LHEWCDGC,-1	LLPAQTSL,-1
IQTGISAI,-1	KATYDKLC,-1	LAILLGML,1	LEGHLGVT,-1	LHPEEFEH,-1	LLPEAQHK,-1
IQVTPPGF,-1	KDDLSGAD,-1	LAIVEALN,-1	LEGIFTGS,-1	LHVDLAQI,1	LLQDSGEV,-1
IRDMLLAN,-1	KDDYFAKK,-1	LALDLGGT,-1	LEGLDLRP,1	LIALSIDS,-1	LLQMITIH,1
ISADIETI,1	KDFAAEVV,-1	LALLDGSN,-1	LEGNDIEL,-1	LIDAIQPG,-1	LLSGLLDS,-1
ISAIDGMN,-1	KDGDGTIT,-1	LANKVPAA,-1	LEGNEQFI,-1	LIFESLPE,-1	LLSRPVLE,-1
ISASASFF,-1	KDPSAVAK,-1	LAPISSSK,-1	LEGSGLES,-1	LIGPNAPL,-1	LLSYVHIL,-1
ISEAQAIK,-1	KDPSVVVR,-1	LAPTQWLD,-1	LEGTLLKP,-1	LIINYDLP,-1	LLVAETFA,-1
ISEGLHPR,-1	KDVESYIH,-1	LAQAVNAR,-1	LEHGIQPD,-1	LILEGNDI,-1	LLVDLDHQ,-1
ISFGTTKD,-1	KEDGQEYA,-1	LAQIAAEN,-1	LEHSDCAF,-1	LILETLPT,-1	LLVEEIHR,-1
ISFTEVDD,-1	KEDIIQGF,-1	LAQIIEAC,-1	LEIINEDI,-1	LILFKDDY,-1	LLWAAGQR,-1
ISGLYQGA,1	KEEEPVKK,-1	LAQTEALM,-1	LEKDFAAE,-1	LIMAKDDL,-1	LLWSQGQL,-1
ISIIHSSL,-1	KENLLDFI,-1	LASDFEIG,1	LEKIEKEQ,-1	LINAAIQK,-1	LLYDLENL,-1
ISLGLPFG,-1	KEPALNEA,-1	LASEISTW,-1	LELFTELA,1	LINGYIQK,-1	LLYRGDVV,-1
ISLSGDHC,-1	KEQIVPKP,-1	LASQPGVD,-1	LELINGYI,-1	LIPVQTQH,-1	LMEYAILS,1
ISMNFEVP,1	KEVAAQVK,-1	LASSQAVS,-1	LENANVLA,-1	LIQQATTV,-1	LMGAVVME,1
ISSSLAVV,-1	KEVSTYIK,-1	LATTLDES,-1	LENASGKP,-1	LISEAQAI,-1	LMKDPSVV,-1
ISTVEPAQ,-1	KGDYPLEA,-1	LAVANDEE,-1	LENLPASK,-1	LISFTEVD,-1	LMKEEGVK,-1
ISVEGSSK,-1	KGEENLMD,-1	LAVDAVIA,-1	LEPTVIDE,-1	LIVSMDVI,-1	LMLQGVDL,-1
ITDCKDPK,-1	KIEKEQSK,-1	LAVDYENV,1	LEQFVSIL,-1	LIVSVDET,-1	LMTQAGVE,-1
ITEGFEAA,1	KLPNLTHL,-1	LAVIPGGS,-1	LEQLKGQG,-1	LIVVPDVS,-1	LMTTVHAI,-1
ITEMVALN,1	KNPVIAQK,-1	LAYEQVAK,-1	LESEMEDA,-1	LIWNIKDE,-1	LMVVGESG,-1
ITESFQVK,-1	KPDVVITE,-1	LCEDIIQL,1	LESENKIP,-1	LIYIPLPD,-1	LNDDDETE,-1
ITFKEEEP,-1	KPEEEVAQ,-1	LCELINAL,1	LESHQDAD,-1	LKDDSWLK,-1	LNEANLSN,-1
ITHLAPGT,-1	KPEFVDII,-1	LCIGENGE,-1	LESLLPGI,-1	LKEPALNE,-1	LNFIKQSK,-1
ITIHLPSP,-1	KPEMIGHY,-1	LCLPSNTK,-1	LETAGGVM,-1	LKNPVIAQ,-1	LNFNKSAP,-1
ITIIGKED,-1	KPGMVVTF,1	LCNSEDIR,-1	LETGKITD,-1	LKPDVVIT,-1	LNGKEVAA,-1
ITKLVEVG,-1	KPIAAVMN,-1	LCPILPAK,-1	LETLPTEY,-1	LKPEFVDI,-1	LNGNLQLE,-1
ITKPDVYK,-1	KPISVEGS,-1	LCSEAALQ,-1	LEYASPAH,-1	LKPISVEG,-1	LNGNQPME,-1
ITLASSQA,-1	KPIWQRPS,-1	LCYVALDF,-1	LFCSEYRP,-1	LKPNMVTP,-1	LNIINSSI,-1
ITVPAYFN,-1	KPNMVTPG,-1	LDAGAQYG,-1	LFDKDGDG,-1	LLAALGGN,-1	LNLEDVQP,-1
IVAASNLR,-1	KPPADYKP,-1	LDAGLPQK,-1	LFDQLHEG,-1	LLAEPADF,-1	LNLQDESF,-1
IVCAAYAH,-1	KPSAPQGG,-1	LDALTDPS,-1	LFHHEGGV,-1	LLANFLAQ,1	LNPDFKPP,-1
IVDVIDQN,-1	KPSFDPNL,-1	LDANTLAE,-1	LFIYGHYK,-1	LLANKVPA,-1	LNSLAEEK,-1
IVEALNGK,-1	KPVSPLLL,-1	LDAVTQQE,-1	LFKDDYFA,-1	LLASGMAR,-1	LNSNGFIC,-1
IVEDIIDT,-1	KQQQDQVD,-1	LDDGGDLT,-1	LFLTDLYP,-1	LLDAGLPQ,-1	LNSNTQVV,-1
IVEGLMTT,1	KVIVVITD,-1	LDDIGHGV,-1	LFNIPFTG,-1	LLDALTDP,-1	LPAGDALL,-1
IVFAKPPD,-1	KVNLAELF,-1	LDDVDSSV,-1	LFPLTAQH,-1	LLDAVTQQ,-1	LPAQTSLE,-1
IVIHNGII,-1	KVTLDWAK,-1	LDESLTNR,-1	LFRDGDIL,-1	LLDGSNVV,-1	LPCILNAR,-1
IVKEVSTY,-1	LAADFEIG,1	LDGADCIM,-1	LGDQQSFG,-1	LLDQGQLN,-1	LPEDPLLS,1
IVLAKPPD,-1	LAAEIEGA,-1	LDGLNASQ,-1	LGECIIAG,-1	LLDSPALK,-1	LPENASHK,-1
IVLGGGCA,-1	LAAGIVGN,-1	LDGLSNIL,-1	LGFSEVEL,-1	LLDVAPLS,-1	LPFADDKR,-1
IVLPMNHK,-1	LAALGGNS,-1	LDGNELDL,-1	LGGGCALL,-1	LLDYEDDE,-1	LPGAAVDL,-1
IVLTTSAG,-1	LAAMQDPE,-1	LDGSNVVF,-1	LGGNSSPS,-1	LLEGSGLE,-1	LPNLTHLN,-1
IVMDPLGG,-1	LAAQEVMK,-1	LDGVANAL,-1	LGGSDTAK,-1	LLELFNSI,1	LPPTHPIR,-1
IVNNLLKP,-1	LAAVPVQD,-1	LDLGGSSF,-1	LGGSLMEY,-1	LLENLEKI,1	LPSPVTAQ,-1
IVNTNVPR,-1	LADAVAVT,-1	LDLGGTNF,-1	LGHELGHW,-1	LLFNIPFT,-1	LPTEYDSR,-1
IVPKPEEE,-1	LADDVTLD,-1	LDLSNGKP,-1	LGIEQQVL,-1	LLFPLTAQ,-1	LPVGAANF,-1
IVRPDNTY,-1	LAEALGTL,-1	LDNGAQID,1	LGITESFQ,-1	LLFVHYLA,-1	LPVGAESF,-1
IVSMDVIQ,-1	LAEDKENY,-1	LDPAEKDE,-1	LGLVDGQE,-1	LLGGSLME,-1	LQAAKDPS,-1
IVSRPEEL,-1	LAEEKLQA,-1	LDQGQLNK,-1	LGMLDPAE,-1	LLGIEQQV,-1	LQAEHELN,-1
IVSTTVET,-1	LAEIVQLV,-1	LDQPTQTV,-1	LGNVTTCN,-1	LLGQTDDT,-1	LQALSLAV,-1
IVSVDETI,-1	LAEKDEFE,-1	LDSLAANV,-1	LGQAEEVV,-1	LLGVADLT,-1	LQANVEHL,1
IVTEEAIA,-1	LAEKLPNL,-1	LDVAPLSL,-1	LGQNGISD,-1	LLGWNSFV,-1	LQDESFTR,-1
IVVIGHVD,-1	LAELEQLK,-1	LEANKIGF,-1	LGQTDDTR,-1	LLIGPNAP,-1	LQDIENAY,-1
IVVITDGR,-1	LAEPADFE,-1	LEASLEGD,-1	LGSEVVDK,-1	LLKPISVE,-1	LQDSGEVR,-1
IVVPDVSK,-1	LAEVAQHY,-1	LEASLESA,-1	LGSYGSLA,-1	LLKPNMVT,-1	LQDVSAST,-1
IWLYSLAW,-1	LAEVKPLV,-1	LEAVQNMV,-1	LGVADLTG,-1	LLLASGMA,-1	LQEELAAA,-1
IWNIKDEL,-1	LAFIQDPD,-1	LECADDRA,-1	LGVIACIG,-1	LLLFPLTA,-1	LQELFLAH,1

LQELVQYP,1	LVFLPFAD,-1	MECNLNEL,-1	NAVITVPA,-1	NLQDESFT,-1	PCHIEMIL,-1
LQEQLMGA,-1	LVFPSEIV,-1	MEISEDKT,-1	NCEVTNLN,-1	NLTHLNLS,-1	PCILGQNG,-1
LQFISSGL,-1	LVGALSES,-1	MEKQQQDQ,-1	NDDDETEV,-1	NLVLFDKA,1	PCIQAESE,1
LQFLLDTC,-1	LVGGASLK,-1	MENANVLA,-1	NDEELNQL,-1	NMSDDDGW,-1	PDFEPSTE,-1
LQGGQLEE,-1	LVGLFEDT,1	MEQGETPL,-1	NDELEIIE,1	NMVSHTER,-1	PDFKPPAD,-1
LQGSLLWA,-1	LVGPEGCG,-1	MESCGIHE,-1	NDGATILS,-1	NMVTPGHA,-1	PDGQMPSD,-1
LQGVHQGA,-1	LVGVDQFL,-1	MFDQSQIQ,-1	NDIKPIWQ,-1	NNATVTTC,-1	PDGYWIEI,1
LQKEEAQK,-1	LVHDPEFD,1	MFMAPGAG,-1	NDLNFIKQ,-1	NNEFIVIH,1	PDIVLLGK,-1
LQKMPLGK,-1	LVHNAQNL,-1	MFVMGVNH,-1	NDPQPEHP,-1	NNLLKPIS,-1	PDNTYEVK,-1
LQLVDAGK,-1	LVHPDLDR,-1	MGDIPAAV,-1	NEAIYDIC,-1	NPADSKPG,-1	PDSPSAQL,-1
LQPTPQVT,-1	LVIEHIQV,-1	MGHCDSVY,-1	NEALYDIC,-1	NPALADIY,-1	PDTNGSQF,-1
LQQAGDQE,-1	LVIGGGSG,-1	MHAYALEL,-1	NEANLSNL,-1	NPAPPIDA,-1	PDVVITEK,-1
LQREEAEN,-1	LVINYDLP,-1	MHLNVGLR,-1	NEATGGKY,-1	NPDFKPPA,-1	PEAPLAAG,-1
LQSHENQE,-1	LVIQSSPP,-1	MHTVCLSK,-1	NEEDAAEL,-1	NPEHNDFL,-1	PEEEVAQK,-1
LQSQVPNI,-1	LVKPSFDP,-1	MIEPIDEY,-1	NEELMASL,1	NPESGYNV,-1	PEEFEHMK,-1
LQYADPVS,-1	LVKSTLGP,-1	MIFDHEFT,-1	NEHSLVID,-1	NPNSTEHM,-1	PEELREDD,1
LRISSLEG,-1	LVLLSYVH,-1	MILNKPGL,-1	NEILTAII,1	NQLDQEVE,-1	PEEMIQTG,1
LSAFGDVS,-1	LVLNLQDE,-1	MILPVGAA,-1	NELLDYED,-1	NSAALIQQ,-1	PEFVDIIN,-1
LSAIAAMN,-1	LVLNSLAE,-1	MILPVGAE,-1	NENTFLDL,-1	NSEGGLHV,-1	PEGQAPVK,-1
LSATMPSD,-1	LVNDPQPE,-1	MKDPSVVV,-1	NEQFINAA,-1	NSEPASLL,-1	PEHNDFLK,-1
LSCHGWVL,-1	LVQEISFG,-1	MLDPAEKD,-1	NESTPPSE,-1	NSFNTDED,-1	PEILAIAP,1
LSDAIPGL,-1	LVQIEYAL,-1	MLETDVHE,1	NEVFLSLP,1	NSIATQGE,-1	PELIPQLV,-1
LSDEIKEE,-1	LVQMVVDG,-1	MLGDPCLK,-1	NFEDVAFD,1	NSLAEEKL,-1	PELLFIHG,1
LSDLNEVP,-1	LVSIPEDV,-1	MLGQAEEV,-1	NFEGEVTK,1	NSNGFICD,-1	PEMIGHYL,-1
LSDVCDVP,-1	LVSNLNPE,-1	MLGSYGSL,-1	NFEGLLWS,1	NSNTQVVL,-1	PERPFLAI,-1
LSEVGDTQ,-1	LVSSGEGK,-1	MLLANKVP,-1	NFNKSAPE,-1	NSSHAGAF,-1	PESGYNVS,-1
LSFFDHST,-1	LVSVLPEG,-1	MLNIINSS,-1	NFSKGEEN,-1	NSSSSNFE,-1	PEVDLVIQ,1
LSGDHCII,-1	LVTALCDI,-1	MMDVDHQI,-1	NFVFGQSG,1	NSVLLVSN,1	PEVIPENT,-1
LSGETAKG,-1	LVTEVENG,-1	MNDIKPIW,-1	NGGSLGSK,-1	NSVLVIID,1	PEVMVAFQ,1
LSGGTTMY,-1	LVTQQLVK,-1	MNFSKGEE,-1	NGIITNYK,-1	NTDEDTKK,-1	PFAPSGLK,-1
LSGLLDSP,-1	LVVAPAGI,-1	MNSFNTDE,-1	NGISDLVK,-1	NTDVPLVL,-1	PFLAILGG,-1
LSHTVEEK,-1	LVVGLCTG,-1	MNVAGVSL,-1	NGKEVAAQ,-1	NTIPHEVQ,-1	PGAGAIPK,-1
LSLGLETA,1	LVVPIIEN,-1	MNVDHEVN,-1	NGLTLGGQ,-1	NTIPQDEK,-1	PGCINYDL,-1
LSLLQDSG,-1	LVYAPPGK,-1	MNYEGSPI,-1	NGNLQLEL,-1	NTIPQEDK,-1	PGDYNINI,-1
LSLPCILN,-1	LVYEQDRP,-1	MPLSEEAI,1	NGNQPMEI,-1	NTLAEKDE,-1	PGEAEADG,-1
LSPSLEAR,-1	LWAHTLHS,1	MPSDVLEV,-1	NHEFLVKP,1	NTNVDKPL,-1	PGEYTSFL,1
LSPWGAEV,-1	LWESLENA,1	MQDATAQM,-1	NHFCEEFG,-1	NVAGVSLK,-1	PGFQLVFL,1
LSRPQDAL,-1	LWSQGQLH,-1	MQEFMILP,1	NHINVELS,1	NVCNENSL,-1	PGGFGAQG,-1
LSRPVLEG,-1	LYANTVLS,1	MSAFDPLK,-1	NIDGTHIA,-1	NVDHEVNL,-1	PGGSSTPL,-1
LSTSIAQL,-1	LYDLENLP,-1	MSDDDGWQ,-1	NIEVMNSF,-1	NVDLTEFQ,1	PGHACTQK,-1
LSVPCILG,-1	LYPEGQAP,-1	MSEFMECN,1	NIINSSIT,-1	NVRPDIVL,-1	PGLSQEAA,-1
LSYEVVDK,-1	LYPWGVVE,-1	MTEPIDEY,-1	NIKDELKK,-1	NVTNPNST,-1	PGNKVNLA,-1
LTAASEAA,-1	LYRGDVVP,-1	MTHGNTGF,-1	NILHDDCA,-1	NVTTCNDY,-1	PGPGGFGA,-1
LTAQHENF,-1	LYSLAWAG,-1	MTQAGVEE,-1	NILHVSEN,-1	NVVQLWAH,-1	PGPGLSQE,-1
LTDLYPER,-1	LYTLIVRP,1	MTTVHAIT,-1	NIPFTGNV,-1	NYDLPNNR,-1	PGTSFEFA,-1
LTDPDAPS,-1	LYTLVLTD,1	MVDNEAIY,-1	NIQGITKP,-1	NYDLPTNR,-1	PHMHAYAL,-1
LTGDLESF,-1	LYTLVTYV,1	MVTEEDKR,-1	NIVGLVGV,1	NYEAGKDD,-1	PHTKPWIG,-1
LTHLNLSG,-1	MACCLLYR,1	MVTPGHAC,-1	NKIGFEEK,-1	NYEGSPIK,-1	PIAAVMNT,-1
LTLNLEDV,-1	MAIATGGA,-1	MVVGESGL,-1	NKIPATQK,-1	NYKPPAQK,-1	PIDAVEQI,1
LTNLIHTK,-1	MAKDDLSG,-1	MVWEGLNV,-1	NKSAPELK,-1	NYQDTIGR,-1	PIDEYCVQ,-1
LTPVEEAP,-1	MAPGAGAI,-1	MVYPFTGD,-1	NKVNLAEL,-1	PAAAAAPA,-1	PIDVTEGE,1
LTTASGVS,-1	MASIPLPD,-1	MYGIENEV,-1	NKVPAAAR,-1	PADSKPGT,-1	PIDYTILD,1
LTTSAGIM,-1	MASLDQPT,-1	MYLNEVAG,-1	NKVTLDWA,-1	PADYKPPA,-1	PIIENTPE,-1
LTVADMER,-1	MATAASSS,-1	MYPGIADR,-1	NLCAIHAK,-1	PAGDALLQ,-1	PINAFIGG,1
LVADKVIV,-1	MATVTALR,-1	NAATEDLW,-1	NLDGVANA,-1	PAGHQEEA,-1	PIPEEYTF,-1
LVAETFAQ,-1	MAVEFLHE,1	NACWELYC,-1	NLEDVQPH,-1	PAHAVDAV,-1	PISVEGSS,-1
LVDAGKVD,-1	MDAELEFA,1	NALDNVDA,-1	NLEGEETF,-1	PALNEANL,-1	PIWQRPSK,-1
LVDLDHQR,-1	MDPLGGSD,-1	NALYPEGQ,-1	NLGDQQSF,-1	PAQTSLEY,-1	PKDVESYI,-1
LVDSVLDV,-1	MDQVENHE,-1	NAPLIFER,-1	NLISFTEV,-1	PATGFIDG,-1	PKPEEEVA,-1
LVDVIEDK,-1	MDTLLATL,-1	NAQNLMQS,-1	NLLKPISV,-1	PAVSEKDI,-1	PLAAGIVG,-1
LVEDKGNQ,-1	MDVDHQIA,-1	NASGKPIA,-1	NLMDAQVK,-1	PAYFNDSQ,-1	PLDAVIEA,1
LVETPTGY,-1	MDVIQHET,-1	NASNNELV,-1	NLNFNKSA,-1	PCDFLIPV,1	PLELDLTG,1
LVFEAPNQ,-1	MEAIAPPL,-1	NATVTTCH,-1	NLPGAAVD,-1	PCEQLVTA,1	PLEMEQQQ,1

PLGECIIA,-1	PVEHPDKF,-1	QGAYQEAF,1	QSEADADI,-1	SDGIMVAR,-1	SHENQEIY,-1
PLGGSDTA,-1	PVGAANFR,-1	QGETPLTM,-1	QSFLQPGG,-1	SDLNEVPV,-1	SHFPMTHG,-1
PLLGGSLM,-1	PVGAESFR,-1	QGHLLPER,-1	QSGAGNNW,-1	SDNAPPPE,-1	SHQDADIE,-1
PLLGWNSF,-1	PVLLDALT,-1	QGHQDQSI,-1	QSHENQEI,-1	SDVCDVPT,-1	SHQDLSQK,-1
PLLLASGM,-1	PVLVGALS,-1	QGITKPAI,-1	QSHFPMTH,-1	SDVLEVTK,-1	SHVSFPLE,-1
PLLSGLLD,-1	PVNVTTEV,-1	QGQLHGLK,-1	QSIISTVE,-1	SEAAEQGR,-1	SIASVADT,-1
PLLVAETF,-1	PVQDLGST,-1	QGRPPEHT,-1	QSQIQEFK,-1	SEAALQAI,-1	SIATQGEL,-1
PLMGVIYV,1	PVQTQHPI,-1	QGSLLWAA,-1	QSQVPNIV,-1	SEADADIL,-1	SIDSVEDH,1
PLNMILDD,1	PVSAQHAK,-1	QGVHQGAG,-1	QSSANYAE,-1	SEALLVTQ,1	SIEDSVIS,1
PLPMMPLS,-1	PVSPLLLA,-1	QHKPSAPQ,-1	QSSPPKDV,-1	SEEFLSFF,1	SIENIAFG,1
PLSCHGWV,-1	PVTNAVIT,-1	QHYQDTLI,-1	QTGISAID,-1	SEEGETEA,-1	SIIHSSLA,-1
PLTAQHEN,-1	PVVAMVWE,1	QIAAENEE,-1	QTIDNSQG,-1	SEFTLASD,-1	SIISTVEP,-1
PLTMLQSQ,1	PVVDHEFD,-1	QIIEACDV,-1	QTLLDAGL,-1	SEGGLHVD,-1	SIPEDVVQ,-1
PLVLMNSF,1	PVVQSDMK,-1	QILHSEEF,-1	QTNLVPYP,-1	SEGLWEIE,1	SITYKPVK,-1
PMFVMGVN,-1	PVWSGMNV,-1	QILPTLVR,-1	QTVDGRPC,-1	SEHLKNPV,-1	SIVMITKL,1
PMTHGNTG,-1	PWGPKPFR,-1	QIVPKPEE,-1	QVAGTPMF,-1	SEHSMDTL,-1	SKGEENLM,-1
PNAPLIFE,-1	PWIGLAEA,1	QLALIAAS,1	QVCNPIIS,-1	SEIGAEEA,-1	SLAEEKLQ,-1
PNFPSPEY,-1	QAAKDPSA,-1	QLDGLNAS,-1	QVIQSLIA,-1	SEISTWDG,-1	SLDLFNCE,1
PNIELSYE,1	QADLYISE,1	QLEEVILQ,1	QVLAQERP,-1	SEKDIQDL,-1	SLDQPTQT,-1
PNLTHLNL,-1	QAEEVVQE,-1	QLELAQVL,1	QVSSLLGE,-1	SEKWQQAL,-1	SLEDLIFE,1
PNMVTPGH,-1	QAEHELNL,-1	QLEPTFIK,-1	QVTPPGFQ,-1	SELAEDKE,-1	SLEGDSGR,-1
PNSTEHMK,-1	QAFQTIAR,-1	QLIDDHFL,-1	QVTQEQGH,-1	SENDLNFI,-1	SLELFMYL,1
PPADYKPP,-1	QAGDQELL,-1	QLIGIQDG,-1	QVVLLSAT,1	SENKIPAT,-1	SLEYALLD,1
PPELLEII,1	QALSLAVD,-1	QLKPDVVI,-1	QVVQVVLD,1	SEPASLLE,-1	SLFDKDGD,-1
PPKDVESY,-1	QAMPTLIE,-1	QLNGNLQL,-1	QYADPVSA,-1	SEVELVQM,1	SLFLTDLY,-1
PPLLQELK,-1	QAQPVLVF,-1	QLQEELAA,-1	QYFGILLD,1	SEVGDTQV,-1	SLGLPFGK,-1
PPSAEVEA,-1	QASPLLQY,-1	QLQGGQLE,-1	QYNVVQLW,-1	SFDPNLSE,-1	SLIALVND,1
PPSQPEDL,-1	QATDFGEA,-1	QLVANVTN,1	QYPVEHPD,-1	SFEFALAI,1	SLIYTNYE,1
PQALIDQL,1	QAVEVVIT,1	QLVDAGKV,-1	QYQEPIPC,-1	SFELLSHT,1	SLKDYCTR,-1
PQALSEHL,1	QAYRPEMK,-1	QMAEIAVN,-1	RDGDILGK,-1	SFEYIEAH,1	SLKPEFVD,-1
PQAQPVLV,-1	QDADIEDF,-1	QMAMLQFI,1	RDMLLANK,-1	SFFDHSTR,-1	SLLQDSGE,-1
PQPEHPLR,-1	QDATAQMA,-1	QMHLNVGL,-1	REDDVGTG,-1	SFGTTKDK,-1	SLLWAAGQ,-1
PQPNFPSP,-1	QDGSLDLN,-1	QMVVDGVK,-1	REEAENTL,-1	SFIGAIAI,1	SLPCILNA,-1
PQVSLIIN,1	QDGSQNTN,-1	QNGISDLV,-1	RISSLEGR,-1	SFLAPISS,-1	SLPENASH,-1
PQVTQEQG,-1	QDIENAYK,-1	QNLDGVAN,-1	RPDIVLLG,-1	SFLDISRP,-1	SLSDLNEV,-1
PSADAPMF,1	QDILDLVG,-1	QNLMQSVK,-1	RPDNTYEV,-1	SFLGMESC,1	SLSEYVSR,-1
PSAFVAAA,1	QDLGSTVI,-1	QNMVSHTE,-1	RPFLAILG,-1	SFLNDVGP,-1	SLSGDHCI,-1
PSALAILE,1	QDTSRPPL,-1	QNPANMSK,-1	RPGPGLSQ,-1	SFLQPGGK,-1	SLVFPLLV,1
PSALAIME,1	QDVAQNPA,-1	QNTNVDKP,-1	RPPEHTSK,-1	SFNGALAA,1	SLVIDTLK,-1
PSAPQGGR,-1	QDVDLAEV,1	QNVPLEDR,-1	RPPLEYVK,-1	SFNTDEDT,-1	SMDTLLAT,-1
PSDECGAG,-1	QDVSASTK,-1	QPDGQMPS,-1	RPPSEMAD,-1	SFSEVQGM,-1	SMDVIQHE,-1
PSDVLEVT,-1	QEAASLLG,-1	QPGCINYD,-1	RPQDALEG,-1	SGAGNNWA,-1	SMMDVDHQ,-1
PSEELELD,1	QEAFDISK,-1	QPNFPSPE,-1	RPTGGVGA,-1	SGDHCIIG,-1	SMSAFDPL,-1
PSEIVGKK,-1	QEDWLVSV,1	QPTPQVTQ,-1	RPVLEGLR,-1	SGELNQYS,-1	SNGFICDY,-1
PSFDPNLS,-1	QEELAAAE,-1	QPTQTVVM,-1	SAALIQQA,-1	SGETAKGD,-1	SNIDGTHI,-1
PSPVTAQK,-1	QEEMLQRE,1	QQADFLDA,-1	SADESGQI,-1	SGETEDTF,-1	SNSAALIQ,-1
PSQPEDLS,-1	QEESIKPK,-1	QQAGDQEL,-1	SAFGDVSK,-1	SGGEEGAR,-1	SNSSSSNF,-1
PSVFAEVP,1	QEHCSSIV,-1	QQAYRPEM,-1	SAGIMDHE,-1	SGGGLLQK,-1	SNVFAMFD,1
PTALLAHE,1	QEISFGTT,-1	QQELQEYE,1	SAGQGEVL,-1	SGGLAAAK,-1	SPAHAVDA,-1
PTGGVGAV,-1	QELLHQAK,-1	QQHGGVSL,-1	SAIAAMNE,-1	SGGLASAR,-1	SPCHIEMI,-1
PTGTYHGD,-1	QEMATAAS,-1	QQIEHLNK,-1	SAIDGMNS,-1	SGGTTMYP,-1	SPDGHLFQ,-1
PTGYIESL,-1	QEPIPCEQ,-1	QQILHSEE,-1	SALILHDD,-1	SGKPIAAV,-1	SPEYMIFD,1
PTPQVTQE,-1	QEQGHLLP,-1	QQLIDDHF,-1	SAPGPLEL,-1	SGLLDSPA,-1	SPHTKPWI,-1
PTQEELEA,-1	QEVEFLST,1	QQMHLNVG,-1	SASASFFL,-1	SGMDEMAV,-1	SPLLLASG,-1
PTQTVVMH,-1	QEYAQVIK,-1	QQQAYRPE,-1	SASFFLDK,-1	SGMNVAGV,-1	SPLLQYFG,-1
PTQWLDGK,-1	QEYEVVTE,-1	QQQDQVDR,-1	SATMPSDV,-1	SGNVVNKK,-1	SPPKDVES,-1
PTVFFDIA,1	QFASQEIA,-1	QQQLIDDH,-1	SAVIHSEN,-1	SGPSIVHR,-1	SPSAQLAL,-1
PTVIDEVR,-1	QFGFIVLT,1	QQSHFPMT,-1	SCGIHETT,-1	SGSLSSTS,-1	SPVEDNEK,-1
PVAAATTA,-1	QFGVGFYS,-1	QQVLWNCL,-1	SDAIPGLK,-1	SGSSHQDL,-1	SPWGAEVK,-1
PVDFVTAD,1	QFISSGLS,-1	QQVSLVIN,1	SDAYYCTG,1	SGVCIDSE,-1	SQATDFGE,-1
PVDISDSD,-1	QFLLDTCK,-1	QREEAENT,-1	SDDDGWQF,-1	SGVQHKPS,-1	SQAVSNAR,-1
PVDYLLGV,1	QGAGFQDI,-1	QRPGPGLS,-1	SDECGAGV,-1	SGVSGGEE,-1	SQEESIKP,-1
PVEDNEKD,-1	QGAGGPGP,-1	QRPPSEMA,-1	SDEIKEER,-1	SGVSVLPQ,-1	SQFGTVEK,-1

SQGQLHGL,-1	TAFLHPEE,-1	THINIVVI,1	TPTGYIES,-1	VAGGAPSV,-1	VELQKEEA,-1
SQNTNVDK,-1	TAGGVMTA,-1	THIPGSPF,-1	TPVEEAPK,-1	VAGTPMFV,-1	VELSLLGK,-1
SQPEDLSL,-1	TAIIQGMR,-1	THLAPGTK,-1	TQAMAFDG,-1	VAHIIDPK,-1	VENGGSLG,-1
SQPGVDGF,-1	TAISLFYE,-1	THLNLSGN,-1	TQEELEAV,1	VAINMVTE,1	VENPEHND,-1
SQQADFLD,-1	TAKGDYPL,-1	THQTSLEL,-1	TQEQGHLL,-1	VALAQAVN,-1	VEQAFQTI,-1
SQSFLQPG,-1	TAQHENFR,-1	TIDNSQGA,-1	TQGHQDQS,-1	VALDFEQE,1	VEQILPTL,-1
SQVAGTPM,-1	TASGVSVL,-1	TIGEILKK,-1	TQSEADAD,-1	VALINAAI,-1	VEQLLQDP,-1
SQVPNIVK,-1	TAVHDAIL,-1	TIHLPSPV,-1	TQTVVMHR,-1	VALMKEEG,-1	VESDADEE,-1
SRPPLEYV,-1	TDEDTIID,1	TIIDIITH,-1	TQVMAASM,1	VALNPDFK,-1	VESTGVFT,-1
SRPQDALE,-1	TDFGEALV,-1	TIIDILTK,-1	TSAGIMDH,-1	VALSDVCD,-1	VETAAGGD,-1
SRPVLEGL,-1	TDPDAPSR,-1	TIIGKEDA,-1	TSASGDEM,-1	VALVHPDL,-1	VETGVLKP,-1
SSAMLESL,1	TDQVIQSL,-1	TIIGSFNG,-1	TSAVIHSE,-1	VANALDNV,-1	VETKEPEK,-1
SSANYAEN,-1	TDVNSILR,-1	TIIPVLVG,-1	TSAYLQDI,1	VANDEELN,-1	VETPTGYI,-1
SSAPGPLE,-1	TEANNHKK,-1	TILDDIGH,-1	TSEIGAEE,-1	VAPAGITL,-1	VEVVITHL,-1
SSGNVVNK,-1	TECCHGDL,-1	TILEGHLG,-1	TSFLAPIS,-1	VAPESFEY,-1	VEWIPNNV,-1
SSGSLSST,-1	TEDKINAL,-1	TIPHEVQK,-1	TSIASVAD,-1	VAQHYQDT,-1	VEYAQEAV,-1
SSHQDLSQ,-1	TEDTFIAD,-1	TIQQCCEN,-1	TSLSEYVS,-1	VAQNPANM,-1	VFACLMGK,-1
SSLLGEED,-1	TEDTFMAD,-1	TISSSLAV,-1	TSQSGDEM,-1	VAQQVLWN,-1	VFAKPPDQ,-1
SSPCHIEM,-1	TEEEYEDS,1	TIVDDDDY,-1	TSRPPLEY,-1	VASYLLAA,1	VFALTNGI,1
SSPPKDVE,-1	TEFQTNLV,-1	TKENLLDF,-1	TTAAPAAA,-1	VAVTMGPK,-1	VFEAPNQE,-1
SSQAVSNA,-1	TEGAELVD,-1	TKLVEVGR,-1	TTASGVSV,-1	VCAAYAHE,-1	VFGEEGLT,-1
SSSGSLSS,-1	TEGEVISL,-1	TKPSDEEM,-1	TTFNSIMK,-1	VCDVPTAK,-1	VFIFWAPE,1
SSSLAVVD,-1	TEHAHQVV,-1	TLADDVTL,-1	TTHQTSLE,-1	VCIESEHS,1	VFLPFADD,-1
SSSRSASR,-1	TEHVAAAE,-1	TLAEKDEF,-1	TTLDESLT,-1	VCNENSLF,-1	VFPSEIVG,-1
SSSSNFEG,-1	TEIRDMLL,-1	TLAPTQWL,-1	TTLEHSDC,-1	VCNPIISG,-1	VFSPDGHL,-1
SSSSRSAS,-1	TEKEQIVP,-1	TLASSQAV,-1	TTMYPGIA,-1	VDAGKVDD,-1	VFVLDEAD,-1
SSSSSRSA,-1	TELAEDKE,-1	TLDDLIMA,1	TTPQTVLF,-1	VDAVIAEL,-1	VGAGGAGL,-1
SSTPLIPK,-1	TELPPTHP,-1	TLDESLTN,-1	TTQAMAFD,-1	VDDLDFFI,-1	VGALSESK,-1
SSTSSWYS,1	TEPIDEYC,-1	TLDWAKPK,-1	TTSAGIMD,-1	VDEATIID,1	VGAPMHDL,-1
SSVPVWSG,-1	TEPTTAFN,-1	TLEHSDCA,-1	TTSAVIHS,-1	VDETIKNP,-1	VGCVAGDE,-1
SSVYPTQE,-1	TESFQVKR,-1	TLGSEVVD,-1	TTVETKEP,-1	VDFLSDEI,-1	VGDAIPAV,-1
SSYFVEWI,1	TEVENGGS,-1	TLIELMKD,1	TTVHAITA,-1	VDGEPLGR,-1	VGDTQVVV,-1
STAISLFY,-1	TEVLKEDI,1	TLINSLFL,1	TVAESAEE,-1	VDGFLVGG,1	VGESGLGK,-1
STCPDDEE,-1	TFAPVNVT,-1	TLIVVPDV,-1	TVDGRPCK,-1	VDIINAKQ,-1	VGGASLKP,-1
STESLQFL,1	TFDDHDPV,-1	TLKPSTLR,-1	TVELLVED,1	VDISDSDM,-1	VGIYEVTH,1
STGVFTTM,-1	TFDDHDSV,-1	TLLATLKK,-1	TVETKEPE,-1	VDLEPTVI,-1	VGLCTGQI,-1
STSIAQLK,-1	TFEAAILT,1	TLLDAGLP,-1	TVFACLMG,-1	VDNEAIYD,-1	VGNLNFNK,-1
STTVETKE,-1	TFEAAMLG,1	TLLEGSGL,-1	TVGCVAGD,-1	VDPLDGTK,-1	VGPEAPLA,-1
STVEPAQR,-1	TFIADLVV,1	TLLKPNMV,-1	TVHAITAT,-1	VDSVLDVV,-1	VGPEGCGK,-1
SVAAMVYP,1	TFITDCKD,-1	TLMVVGES,-1	TVLCELIN,-1	VDVFREDL,1	VGSGVSGG,-1
SVADTAEE,-1	TFKEEEPV,-1	TLNDELEI,-1	TVLSGGTT,-1	VDVGDVDA,-1	VGSPVEDN,-1
SVDETIKN,-1	TGAGLLEI,-1	TLNLEDVQ,-1	TVPAYFND,-1	VDVIDQNR,-1	VGSQATDF,-1
SVISLSGD,-1	TGDLESFK,-1	TLPDTLQG,1	TVQQIEHL,-1	VDVIEDKL,-1	VGTGAGLL,-1
SVLDVVRK,-1	TGDVTAWT,-1	TLPTEYDS,-1	TVTEDKIN,-1	VDVLVSSG,-1	VGVDQFLV,-1
SVLPEGSR,-1	TGETGIEE,-1	TLQGSLLW,-1	TVTNDGAT,-1	VDWCPTGF,-1	VGVEHLLR,-1
SVLRISSL,-1	TGFIDGDL,-1	TMPSDVLE,-1	TVTTCHSK,-1	VEAANYQD,-1	VGVYEALK,1
SVNYKPPA,-1	TGGAVFGE,-1	TMYPGIAD,-1	TWDGVIVT,1	VEAGDVIY,-1	VHAITATQ,-1
SVPAPACA,-1	TGGKYVPR,-1	TNAVITVP,-1	TYHGDSDL,-1	VEAIVLPM,-1	VHDAILED,-1
SVPCILGQ,-1	TGGMAFHS,-1	TNDGATIL,-1	TYVPVTTF,-1	VEALNGKE,-1	VHILASVL,1
SVPVWSGM,-1	TGGVGAVA,-1	TNGIYPHK,-1	VAAAELGA,-1	VEDHLAWS,-1	VHNAQNLM,-1
SVYPTQEE,-1	TGIFLDLM,1	TNLCAIHA,-1	VAAAPVAA,-1	VEDIIDTG,-1	VHQGAGFQ,-1
SWEGAFQH,1	TGISAIDG,-1	TNLVPYPR,-1	VAAATTAA,-1	VEDKGNQV,-1	VHTECCHG,-1
SWYSSSSS,-1	TGIVQGLK,-1	TNPADSKP,-1	VADKVIVT,-1	VEDNEKDL,-1	VHVLCNSE,-1
SYDYLVIG,1	TGKITDFI,-1	TNPNSTEH,-1	VADLTGEL,-1	VEDPAGHQ,-1	VIAADMLG,-1
SYEVVDKD,-1	TGTYHGDS,-1	TNTIPHEV,-1	VADTAEEK,-1	VEEGIVLG,1	VIACIGEK,-1
SYSMIVNN,1	TGVFTTME,-1	TNTIPQDE,-1	VADTNNHQ,-1	VEELAEQL,1	VIAGLNVL,-1
TAAGGDGA,-1	TGVLKPGM,-1	TNTIPQED,-1	VADVSIED,-1	VEFATHED,-1	VIEAEHTL,-1
TAAPAAAA,-1	TGYIESLP,-1	TNVDKPLR,-1	VADVTTPQ,-1	VEFATHEE,-1	VIEHIQVN,-1
TAASEAAC,-1	THEDAVAA,-1	TNYEAGKD,-1	VAECFDEI,-1	VEFVTHED,-1	VIFTDVNS,-1
TAASSSSL,-1	THEEAVAA,-1	TPDMMEEM,1	VAESAEEE,-1	VEGVAHII,-1	VIGGGSGG,-1
TADKFDEN,-1	THETAFLG,-1	TPGHACTQ,-1	VAETFAQK,-1	VEHPDKFL,-1	VIGHVDSG,-1
TADNLVLN,1	THGHVGAD,-1	TPQTVLFK,-1	VAFQDVAQ,-1	VELCPILP,-1	VIHNGIIT,-1
TADSQPPV,-1	THGNTGFS,-1	TPQVTQEQ,-1	VAGDEESY,-1	VELNGNQP,-1	VIIDVKPK,-1

VIISAPSA,-1	VNLPGAAV,-1	VTKENLLD,-1	WIEILNPN,-1	YSAFLVAD,1	
VILDAGAQ,-1	VNNLLKPI,-1	VTLAPTQW,-1	WLDANTLA,-1	YSALILHD,-1	
VILDDVDS,-1	VNPESGYN,-1	VTLDWAKP,-1	WLYSLAWA,-1	YSSSSSRS,-1	
VILQAEHE,-1	VNVTTEVK,-1	VTNAVITV,-1	WNCLIEDP,1	YTEGAELV,1	
VINYDLPT,-1	VNYKPPAQ,-1	VTNDGATI,-1	WNIKDELK,-1	YTGIVQGL,-1	
VIPGGSST,-1	VPAPACAL,-1	VTNLNDYR,-1	WNNATVTT,-1	YTSASGDE,-1	
VIQAMPTL,-1	VPAYFNDS,-1	VTNPNSTE,-1	WNSFVVWK,-1	YVADTNNH,-1	
VIQHETIG,-1	VPCGNIVG,-1	VTNTIPHE,-1	WPELIPQL,-1	YVNGLTLG,-1	
VIQSSPPK,-1	VPCILGQN,-1	VTNTIPQD,-1	WPQEAGEY,-1	YVPLTDKE,-1	
VISASASF,-1	VPFAPSGL,-1	VTNTIPQE,-1	WQEHCSSI,-1	YVPVTTFK,-1	
VISLGLPF,-1	VPIIENTP,-1	VTPGHACT,-1	WQPDTEEE,-1	YVQQHGGV,-1	
VISLSGDH,-1	VPKPEEEV,-1	VTPPGFQL,-1	WSADESGQ,-1	YVSNIDGT,-1	
VITHLAPG,-1	VPLTDKEK,-1	VTQEQGHL,-1	WSGMNVAG,-1	YWPQEAGE,-1	
VITKPDVY,-1	VPQAQPVL,-1	VTSEIGAE,-1	WSQGQLHG,-1	YYTSASGD,-1	
VITVPAYF,-1	VPVQDLGS,-1	VTSIASVA,-1	WVDPLDGT,-1		
VIVTEEAI,-1	VPVWSGMN,-1	VTTPQTVL,-1	WYSSSSSR,-1		
VIVTPSEK,-1	VQDLGSTV,-1	VTVTEDKI,-1	YADASLVF,-1		
VIVVITDG,-1	VQEISFGT,-1	VTYVPVTT,-1	YADPVSAQ,-1		
VIYVPLTD,-1	VQFASQEI,-1	VVAPAGIT,-1	YAENFIQS,1		
VKEVSTYI,-1	VQFVDMVK,-1	VVDHEFDA,-1	YAPPGKEK,-1		
VKPSFDPN,-1	VQGMVELN,1	VVDKDSIR,-1	YAQEAVKK,-1		
VKSTLGPK,-1	VQHKPSAP,-1	VVESTGVF,-1	YASPAHAV,-1		
VLAEVAQH,-1	VQMVVDGV,-1	VVGAGGAG,-1	YCIDNEAL,1		
VLAIFNLL,-1	VQNMVSHT,-1	VVGESGLG,-1	YCLEHGIQ,-1		
VLAKPPDK,-1	VQPHDLGK,-1	VVGLCTGQ,-1	YCTGDVTA,-1		
VLAQERPK,-1	VQQHGGVS,-1	VVGPEAPL,-1	YDDMLVVP,1		
VLCNSEDI,-1	VQQIEHLN,-1	VVHPGDLK,-1	YDLENLPA,-1		
VLDEADVM,-1	VQRPGPGL,-1	VVIAADML,-1	YDYGPHFQ,-1		
VLDGADCI,-1	VQTQHPIR,-1	VVIGHVDS,-1	YEAGKDDY,-1		
VLDGLSNI,-1	VQYPVEHP,-1	VVILDAGA,-1	YEDSSGNV,-1		
VLDQVEAR,-1	VRPDIVLL,-1	VVITHLAP,-1	YEFLTPVE,-1		
VLEQVVTS,1	VRPDNTYE,-1	VVLDGLSN,-1	YEIDLQKM,-1		
VLFHHEGG,-1	VSEDFLQD,1	VVLGHSER,-1	YENVRPDI,-1		
VLGFLEAN,1	VSEFYEET,1	VVLSPSLE,-1	YEQDRPLK,-1		
VLGGGCAL,-1	VSEKDIQD,-1	VVMEKPNI,-1	YEVVDKDS,-1		
VLGHELGH,-1	VSENVIFT,1	VVPIIENT,-1	YEVVTESE,-1		
VLHDPETL,-1	VSFPLEGL,-1	VVTESEKR,-1	YFEYIEEN,1		
VLISEAQA,-1	VSFSEVQG,-1	VVTFAPVN,-1	YFPIPEEY,-1		
VLLDALTD,-1	VSGGEEGA,-1	VVTNTIPH,-1	YGIENEVF,-1		
VLLGIEQQ,-1	VSILMASI,1	VVTNTIPQ,-1	YHEMIESG,1		
VLLSYVHI,-1	VSIPEDVV,-1	VVTSIASV,-1	YHGDSDLQ,-1		
VLNLQDES,-1	VSLKDYCT,-1	VVVGAGGA,-1	YHTSQSGD,-1		
VLNSLAEE,-1	VSMDVIQH,-1	VVVGPEAP,-1	YIPLPDEK,-1		
VLQPTPQV,-1	VSNIDGTH,-1	VVVTNTIP,-1	YIQQLCED,-1		
VLRISSLE,-1	VSNLNPER,-1	VVWVDPLD,-1	YISEGLHP,-1		
VLSGGTTM,-1	VSNSAALI,-1	VVYNASNN,-1	YISLIYTN,-1		
VLSPSLEA,-1	VSPLLLAS,-1	VWEGLNVV,-1	YLNEVAGK,-1		
VLTDPDAP,-1	VSSLLGEE,-1	VWSGMNVA,-1	YLSLLQDS,-1		
VLTTSAGI,-1	VSTTVETK,-1	VWVDPLDG,-1	YNASNNEL,-1		
VLTVADME,-1	VSVDETIK,-1	VYADASLV,-1	YNEATGGK,-1		
VLVGALSE,-1	VSVLPEGS,-1	VYAPPGKE,-1	YNTDVPLV,-1		
VLVSSGEG,-1	VSVLPQNR,-1	VYEQDRPL,-1	YNVSLLYD,1		
VLVYVEDP,1	VTADKFDE,-1	VYNASNNE,-1	YNVVQLWA,-1		
VMDPLGGS,-1	VTALCDIK,-1	VYPFTGDH,-1	YPEGQAPV,-1		
VMEKPNIR,-1	VTDFFLQL,1	VYPTQEEL,-1	YPFTGDHK,-1		
VMGVNHEK,-1	VTEDKINA,-1	WAPESAPL,-1	YPTQEELE,-1		
VNDFLAEI,1	VTEEAIAK,-1	WARPPISM,-1	YPVEHPDK,-1		
VNDPQPEH,-1	VTEVENGG,-1	WASFLNDV,-1	YPVTNAVI,-1		
VNDTIQID,-1	VTFAPVNV,-1	WDNLLAVI,1	YPVVDHEF,-1		
VNGLTLGG,-1	VTFDDHDP,-1	WEDMEDLV,-1	YQEPIPCE,-1		
VNHFCEEF,-1	VTFDDHDS,-1	WEGLNVVK,-1	YQGAGGPG,-1		
VNLAELFK,-1	VTGGWDNL,-1	WEIENNPT,-1	YQSNTILR,-1		
VNLGDQQS,-1	VTHEDAVA,-1	WGDAGAEY,-1	YRGDVVPK,-1		
