# Supplementary material for: Prediction of HIV-1 protease cleavage site using a combination of sequence, structural, and physicochemical features
Source: BMC Bioinformatics. 2016 Dec 23;17(Suppl 17):478. doi: 10.1186/s12859-016-1337-6 (PMC5259813; doi:10.1186/s12859-016-1337-6)

Additional File 5: The ROC plots for the four benchmark datasets

Figure S1. The ROC plots of training, validation, and test sets for the 746 dataset.

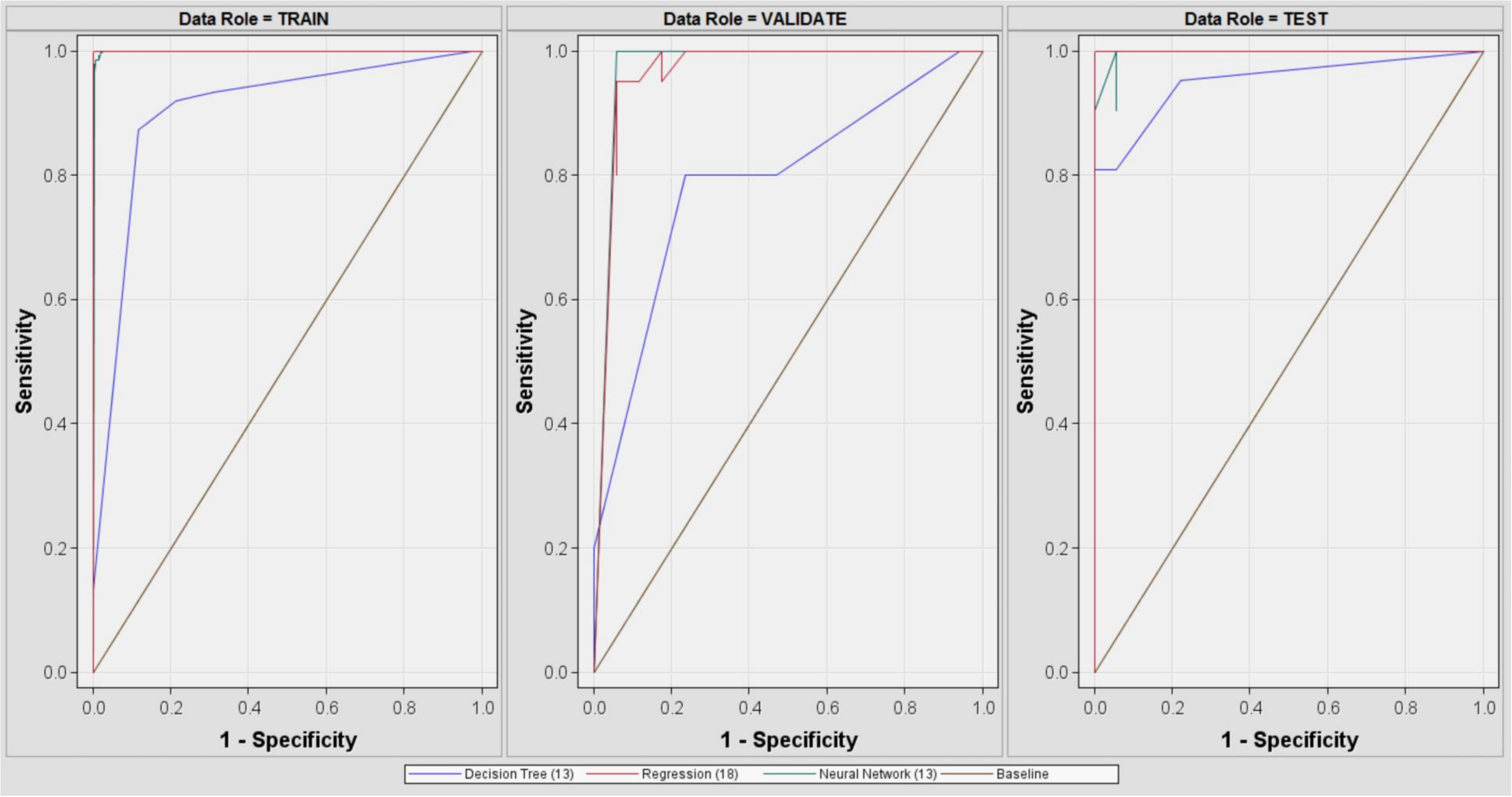

Figure S2. The ROC plots of training, validation, and test sets for the 1625 dataset.

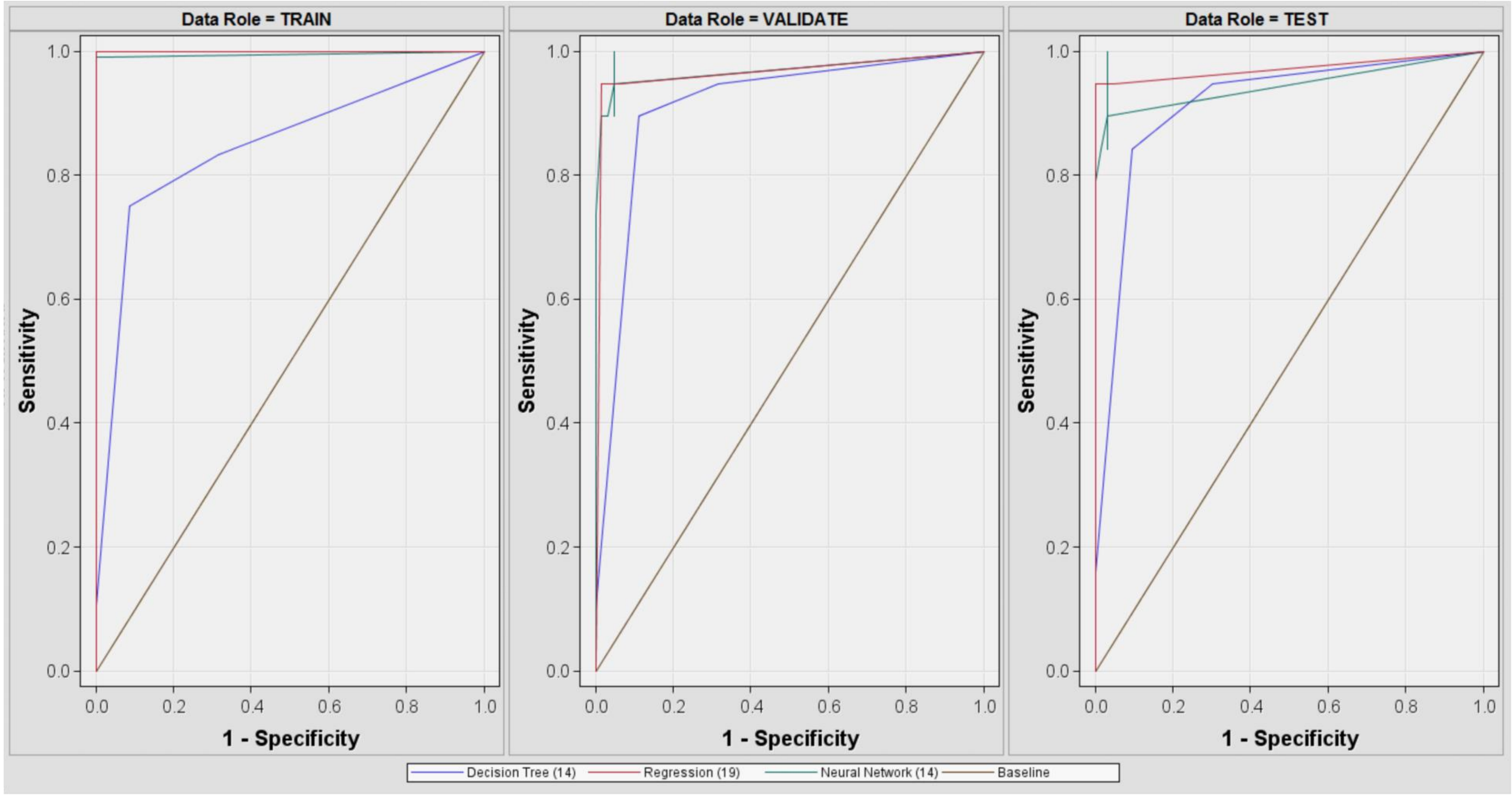

Figure S3. The ROC plots of training, validation, and test sets for the Schilling dataset.

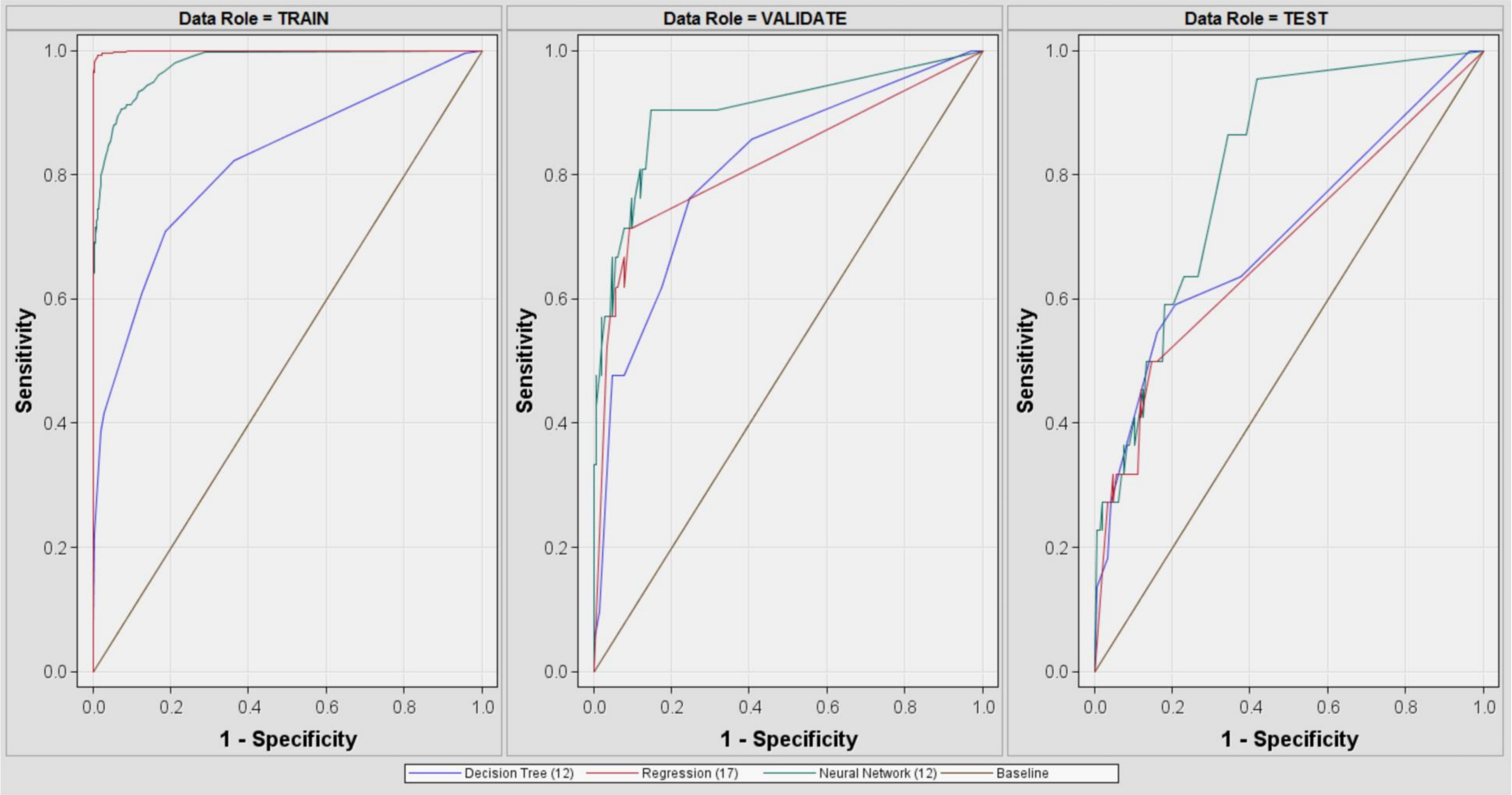

Figure S4. The ROC plots of training, validation, and test sets for the Impens dataset.

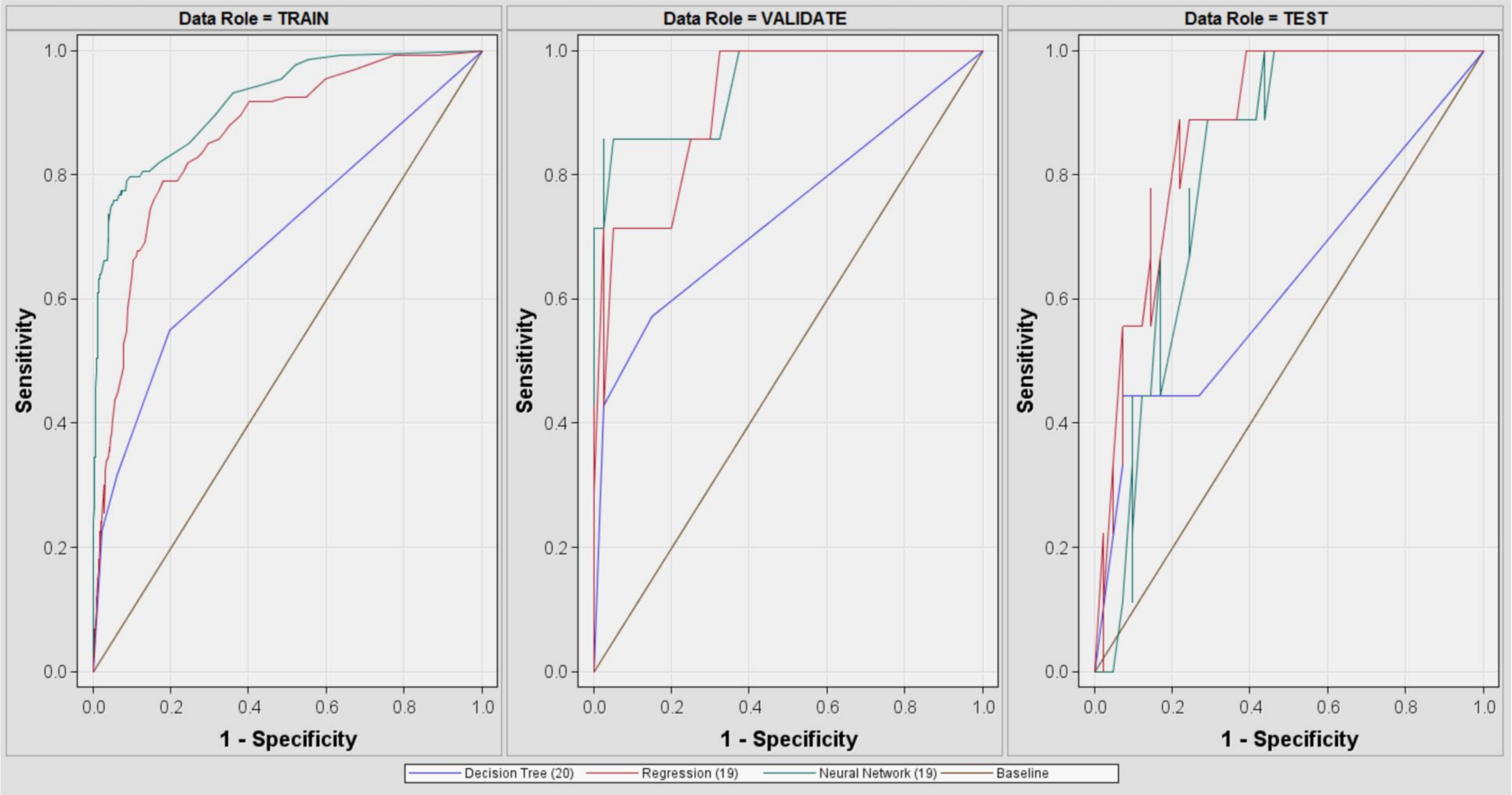

Supplement: Additional file 5: — The ROC plots for the four benchmark datasets. (PDF 675 kb) [file 12859_2016_1337_MOESM5_ESM.pdf]
