# Supplementary material for: Prediction of HIV-1 protease cleavage site using a combination of sequence, structural, and physicochemical features
Source: BMC Bioinformatics. 2016 Dec 23;17(Suppl 17):478. doi: 10.1186/s12859-016-1337-6 (PMC5259813; doi:10.1186/s12859-016-1337-6)

Additional File 6: Decision trees of the 1625, Schilling, and Impens datasets.

Figure S5. Decision tree of the 1625 dataset based on Seq+Str features.

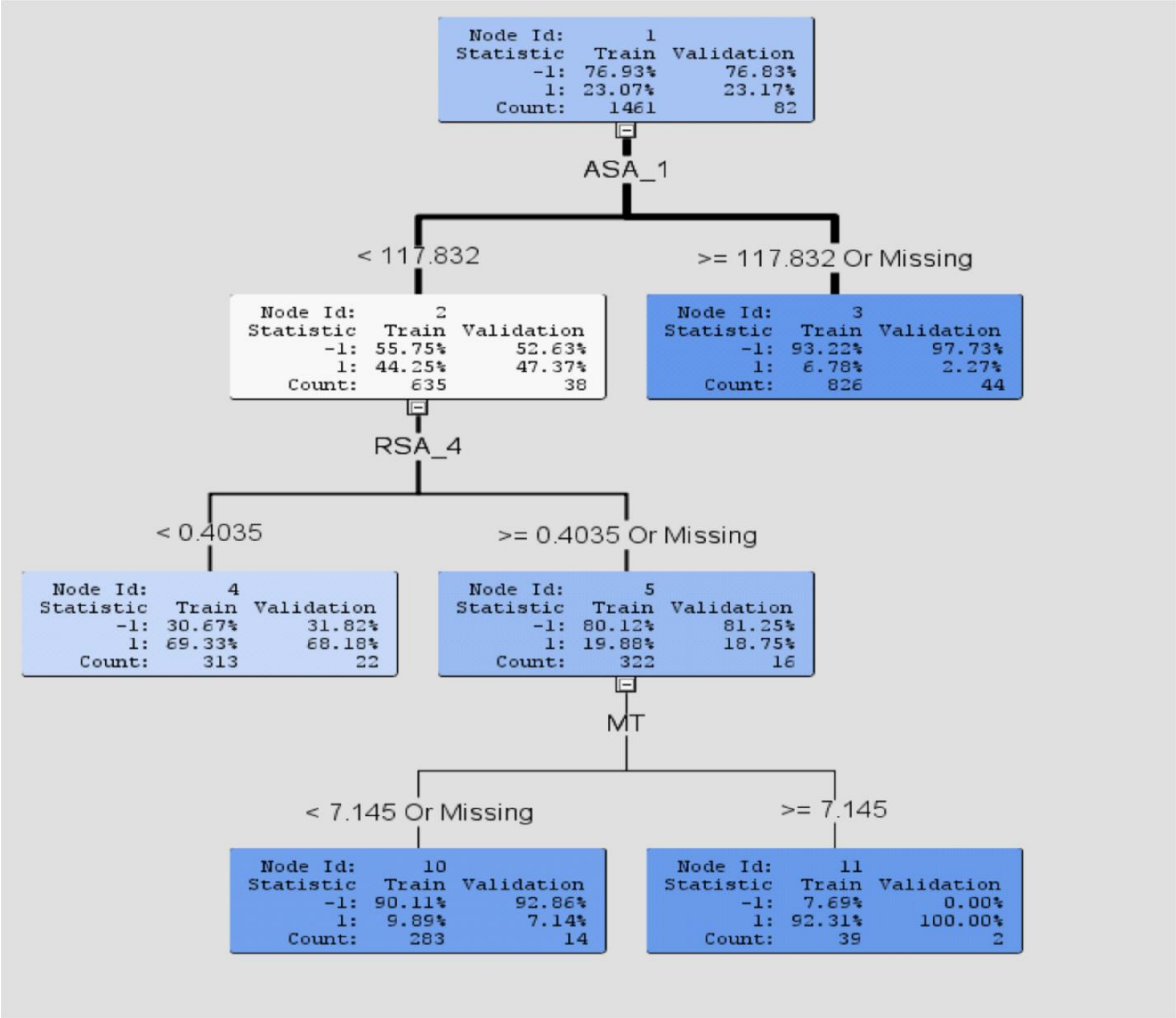

Figure S6. Decision tree of the Schilling dataset based on Seq+Str+Phy features.

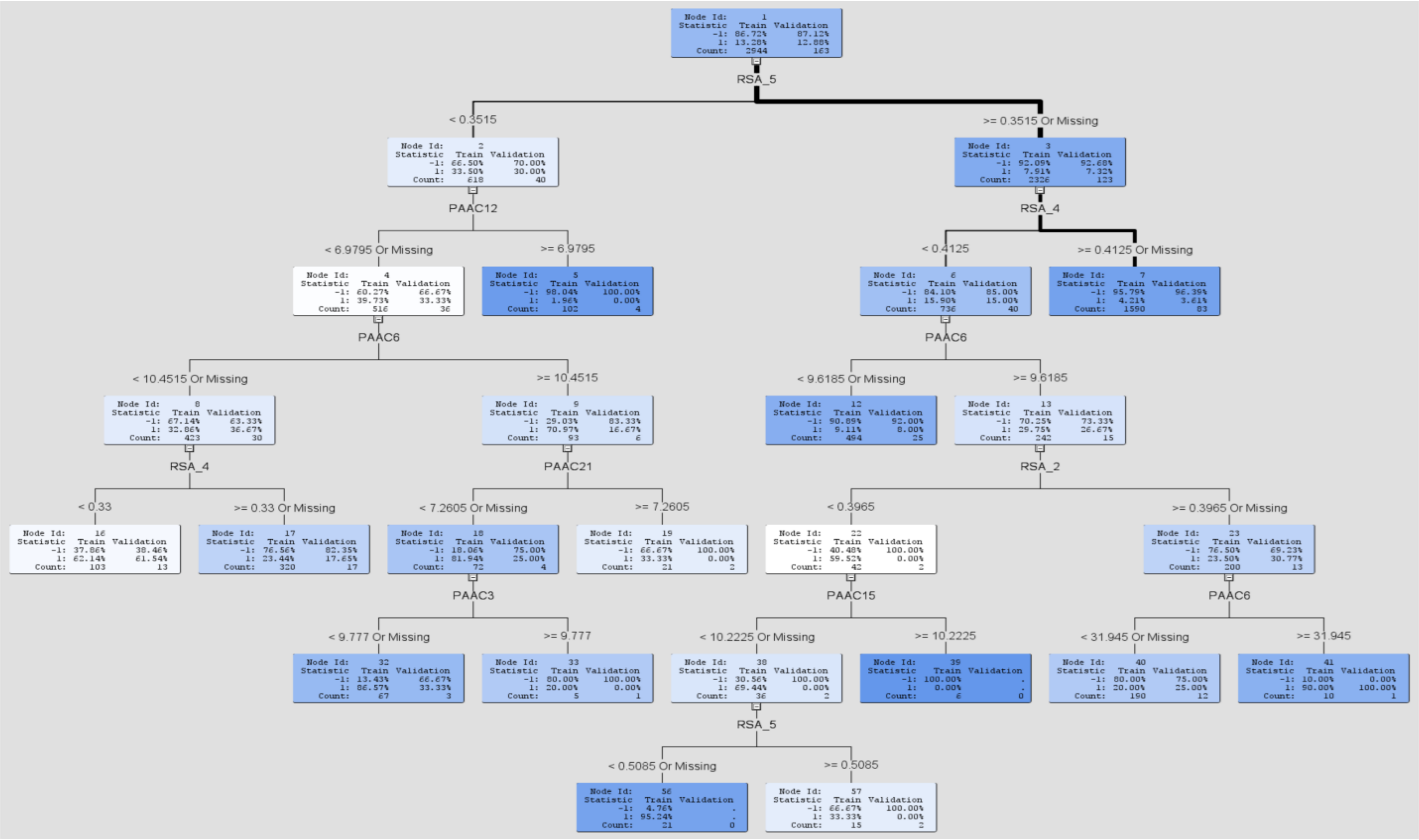

Figure S7. Decision tree of Impens dataset based on SA features.

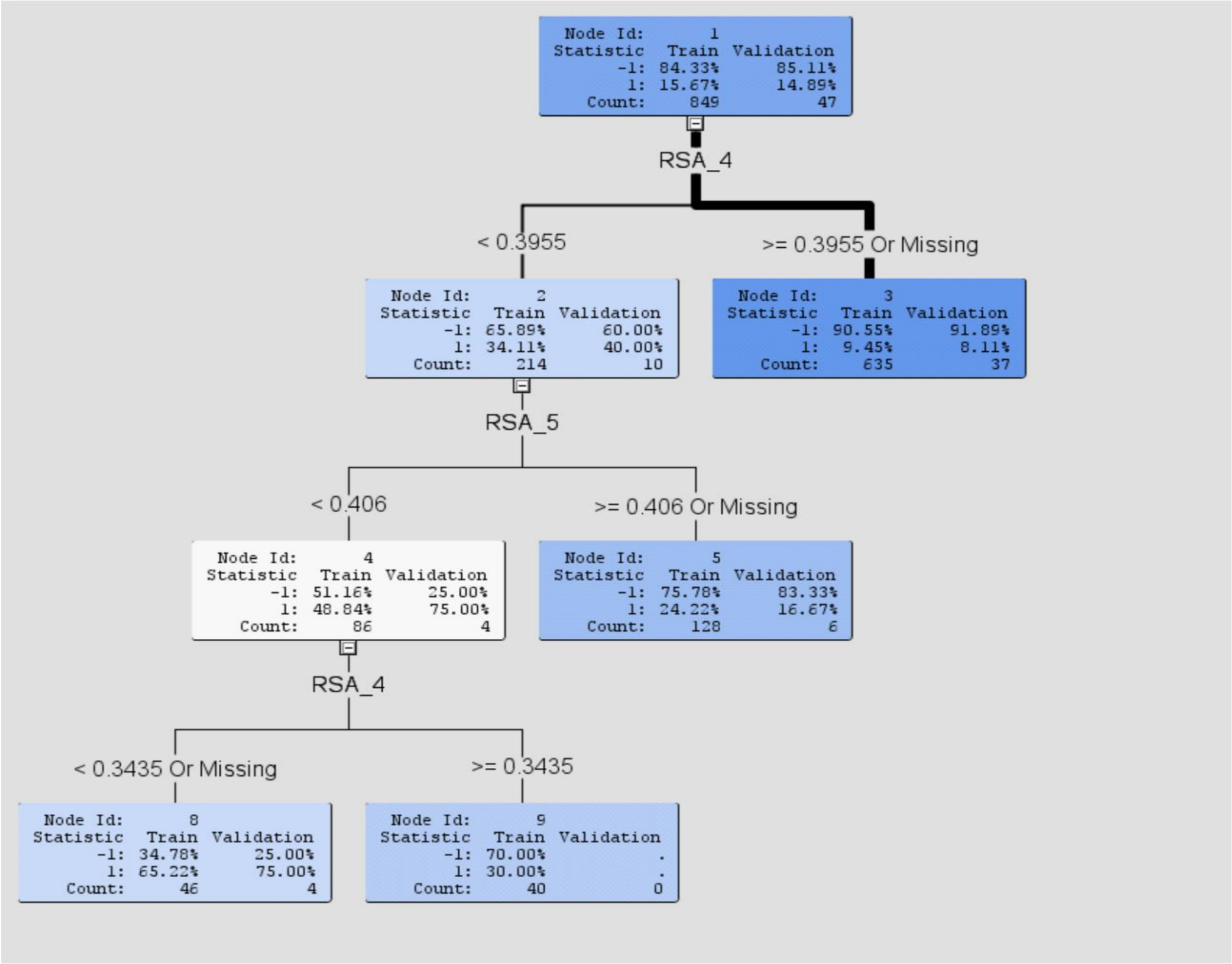

Supplement: Additional file 6: — Decision trees of the 1625, Schilling, and Impens datasets. (PDF 464 kb) [file 12859_2016_1337_MOESM6_ESM.pdf]
